# Targeting Fibroblast-Derived Interleukin 6: A Strategy to Overcome Epithelial-Mesenchymal Transition and Radioresistance in Head and Neck Cancer

**DOI:** 10.3390/cancers17020267

**Published:** 2025-01-15

**Authors:** Xinyang Li, Hugues Escoffier, Thomas Sauter, Mahvash Tavassoli

**Affiliations:** 1 Head and Neck Oncology Group, Centre for Host Microbiome Interaction, King’s College London, Hodgkin Building, London SE1 1UL, UK; xinyang.li@kcl.ac.uk; 2 Department of Life Sciences and Medicine, University of Luxembourg, L-4370 Belvaux, Luxembourgthomas.sauter@uni.lu (T.S.)

**Keywords:** head and neck squamous cell carcinoma, IL-6, epithelial-to-mesenchymal transition, radioresistance, cancer associated fibroblasts

## Abstract

Background: Cancer-associated fibroblasts have been reported to play a central role in driving cancer progression, promoting metastasis, and conferring resistance to therapy in HNSCC. Methods: Indirect and direct co-culture models of HPV-positive and HPV-negative HNSCC cells with fibroblasts were developed to study the effect of fibroblasts on cancer cells. ELISA was used to measure IL-6 secretion in these models. To dissect the underlying signalling mechanisms, the effects of IL-6, an IL-6 receptor (IL-6R) inhibitor, a MAPK/ERK inhibitor, and a JAK/STAT inhibitor were evaluated. Epithelial-to-mesenchymal transition (EMT) was assessed by measuring EMT markers and conducting scratch assays and spheroid assays. Radioresistance was evaluated using clonogenic assays. Additionally, radioresistant (RR) cell lines were established from parental cells to examine the correlation between radioresistance and EMT. Results: Fibroblasts were found to drive EMT-like changes and heightened radioresistance in HNSCC cells through IL-6 secretion. Remarkably, these Fb-driven effects were robustly reversed using IL-6R and MAPK/ERK inhibitors in both HPV-positive and HPV-negative cell lines, whereas JAK/STAT inhibitors proved effective only in HPV-negative cells. RR cell lines exhibit a more aggressive phenotype than their parental counterparts, marked by pronounced EMT features and heightened resistance to radiotherapy. Importantly, these aggressive characteristics were substantially attenuated by targeting IL-6R or MAPK/ERK pathways. Conclusions: This study highlights the critical role of fibroblast-secreted IL-6 in driving and maintaining EMT and radioresistance in HNSCC, resulting in a more aggressive tumour phenotype. Targeting the IL-6/IL-6R/ERK pathway emerges as a promising therapeutic approach for combating CAF-driven tumour progression and improving clinical outcomes in patients with aggressive, therapy-resistant HNSCC.

## Simple Summary

Head and neck squamous cell carcinomas (HNSCCs) are highly aggressive and heterogeneous malignancies, with standard treatments often demonstrating limited efficacy. The tumour microenvironment, particularly fibroblasts, has emerged as a key contributor to metastatic potential and therapy resistance. Using co-culture models of HNSCC cells and fibroblasts, we identified the IL-6/IL-6R/ERK signalling axis as a central driver of disease progression in both HPV-positive and HPV-negative HNSCC models. In contrast, the classical IL-6/IL-6R/STAT signalling pathway was found to affect only HPV-negative HNSCC. Furthermore, radioresistant HNSCC cell lines were characterised by a stronger dependence on the IL-6/IL-6R/ERK pathway and exhibited a more pronounced epithelial-to-mesenchymal transition-like phenotype compared with their parental counterparts. Our findings, for the first time, establish the critical role of the IL-6/IL-6R/ERK pathway in inducing and maintaining a more invasive and radioresistant HNSCC subtype. This pathway emerges as a promising therapeutic target for addressing advanced or recurrent HNSCC in clinical settings.

## 1. Introduction

Head and neck squamous cell carcinomas (HNSCCs) rank as the sixth most common malignancy globally, with approximately 890,000 new cases and 450,000 deaths reported in 2018 [1]. Tobacco use and alcohol consumption are the leading risk factors worldwide, and the concurrent misuse of these substances significantly increases the risk of developing HNSCC [2]. The five-year overall survival rate for HNSCC patients is 58%. Those with HPV-positive oropharyngeal cancer exhibit the most favourable prognosis, with an 87% survival rate, whereas patients with hypopharyngeal cancer have the lowest, at 51% [3]. Treatment strategies are tailored based on the clinical characteristics of each patient and typically include surgery, radiotherapy (RT), and chemotherapy (CT). Despite advances in targeted therapies and immunotherapy, these newer treatments have yet to show a definitive therapeutic benefit in improving clinical outcomes [4].

IL-6, a pleiotropic cytokine, serves a critical role in various biological processes, including infection, haematopoiesis, inflammation, and oncogenesis [5]. The relationship between IL-6 and clinical parameters, as well as oncological outcomes in cancers, has been extensively investigated over the past two decades [6,7]. In HNSCC, IL-6 has been shown to be markedly elevated in the saliva or blood samples from patients and independently predicts tumour recurrence, poor survival, and tumour metastasis [8,9].

Previous research on HNSCC primarily focused on the cancer cells themselves, but recent studies have emphasised the pivotal role of noncancerous cells and extracellular matrix (ECM) proteins within the tumour microenvironment (TME) [10]. Cancer-associated fibroblasts (CAFs), which are abundant in the HNSCC TME, are critical for both the formation and function of the TME. Similar to other solid cancers, the presence of CAFs in the stroma of HNSCC has been reported to predict poor outcomes and promote tumorigenesis [11,12]. They contribute through autocrine and paracrine cytokines and ECM components that form scaffolds supporting tumour growth [12]. The multifaceted role of CAFs is highlighted by their correlation with several key aspects of tumour aggressiveness, including proliferation, invasion, immune suppression, and resistance to therapy [13].

Epithelial–mesenchymal transition (EMT) is a dynamic process in cancer progression where polarised epithelial tumour cells transition to a mesenchymal phenotype. This transformation is marked by the loss of cell junction proteins such as E-cadherin and β-catenin, alongside an increase in mesenchymal markers like N-cadherin, Vimentin, and α-SMA [14]. These changes in membrane molecules and transcription factors during EMT are linked not only to tumour progression and increased metastasis in HNSCC patients, resulting in poorer prognosis [15], but also to enhanced stemness, migration, invasion, and therapy resistance [14]. Furthermore, EMT has been identified as a significant factor in therapy resistance, correlating with resistance to RT, CT, and both immune and targeted therapies [16].

CAFs are crucial for cancer cell invasion as they can either induce EMT to increase invasiveness in HNSCC cells or directly remodel the ECM [17]. EMT cells were noted to be localised to the leading edge of primary tumours in proximity to CAFs in HNSCC [18]. The interaction between CAFs and cancer cells, which facilitates EMT, often occurs through various mechanisms, primarily driven by CAFs’ altered secretome, which includes growth factors and cytokines that enhance cancer cell survival, proliferation, stemness, and therapy resistance [19].

Understanding the influence of specific factors in TME is critical for improving HNSCC treatment outcomes. In the TME, IL-6 is secreted by Fb, as well as cancer cells, immune cells, and endothelial cells [9]. IL-6 signals through its two receptors, IL-6R and gp130, triggering the downstream activation of the JAK/STAT, MAPK/ERK, and PI3K signalling pathways [20]. In HNSCC, the IL-6/JAK/STAT pathway has been shown to be a potent inducer of EMT, increasing resistance against therapies such as Cetuximab or RT and enhancing metastatic potential [21,22,23]. However, the impact of IL-6 on the MAPK/ERK pathway remains not fully understood, with only partial effects reported in mediating IL-6’s pro-invasion activity [21,22]. Furthermore, there is evidence that IL-6 overexpression is particularly prominent in HPV-negative cancers but much less so in HPV-positive tumours [24]. Our previous work has shown differential signal transduction in HPV-negative and positive HNSCCs in relation to EGFR activation [25]. Thus, we also investigated whether HNSCCs with different HPV statuses respond differently to IL-6 treatment. HNSCC patients commonly undergo neo-adjuvant or adjuvant fractionated RT [26]. To more accurately replicate clinical conditions, we developed radioresistant (RR) HNSCC cell lines to study the role of the IL-6/IL-6R/ERK pathway in maintaining EMT and radioresistance in HNSCC.

The relatively poor clinicopathological features of HNSCC raise questions about whether and how CAFs contribute to a pro-tumoural TME. To address these hypotheses, we utilised 2D and 3D co-culture models, along with RR tumour cell lines, to examine how CAF-secreted IL-6 mediates interactions between fibroblasts and tumour cells, as well as the distinct signalling pathways responsible for the differing responses of HNSCC cells based on their HPV status.

## 2. Materials and Methods

### 2.1. Reagents

Recombinant IL-6 and TGF-β were purchased from PeproTech (Altrincham, UK). Tocilizumab and BP-1-102 were purchased from Selleck. SCH772984 was purchased from Cambridge Bioscience (Cambridge, UK).

### 2.2. Cell Culture

The HPV-negative HNSCC cell lines HN30 were obtained from Andrew Yeudall at the Philips Institute of Oral and Craniofacial Molecular Biology, Virginia Commonwealth University, Richmond, Virginia, USA, and HN5 from Professor Barry Gusterson from the Department of Pathology, University of Glasgow, UK. Additionally, 1BR3 normal human Fb (passages 5–8) and packaging cells PT67 hTERT were gifts from Prof. Alan Lehmann at the Sussex Centre for Genome Damage and Stability, University of Sussex, UK. HN30, HN5, 1BR3, and immortalised 1BR3 (i1BR3) were cultured in Dulbecco’s Modified Eagles Medium (DMEM; GE Healthcare, Chalfont St. Giles, UK) supplemented with 10% foetal bovine serum (FBS), 50 mg/mL streptomycin, 100 mg/mL penicillin, and 1 mM sodium pyruvate. The HPV-positive cell lines SCC090 and SCC154 were gifts from Professor Susanne Gollin at the University of Pittsburgh (Pittsburgh, PA, USA) and were grown in MEM supplemented with 10% FBS, 100 µg/mL gentamicin, and 1× MEM non-essential amino acids. In the co-culture models, Fb and HNSCC cells were seeded at a 1:1 ratio. All cells were maintained in a 95% air, 5% CO_2_ humidified incubator at 37 °C. All cells used were subjected to mycoplasma test every 3 months and were shown to be negative.

### 2.3. Collection of Fb Conditioned Media (FbCM)

FbCM was generated from Fb cultures in serum-free MEM medium supplemented with 100 µg/mL gentamicin and 1× MEM non-essential amino acids to optimise the culture conditions for HPV-positive cells in subsequent experiments. When the cultures were approximately 70% confluent, the supernatant was collected after 48 h, and all cellular debris was removed using 0.2 µm filters. The number of cells present in these cultures at the time of collection was 892,000 (±16,250) (mean ± SEM) per T25 flask.

### 2.4. Establishment of Immortalised 1BR3 Cells

The immortalised cells were generated following the established protocol [27]. Briefly, PT67 hTERT cells spontaneously produce the pBABE-puro-hTERT retrovirus. Fresh culture supernatants of PT67 hTERT were collected 24 h after transfection and then filtered with 0.45 µm syringes. 1BR3 cells were infected with these fresh filtered viral supernatants in the presence of 4 µg/mL polybrene (Merck, Darmstadt, Germany) for 24 h over three consecutive days. Infected cells were selected with puromycin. The hTERT-transformed 1BR3 cells were named i1BR3 cells.

### 2.5. Establishment of Isogenic Radioresistant HNSCC Cells

Radiation-resistant HN30 (HN30RR), HN5 (HN5RR), and SCC090 (090RR) were derived from parental HNSCC cells after repeated exposure to a Nordion GC-1000S v2.9 cell irradiator, which uses a caesium source at a dose rate of 250 ± 0.59% Gy/h. The HPV-negative HNSCC cells received 4 Gy 15 times. The HPV-positive HNSCC cells received 2 Gy two times and then 4 Gy 14 times. Radioresistant cells were maintained with 4 Gy of irradiation weekly and used for no more than 2 months to preserve their normal phenotype and behaviour [28].

### 2.6. Immunoblotting

Cells were lysed, and proteins were separated with electrophoresis and subsequently transferred to nitrocellulose membranes, as previously described [29]. Antibody dilutions and incubation times followed the manufacturer’s instructions. Protein expression was detected using a luminescence chemical imaging system with an ECL reagent. The primary antibodies used included alpha-tubulin (Sigma-Aldrich, St. Louis, MO, USA) at a concentration of 1:7000 and E-cadherin, phospho-ERK1/2, phospho-STAT3 (Tyr705), and total-STAT3 (Cell Signaling Technology, Beverly, MA, USA) at 1:1000. Secondary HRP-coupled anti-rabbit and anti-mouse antibodies were obtained from Fisher Scientific (Loughborough, UK) and Sigma-Aldrich (St. Louis, MO, USA), respectively, and used at a concentration of 1:7000.

### 2.7. Immunofluorescence

Immunofluorescence (IF) was conducted to assess the expression levels of N-cadherin and Vimentin as previously described [30]. Briefly, 20,000 to 30,000 cells were seeded into eight-chamber slides, fixed with 4% paraformaldehyde for 20 min, and permeabilised with Triton X-100 for 10 min. Following blocking with 1% BSA, the cells were incubated overnight at 4 °C with N-cadherin (1:200, #13116, Cell Signaling Technology) or Vimentin (1:200, #5741, Cell Signaling Technology) antibodies. This was followed by incubation with the appropriate secondary antibody and counterstaining with DAPI for visualisation. Images were captured using Leica THUNDER Imager Tissue (Leica Microsystems GmbH, Wetzlar, Germany) at 60× magnification. Fluorescence intensities were measured using ImageJ 1.53k.

### 2.8. MTT Assay

Cell survival was assessed using the 3-(4,5-dimethylthiazol-2-yl)-2,5-diphenyltetrazolium bromide (MTT) cell viability assay, as previously described [25]. The MTT assay, widely used to evaluate cytotoxicity, directly measures cellular metabolism and provides an indirect assessment of cell viability. The optical density was recorded at a wavelength of 570 nm using a Tecan Infinite F50.

### 2.9. GFP Labelling of Cells

Cells were transfected with the pEGFP-N1 plasmid using jetPRIME transfection reagent (Polyplus, London, UK) for a period of 24 h. Post-transfection, cells underwent selection using puromycin at a concentration of 1 µg/mL. Clones that survived were expanded and utilised in further experiments.

### 2.10. Scratch Assay

A total of 6 × 10^6^ cells were seeded into six-well plates until 100% confluence was achieved. Scratch assays were carried out as previously described [31]. Briefly, after serum starvation for 16 to 24 h, the area between the scratches was imaged immediately after creating the scratch and again at 18 or 24 h later, depending on the cell line. The percentage of scratch area closure between the two time points was calculated and expressed as a percentage of the closure of the initial scratched area. Images were captured using Leica THUNDER Imager Tissue (Leica Microsystems GmbH, Wetzlar, Germany) at 40× magnification. The scratch closure was measured using ImageJ 1.53k (Rasband, W.S., ImageJ, U. S. National Institutes of Health, Bethesda, MD, USA).

### 2.11. Spheroid Assay

**Figure 1:**
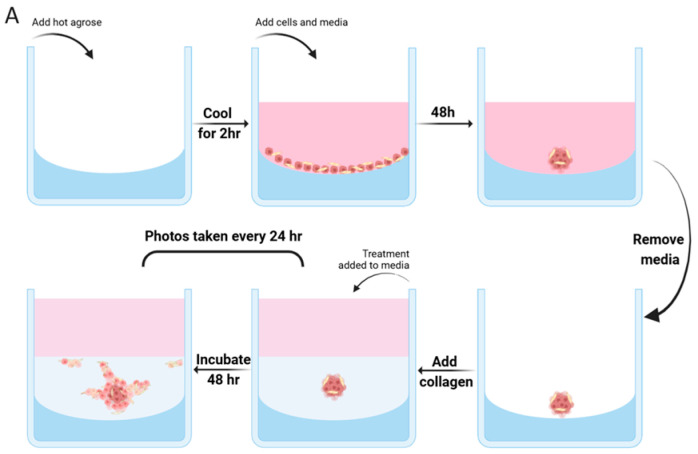

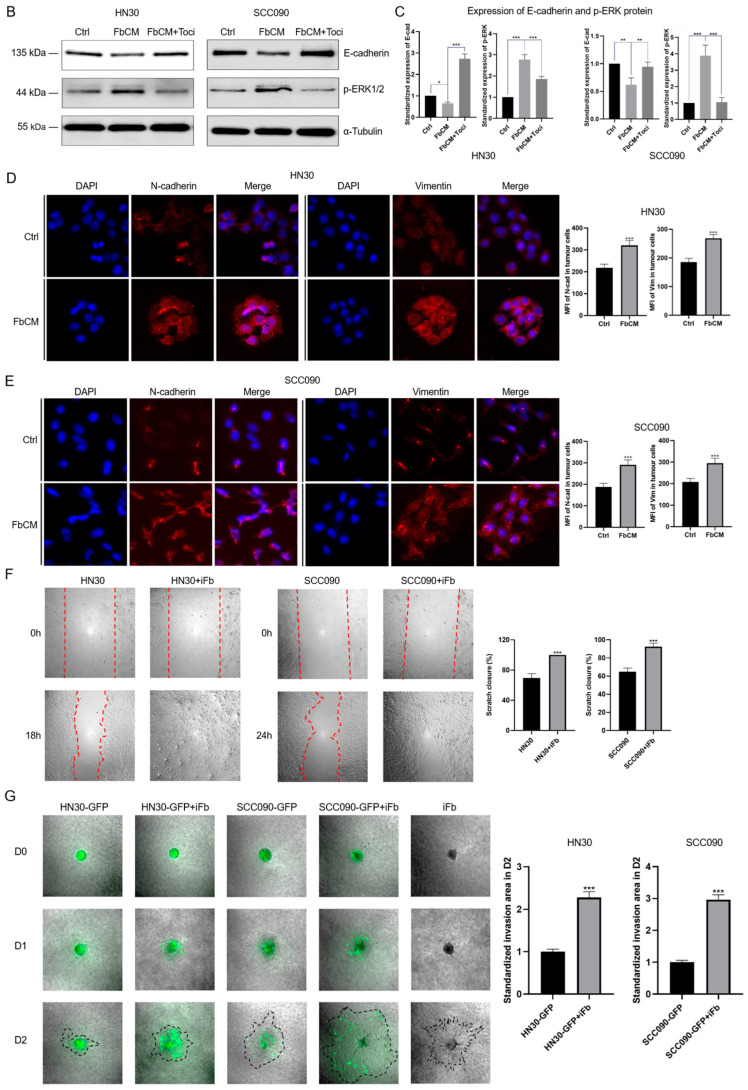
Indirect and direct interactions between HNSCC cells and Fb induce EMT phenotype and changes in HNSCC cell invasion. (**A**) Flowchart of spheroid assay for 3D invasion analysis. (**B**) Supernatants from i1BR3 cells were used to incubate both HPV-negative HN30 and HPV-positive SCC090 cells for 24 h. Tocilizumab (1 μg/mL) was also added to FbCM-treated samples. The expression of E-cadherin and p-ERK was analysed by Western blotting, with tubulin as a loading control. This immunoblot is representative of 3 independent experiments. (**C**) Quantification of three experiments involving HN30 and SCC090 cells is shown in a bar chart. Band intensities were adjusted for the intensities of the loading control bands and then normalised using the control value, one-way ANOVA (* *p* < 0.05, ** *p* < 0.01, *** *p* < 0.001). The expression of mesenchymal markers N-cadherin and Vimentin in HN30 cells (**D**) and SCC090 cells (**E**), with or without FbCM treatment, was assessed by indirect immunofluorescence, all at 60x magnification and quantification of the fluorescence intensity by independent *t* test (*** *p* < 0.001). (**F**) Cell migration of HN30 and SCC090 cells was assessed after treatment with control media or FbCM. Representative images were taken at 0 and 18 or 24 h, all at 40× magnification. Data are presented as the mean percentage of scratch area closure over the respective time periods, standardised to control values, independent *t* test (*** *p* < 0.001). (**G**) Representative images of spheroids in mono-culture of GFP-labelled HN30 or SCC090 cells and co-culture with i1BR3 cells, all at 40× magnification. Data are presented as the mean fold change of invasion area at 48 h relative to 0 h, standardised to the control group values, and displayed in a bar chart, independent *t* test (*** *p* < 0.001). Ctrl, control group; FbCM, conditioned media from i1BR3 Fb; FbCM + Toci, conditioned media from i1BR3 Fb plus Tocilizumab (1 μg/mL). All experiments were conducted independently in triplicate.

The spheroid assay was modified based on previously described methods [32]. A 1% agarose solution was used to coat 96-well tissue culture plates, creating a low-attachment surface to facilitate spheroid formation. Each well was seeded with 3000 cells and incubated for 48 h. After removing the old media, a pre-cooled collagen mixture was carefully added over the spheroids. The plate was then transferred to a 37 °C incubator to allow the collagen layer to solidify. Specific treatments were subsequently applied on top of the solidified collagen layer using the complete medium. Phase contrast images were captured at 0, 24, and 48 h using a Leica THUNDER Imager Tissue (Leica Microsystems GmbH, Wetzlar, Germany) at 40× magnification. The largest invaded area was quantified in 2D using ImageJ software (version 1.53k). Leica A flowchart illustrating the development of spheroid co-culture models is presented in Figure 1A.

### 2.12. Clonogenic Assay

The radiosensitivity of HNSCC cells was evaluated using a clonogenic assay, as previously described [25]. Briefly, drug-pre-treated cells were subjected to 4 Gy irradiation, and 3000 cells were subsequently plated in each 6 cm dish. Post-irradiation, the cells were incubated at 37 °C for 10–14 days to allow for colony formation. The dishes were then washed and stained with Coomassie Blue R350 for 30 min, and the colonies were counted and analysed.

### 2.13. ELISA

IL-6 levels in conditioned media were quantified using the ELISA MAX™ Deluxe Set Human IL-6 from BioLegend (430504) (San Diego, CA, USA), according to the manufacturer’s instructions. Absorbance was measured at a wavelength of 450 nm on a Tecan Infinite F50. The remaining cells were counted, and the conditioned media were normalised to a concentration of 10^4^ cells/mL.

### 2.14. Analysis of Public Data

Gene expression profiles and clinical data of HNSC patients were obtained from the TCGA database in July 2024 using the R package ‘TCGAbiolinks’ (version 2.32) [33]. The samples within the TCGA dataset were stratified based on the expression levels of the IL-6 and IL-6R genes. The upper third of samples, in terms of IL-6 and IL-6R expression levels, were classified as the ‘High’ group, while the lower third was classified as the ‘Low’ group. We then created an IL-6/IL-6R combined group by selecting samples that fell into the ‘High’ category for both IL-6 and IL-6R, forming a ‘High’ group (*n* = 57). Similarly, samples classified in the ‘Low’ category for both IL-6 and IL-6R were combined to create the ‘Low’ group (*n* = 62). To compare the differentially activated pathways between these groups, a Gene Set Enrichment Analysis (GSEA) Pre-ranked analysis was conducted using the R package ‘fgsea’ (version 1.30) [34]. The genes were ranked based on *p*-value and log2FC. Kaplan–Meier survival curves were generated to compare survival outcomes between the high and low IL-6/IL-6R groups, employing the R packages ‘survival’ (version 3.7) [35] and ‘survminer’ (version 0.4.9) [36]. Additionally, Single-cell RNA-Seq (scRNA-Seq) analysis was performed to determine the localisation of IL-6, IL-6R, and IL6ST expression within the cellular components of the TME. The scRNA-Seq data generated by Choi et al. was retrieved from the Gene Expression Omnibus archive (GSE181919) [37]. Cluster annotation was conducted using the marker panel listed in Table S1 [37], and the analysis was carried out using the R package ‘Seurat’ (version 5.1) [38].

### 2.15. Statistical Analysis

All experiments were conducted independently and replicated three times. Results are presented as mean ± SEM. Statistical analyses included the independent samples. Student’s t-test was performed for comparisons between two groups and one-way ANOVA for comparisons involving three or more groups. The Kolmogorov–Smirnov test was employed to assess the normality of the data distribution. A *p*-value of less than 0.05 was considered statistically significant. All statistical analyses were performed using GraphPad Prism version 10.2.3.

## 3. Results

### 3.1. Presence of Fb Induces EMT-like Changes in HNSCC Cells

In head and neck cancer, tumour cells expressing the EMT program have been shown to be spatially localised to the leading edge, in the proximity of CAFs, and were potentially induced by stimuli from them [18]. To investigate the effect of Fb on HNSCC cell lines, HPV-negative HN30 and HN5 cells, as well as HPV-positive SCC090 and SCC154 cells, were treated with filtered conditioned media (CM) derived from i1BR3 fibroblasts (FbCMs). Western blot analysis showed that the epithelial marker E-cadherin was significantly downregulated by FbCM treatment in all four cell lines (Figure 1B,C and Figure S1A,B). Concurrently, treatment with FbCM resulted in an upregulation of p-ERK levels across all HNSCC cell lines (Figure 1B,C and Figure S1A,B), indicating activation of the MAPK/ERK pathway. Immunofluorescent analysis showed that the expression levels of the EMT markers N-cadherin and Vimentin were significantly elevated in all four cell lines, further supporting that i1BR3 induces an EMT phenotype in HNSCC cells (Figure 1D,E and Figure S1C,D). To determine the effect of Fb on tumour cell proliferation, we performed MTT assays with and without FbCM treatment. Increased cell viability was observed in all four cell lines on days 4 and 5 but not on day 3 (Figure S1D). This finding is consistent with the EMT process described in the literature, which typically shows a lower proliferation rate but increased stemness characteristics [39]. Next, the effect of directly co-culturing Fb and tumour cells was evaluated using 2D scratch assays and 3D spheroid assays. In both assays, HPV-negative and HPV-positive HNSCC cells were seeded in the presence of i1BR3 at a ratio of 1:1. The addition of i1BR3 to the HNSCC cells resulted in a significantly enhanced migration ability (Figure 1F and Figure S1F) and invasion ability of HNSCC into the collagen gel (Figure 1G and Figure S1G). These results suggest that the presence of FbCM or Fb themselves induces an EMT-like phenotype and behavioural changes in both HPV-negative and HPV-positive HNSCC cells.

### 3.2. IL-6 Induces Higher EMT and Radioresistance in HNSCC Cells

**Figure 2:**
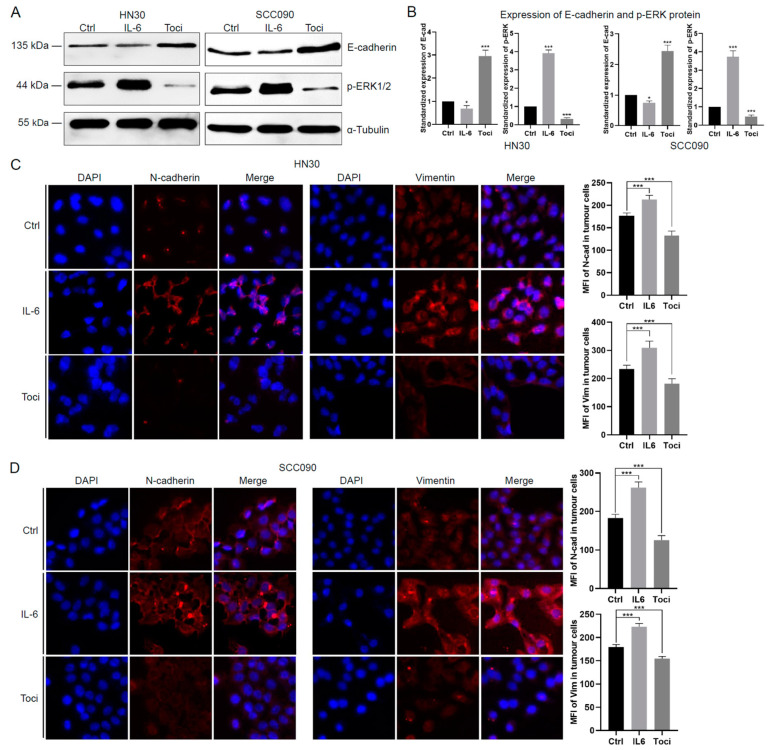

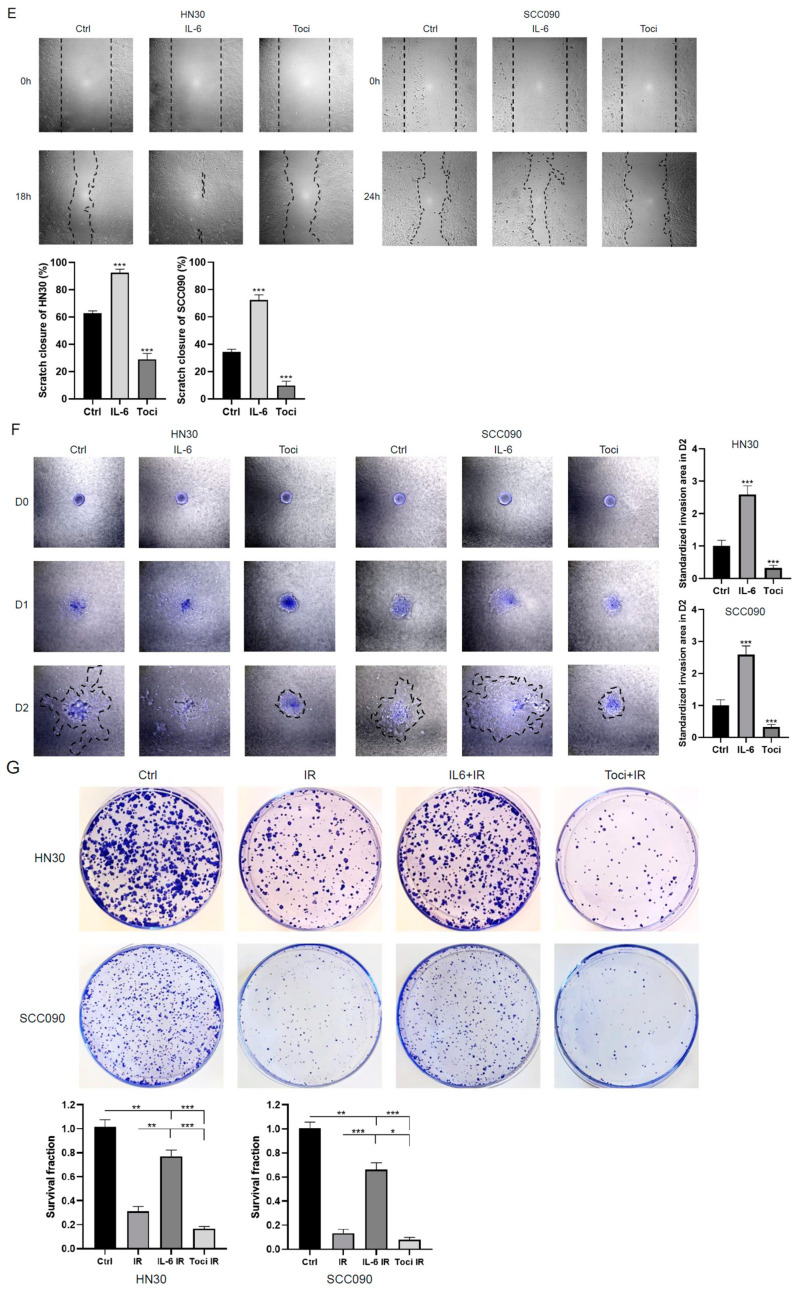
IL-6 induces EMT phenotype and enhances migration, invasion, and radioresistance in HNSCC cells. (**A**) HPV-negative HN30 and HPV-positive SCC090 cells were treated with IL-6 (10 ng/mL) or tocilizumab (1 μg/mL) for 24 h. The expression of E-cadherin and p-ERK was analysed by Western blotting, with tubulin as a loading control. This immunoblot is representative of 3 independent experiments. (**B**) Quantification of expression levels in HN30 and SCC090 cells is shown in a bar chart. Band intensities were adjusted for the intensities of the loading control bands and then normalised using the control value, one-way ANOVA (* *p* < 0.05, *** *p* < 0.001). The expression of mesenchymal markers N-cadherin and Vimentin in HN30 (**C**) and SCC090 (**D**) cells, treated with IL-6 (10 ng/mL) or tocilizumab (1 μg/mL) for 24 h, was assessed by indirect immunofluorescence. All images were taken at 60x magnification. Quantification of these experiments is presented in a bar chart, one-way ANOVA (*** *p* < 0.001). Cell migration (**E**) and invasion (**F**) were evaluated using scratch assays and spheroid assays on HN30 and SCC090 cells treated with IL-6 (10 ng/mL) or tocilizumab (1 μg/mL). Data are presented as the mean percentage of scratch area closure and mean fold change of invasion area over the respective time periods, standardised to control values, one-way ANOVA (*** *p* < 0.001) (**G**) Clonogenic assay of HN30 and SCC090 cells pre-treated with IL-6 (10 ng/mL) or tocilizumab (1 μg/mL) for 24 h. Quantification of the survival fraction was normalised to the plating efficiency of non-irradiated controls, one-way ANOVA (* *p* < 0.05, ** *p* < 0.01,*** *p* < 0.001). Ctrl, untreated control group. IR, irradiation at 4 Gy. IL-6 + IR, IL-6 (10 ng/mL) treatment prior to 4 Gy irradiation. Toci + IR, tocilizumab (1 μg/mL) treatment prior to 4 Gy irradiation. All experiments were conducted independently in triplicate.

CAFs contribute to increased invasion and metastasis by producing pro-invasive molecules, remodelling the ECM, and interacting with endothelial and immune cells. Evidence suggests that several receptor-ligand interactions, typically mediated by CAF-secreted TGF-β and IL-6 [40], can induce EMT and therapy resistance in HNSCC cells. In the first part, we demonstrated that soluble molecules secreted by i1BR3 cells can trigger changes in the levels of EMT markers. HN30 and SCC090 cells were treated with TGF-β initially, and no significant differences were observed in cell migration and invasion with or without treatment (Figure S2A,B). IL-6 has been identified as a dual inducer of MAPK/ERK pathway activation and EMT in many cancers, including HNSCC [22]. HNSCC cells were subsequently treated with IL-6, which significantly upregulated the expression of p-ERK and downregulated the epithelial marker E-cadherin, similar to the effects observed with FbCM treatment (Figure 2A,B and Figure S2C,D). The selective blocking of the IL-6 receptor with tocilizumab further verified the effect of IL-6/IL-6R on the levels of E-cadherin and p-ERK in all HNSCC cell lines (Figure 2A,B and Figure S2C,D). Indirect immunofluorescence was also performed to verify the upregulation of EMT markers N-cadherin (Figure 2C and Figure S2E) and Vimentin (Figure 2D) in IL-6-treated HNSCC cells and their downregulation when treated with tocilizumab. The results indicate that IL-6 activation significantly modulates EMT markers in HNSCC. To determine if IL-6 induces similar increases in cell migration and invasion as FbCM, tumour cells were treated with either IL-6 or tocilizumab. Activation of IL-6R with IL-6 treatment led to increased migration, as demonstrated by the scratch assay (Figure 2E and Figure S2F), and enhanced invasion, as shown by the spheroid assay (Figure 2F and Figure S2G), in both HPV-negative and HPV-positive HNSCC cells. Another frequent consequence of EMT in cancer cells is the development of therapy resistance, including radioresistance [41]. Accordingly, the potential of IL-6/IL-6R activation to induce radioresistance in HNSCC cells was evaluated. Cells were treated with IL-6 and tocilizumab prior to irradiation to assess their impact on radiosensitivity. At the concentrations used, IL-6 (10 ng/mL) and tocilizumab (1 μg/mL) did not affect cell viability at 48h (Figure S2H). Radiosensitivity was reduced, and survival was enhanced using exogenous IL-6, as demonstrated by clonogenic assays, whereas tocilizumab produced the opposite effect (Figure 2G and Figure S2I). These findings indicate that IL-6 can regulate EMT markers and p-ERK expression similarly to FbCM, while blocking IL-6R with tocilizumab reverses this effect. Moreover, the results highlight the critical role of the IL-6/IL-6R signalling pathway in response to radiotherapy.

### 3.3. Effects of IL-6 on HNSCC Cells Can Be Reversed Using ERK or STAT Inhibitors

**Figure 3:**
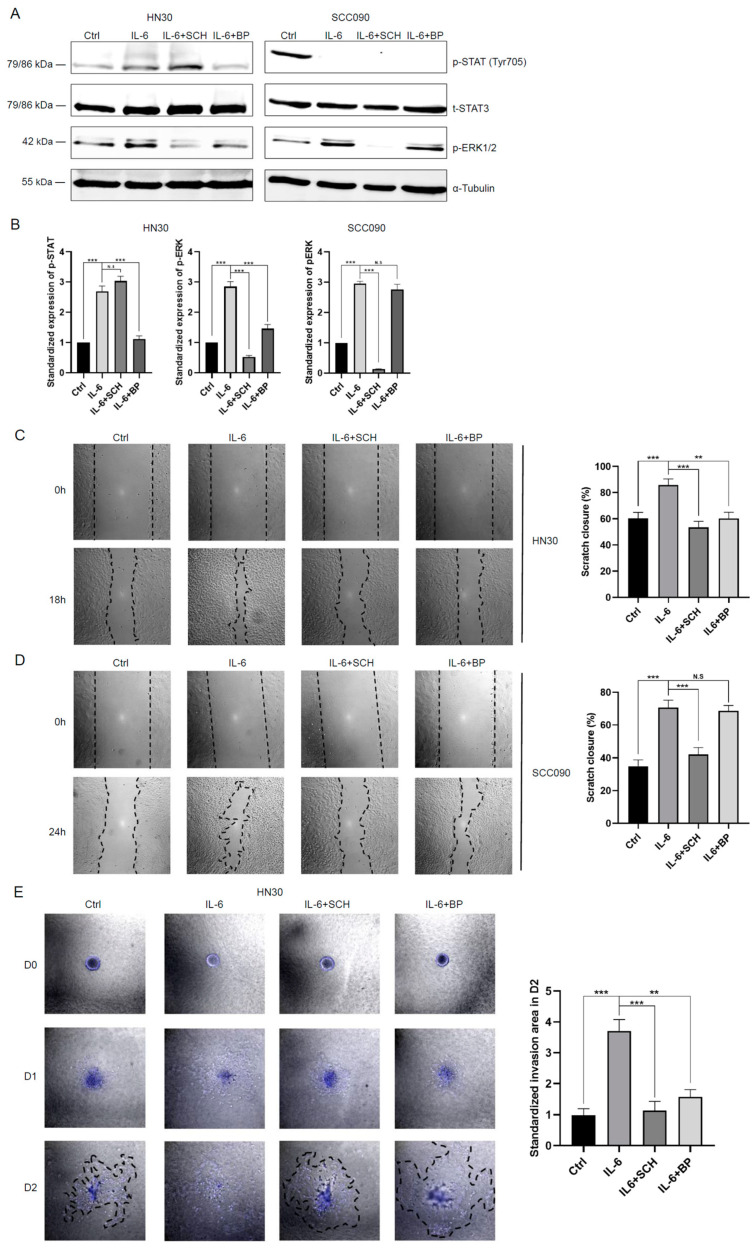

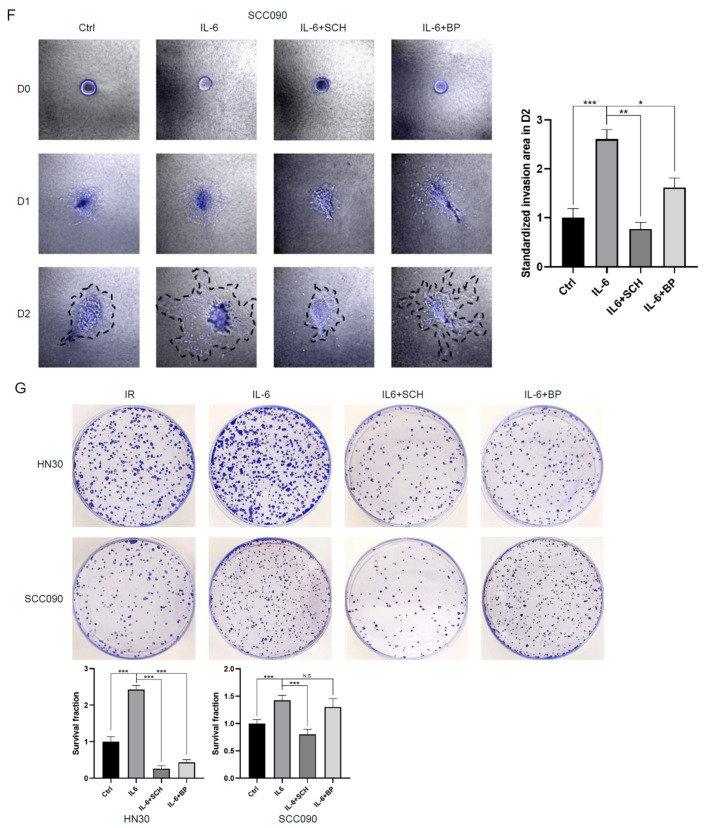
MAPK/ERK pathway is the main downstream responder triggered by IL-6 treatment to induce EMT and radioresistance. (**A**) Levels of phosphorylated STAT3, total STAT3, and phosphorylated ERK1/2 were measured in HN30 and SCC090 cells after treatment with IL-6 (10 ng/mL), with or without concurrent administration of SCH772984 (100 nM) or BP-1-102 (10 μM) for 24 h. This immunoblot is representative of 3 independent experiments. (**B**) Quantification of the Western blotting results is presented in a bar chart. Quantification of three experiments involving HN30 and SCC090 cells is shown in a bar chart. Band intensities were adjusted for the intensities of the loading control bands and then normalised using the control value, one-way ANOVA (N.S *p* > 0.05, *** *p* < 0.001). Cell migration was evaluated using a scratch assay on HN30 (**C**) and SCC090 (**D**) cells and a spheroid assay on HN30 (**E**) and SCC090 (**F**) cells. All cells were treated with IL-6 (10 ng/mL), with or without concurrent administration of SCH772984 (100 nM) or BP-1-102 (10 μM). Data are presented as the mean percentage of scratch area closure or mean fold change of invasion area over the respective time periods, standardised to the control group values, and displayed in a bar chart, one-way ANOVA (N.S *p* > 0.05, * *p* < 0.05, ** *p* < 0.01, *** *p* < 0.001). (**G**) Clonogenic assay of HN30 and SCC090 cells pre-treated with IL-6 (10 ng/mL), with or without concurrent administration of SCH772984 (100 nM) or BP-1-102 (10 μM) for 24 h. Quantification of the survival fraction of HN30 and SCC090 colonies after 4 Gy radiation was normalised to the plating efficiency of non-irradiated controls, one-way ANOVA (N.S *p* > 0.05, *** *p* < 0.001). Ctrl, untreated control group; IR, irradiation at 4 Gy. IL-6 + SCH, IL-6 (10 ng/mL) and SCH772984 (100 nM). IL-6 + BP, IL-6 (10 ng/mL) and BP-1-102 (10 μM). All experiments were conducted independently in triplicate.

IL-6 activates downstream targets via phosphorylation of ERK1/2 (p-ERK1/2) and STAT3 (p-STAT3) in cancer cells [42]. Both MAPK/ERK and JAK/STAT pathways are known to mediate EMT in multiple cancers, including HNSCC [40]. However, there has been limited research on the differential roles of the MAPK/ERK and JAK/STAT pathways between HPV-negative and HPV-positive HNSCC. At the concentrations used, ERK inhibitor SCH772984 (100 nM) and JAK/STAT inhibitor BP-1-102 (10 μM) did not impact the viability of either HPV-negative or HPV-positive cells within 48 h (Figure S2H). The impact of IL-6 and either inhibitor on the MAPK/ERK and JAK/STAT pathways in HNSCC was confirmed by using p-ERK- and p-STAT3-specific antibodies. Increased activation of ERK1/2 and STAT3 with IL-6 treatment was observed in HPV-negative HN30 and HN5 cells compared with controls. In contrast, HPV-positive SCC090 and SCC154 cells exhibited activated ERK1/2 but had reduced expression of p-STAT3 following IL-6 treatment (Figure 3A,B and Figure S3A,B). Furthermore, the inhibitors SCH772984 and BP-1-102 demonstrated effective inhibition of the MAPK/ERK and JAK/STAT pathways, respectively, in all HNSCC cell lines tested (Figure 3A,B and Figure S3A,B). To further investigate the behavioural changes following MAPK/ERK or JAK/STAT inhibition, scratch assays and spheroid assays were conducted on all HNSCC cell lines. ERK inhibitors were found to reverse the pro-aggressive effects of IL-6 in all HNSCC cell lines tested, regardless of HPV status (Figure 3C–F and Figure S3B–E). Surprisingly, following IL-6 treatment, the JAK/STAT inhibitor significantly reduced aggressive behaviour only in the HPV-negative HN30 and HN5 cell lines (Figure 3C,E and Figure S3B,D). In contrast, the changes in migration and invasion were either non-significant or marginal in BP-1-102-treated HPV-positive HNSCC cell lines (Figure 3D,F and Figure S3C,D).

MAPK/ERK [43] and JAK/STAT [44] pathways have been shown to be important modulators of radiosensitivity in HNSCC. To investigate this further, four HNSCC cell lines were pre-treated with IL-6 in combination with either an ERK or STAT inhibitor before being exposed to 4 Gy of irradiation. The results from clonogenic assays revealed that IL-6-induced radioresistance was effectively neutralised using SCH772984, an ERK inhibitor, in both HPV-negative and HPV-positive cells. In contrast, the STAT inhibitor was able to reverse the radioresistance effect of IL-6 only in HPV-negative cells, with no significant effect observed in HPV-positive cells (Figure 3G and Figure S3F).

The effects of adding an ERK1/2 inhibitor or JAK/STAT inhibitor to IL-6 treatment are summarised in Figure S3G, illustrating the distinct contributions of these pathways in mediating IL-6-induced enhancement of EMT behaviour and radioresistance. These findings indicate that IL-6-mediated activation of the MAPK/ERK signalling pathway plays a crucial role in enhancing tumour cell migration, invasion, and radioresistance in HNSCC. Conversely, the JAK/STAT3 signalling pathway appears to be more influential in promoting an aggressive phenotype in HPV-negative HNSCC cells, whereas its impact is considerably less pronounced in HPV-positive HNSCC cells.

### 3.4. Fb Serves as a Primary Source of IL-6, Which Can Be Further Induced by Irradiation

**Figure 4:**
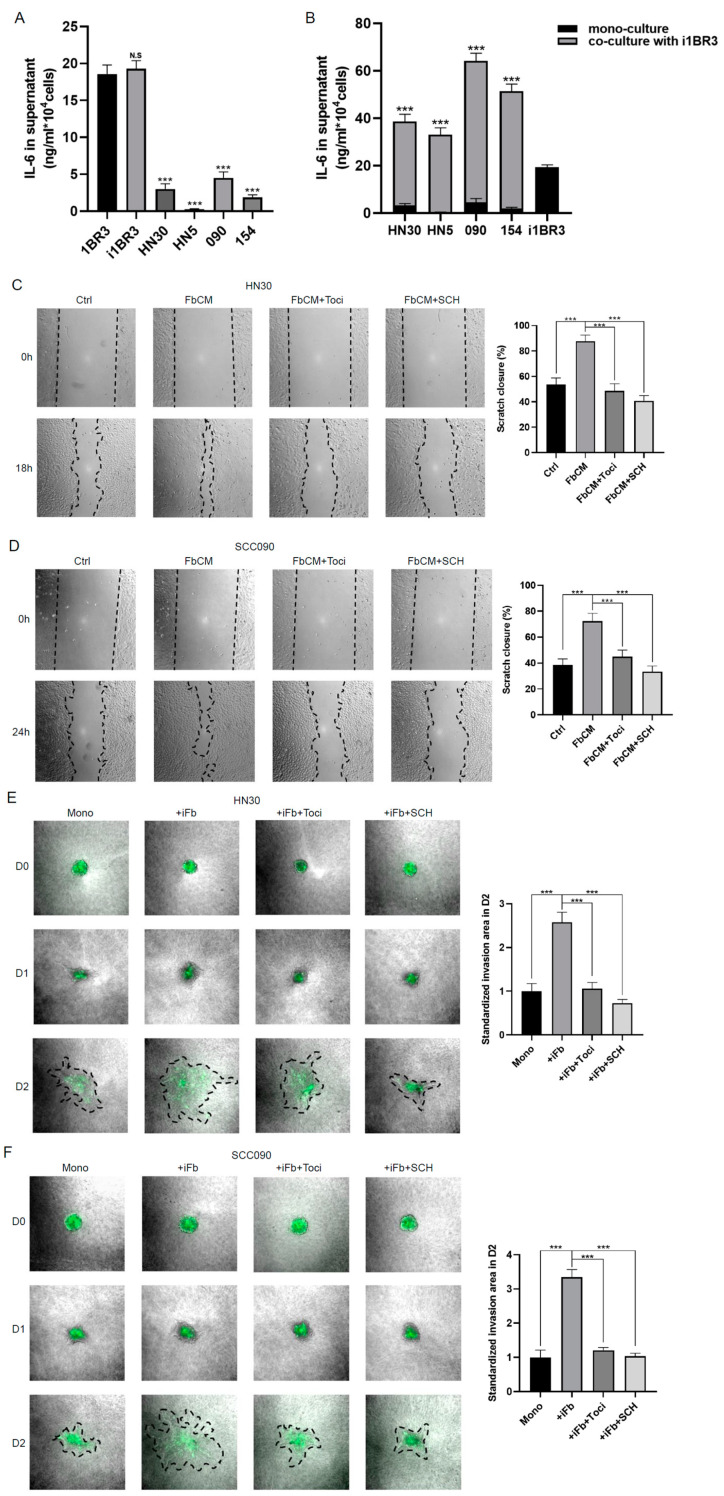

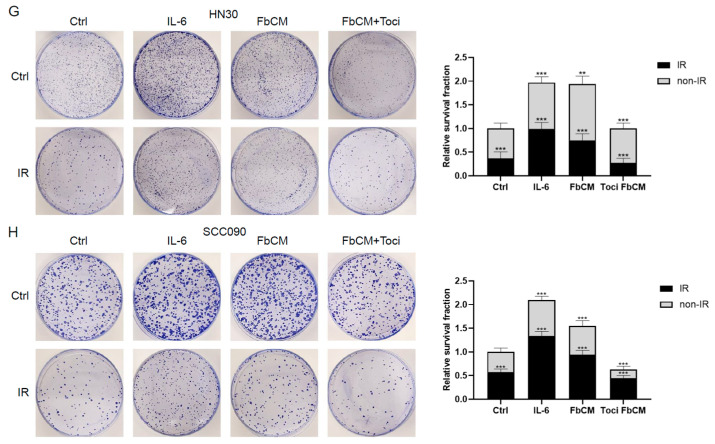
Blocking the IL-6 receptor or MAPK/ERK pathway eliminates the effects of Fb-derived IL-6 on EMT and radioresistance. Baseline secretion of IL-6 in conditioned media from mono- (**A**) and co-culture (**B**) of HNSCC and Fb cell lines was measured, one-way ANOVA (N.S *p* > 0.05, *** *p* < 0.001). Cell migration of HN30 (**C**) and SCC090 (**D**) cells was assessed after treatment with control media or FbCM. tocilizumab (1 μg/mL) or SCH772984 (100 nM) was added concurrently with FbCM. Representative images were taken at 0 and 18 or 24 h, all at 40× magnification. Data are presented as the mean percentage of scratch area closure over the respective time periods, standardised to control values, one-way ANOVA (*** *p* < 0.001). Representative images of spheroids in mono-culture of GFP-labelled HN30 (**E**) or SCC090 (**F**) cells and co-culture with i1BR3 cells, all at 40x magnification. tocilizumab (1 μg/mL) or SCH772984 (100 nM) was added to the co-culture group. Data are presented as the mean fold change of invasion area at 48 h relative to 0 h, standardised to the control group values, and displayed in a bar chart, one-way ANOVA (*** *p* < 0.001). Clonogenic assay of HN30 (**G**) and SCC090 (**H**) cells pre-treated with IL-6 (10 ng/mL) or FbCM, with or without concurrent administration of tocilizumab (1 μg/mL) for 24 h, was performed. Quantification of the survival fraction after 4 Gy radiation was normalised to the plating efficiency of non-irradiated controls, one-way ANOVA (** *p* < 0.01, *** *p* < 0.001). Ctrl, untreated control group; FbCM, conditioned media from i1BR3 Fb; IR, irradiation at 4 Gy. All experiments were conducted independently in triplicate.

Considering that IL-6 can trigger the same effects as FbCM, and given that the IL-6/IL-6R pathway is highly activated in HNSCC tumour [9], we next investigated whether Fb secreted IL-6 is causing the FbCM-induced EMT and increased radioresistance in HNSCC. To identify the source of IL-6 in our co-culture system, the baseline secretion levels of IL-6 from Fb and the tumour cell lines were assessed. ELISA results indicated that the baseline secretion of IL-6 in the CM from i1BR3 cells was comparable to that from primary 1BR3 cells and that Fb secreted IL-6 at a rate approximately five times higher than that of the tumour cell lines (Figure 4A). Additionally, changes in IL-6 secretion levels in co-culture compared with mono-culture of each cell line were measured. ELISA results demonstrated a dramatic enhancement in IL-6 secretion across all co-culture models, with HPV-positive HNSCC cells showing an even greater ability to induce higher IL-6 secretion (Figure 4B). The results indicate that IL-6 is primarily secreted by Fb, and its secretion is further enhanced in co-culture models of HNSCC cells and Fb. Irradiation and treatment with TGF-β further enhanced the secretion level of IL-6 from i1BR3 cells. The results indicate that IL-6 is primarily secreted by Fb, and its secretion is enhanced in co-culture models of HNSCC cells and Fb. Additionally, irradiation and TGF-β treatment further increase IL-6 secretion from i1BR3 cells (Figure S4A). The selective IL-6R inhibitor tocilizumab was shown to counteract the effects of FbCM, specifically reversing the downregulation of E-cadherin and the upregulation of p-ERK (Figure 1A,B). Blocking the IL-6 receptor also abrogated the pro-migration (Figure 4C,D, and Figure S4B,C) and pro-invasion (Figure 4E,F, and Figure S4D,E) effects observed with the presence of co-cultured 1BR3 cells. It is noted that the addition of SCH772984, an ERK inhibitor, to FbCM-treated models produced effects similar to those of tocilizumab in both scratch and spheroid assays (Figure 4C–F and Figure S4B–E). The colony assay demonstrated a clear increase in survival and radioresistance of HNSCC cell lines pre-treated with IL-6 or FbCM compared with control cells, with a statistically significant increase in survival fraction at a dose of 4 Gy. Conversely, tocilizumab eliminated the effects of FbCM (Figure 4G,H and Figure S4F). These results underscore the mechanistic and functional involvement of the IL-6/IL-6R and MAPK/ERK pathways in mediating changes induced by the co-culture of tumour cells and Fb.

### 3.5. Validation of Sources of IL-6 in HNSCC and Downstream Responses Through Bioinformatics Analysis of Public Data

**Figure 5:**
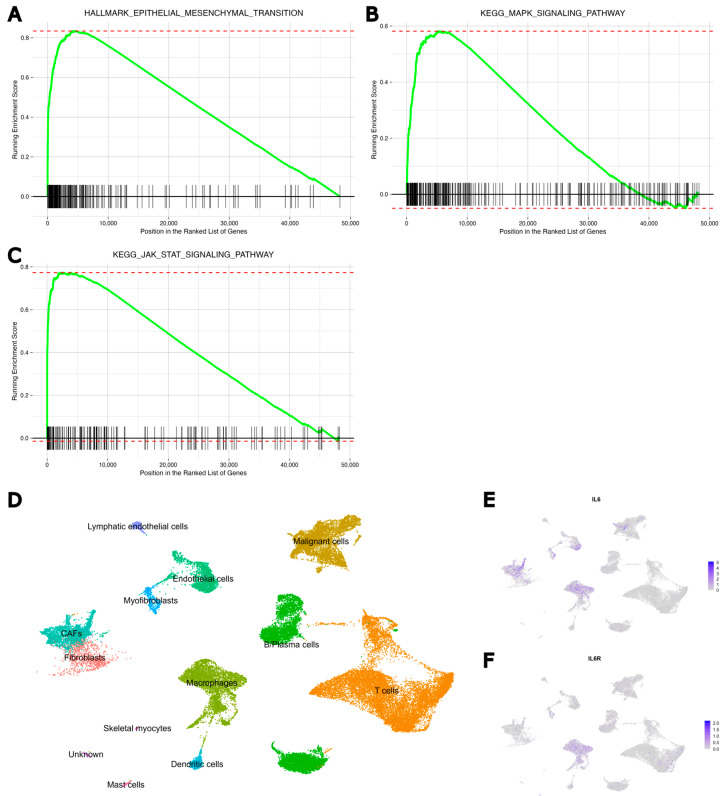
Bioinformatics analysis into IL-6 sources and downstream responses in HNSCC. GSEA enrichment plot of the (**A**) ‘Epithelial–mesenchymal transition’, (**B**) ‘MAPK signalling pathway’, and (**C**) ‘JAK/STAT signalling pathway’ gene sets. The *y*-axis represents the enrichment score (ES), while the *x*-axis depicts the genes represented in each gene set. The green line connects points of ES and genes. ES indicates the maximum deviation from zero, calculated for each gene as it is ordered in the ranked list, reflecting the degree of over-representation of a gene set at the top or bottom of the ranked gene list. The red horizontal dotted bars correspond to the minimum and maximum values observed for the enrichment score. (**D**) Uniform manifold approximation and projection (UMAP) of scRNA-Seq of all cancer-stage cells with clusters denoted by colours and labelled according to inferred cell types. (**E**) UMAP plot displaying the expression of IL6 in all cancer-stage cells. (**F**) UMAP plot displaying the expression of IL6R in all cancer-stage cells. The expression is scaled to the maximum expression of the selected gene across the HNSCC cells.

To explore the clinical significance of the IL-6/IL-6R pathway in HNSCC, differences in downstream pathway expression between ‘High’ and ‘Low’ IL-6/IL-6R groups were analysed using TCGA data from 115 primary tumour samples. Initially, HNSCC patients were divided into ‘High’ and ‘Low’ IL-6/IL-6R groups based on RNA-Seq data from TCGA, where the expression of both genes either fell in the upper third or in the lower third (see methods). Survival analysis indicated no significant difference in overall survival between the ‘High’ and ‘Low’ IL-6/IL-6R expression groups (Figure S5A). However, the downstream pathways activated in the IL-6/IL-6R ‘High’ expression group, focusing on EMT, MAPK, and JAK/STAT gene sets showed significant over-representation of these pathways in the IL-6/IL-6R ‘High’ group, with normalised enrichment scores (NESs) of 2.9, 2.0, and 2.7, respectively, and a false discovery rate (FDR) of less than 1 × 10^−10^ for all three pathways (Figure 5A–C). We also conducted a GSEA pre-ranked analysis on all gene sets in the Hallmark (Table S2) and KEGG (Table S3) databases to identify differentially represented gene sets between the ‘High’ and ‘Low’ IL-6/IL-6R groups. Notably, the EMT pathway emerged as the most significantly differentially represented gene set (FDR = 1.22 × 10^−35^), with the MAPK and JAK/STAT pathways also ranking among the top 10 most significantly represented gene sets in the KEGG analysis. These results suggest that IL-6/IL-6R pathway activation in HNSCC patients is linked to increased EMT and the activation of the MAPK/ERK and JAK/STAT pathways.

Additionally, we analysed the source of IL-6 and the distribution of IL-6R within the HNSCC TME using single-cell RNA-Seq data from patient samples [9]. The clustering was conducted, and the primary cell types within the HNSCC TME were identified (Figure 5D). The analysis revealed that IL-6 mRNA expression was primarily concentrated in cells identified as CAF, myofibroblasts, malignant cells, and macrophages (Figure 5E). In contrast, IL-6R expression was relatively low across the dataset, with expression detected in clusters identified as various cell types, including macrophages, myofibroblasts, and cancer cells (Figure 5F). The IL6ST gene, which is part of the cytokine receptor complex activated by IL-6R [15], also is expressed in malignant cells (Figure S2). In summary, the single-cell data confirmed that fibroblasts, particularly myofibroblasts and CAFs, are major sources of IL-6.

### 3.6. Radioresistant HNSCC Cell Lines Exhibit Higher EMT Status

**Figure 6:**
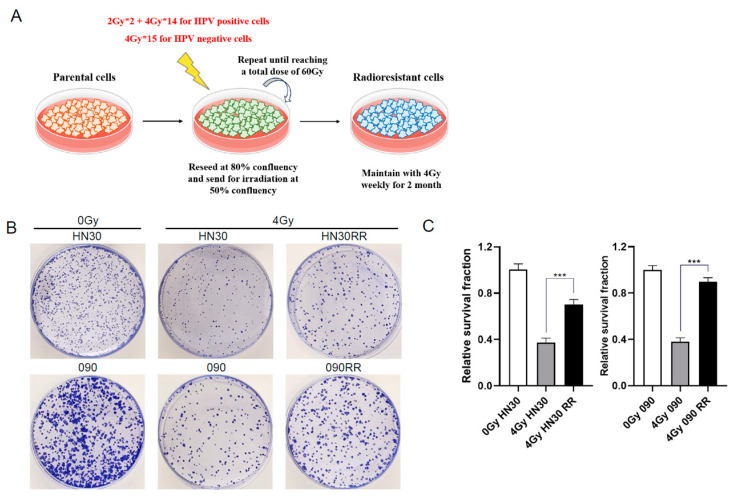

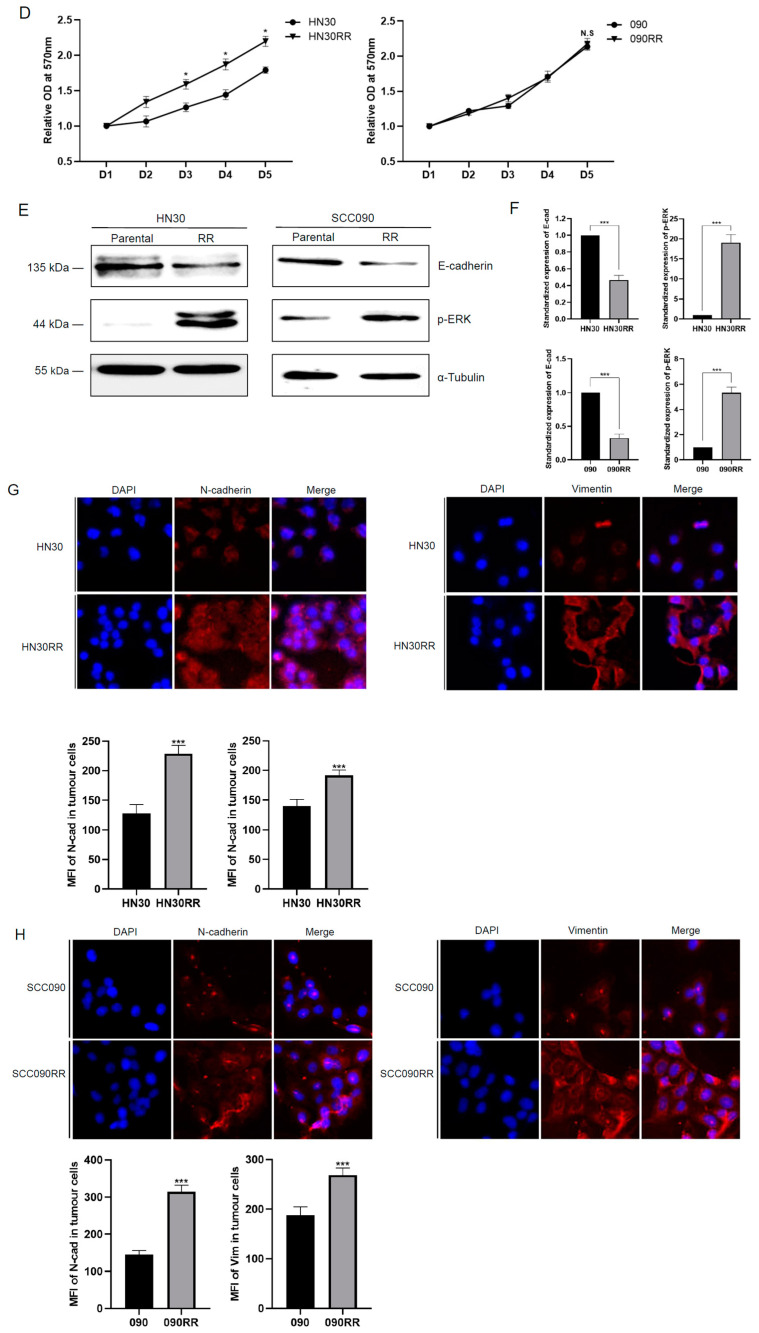

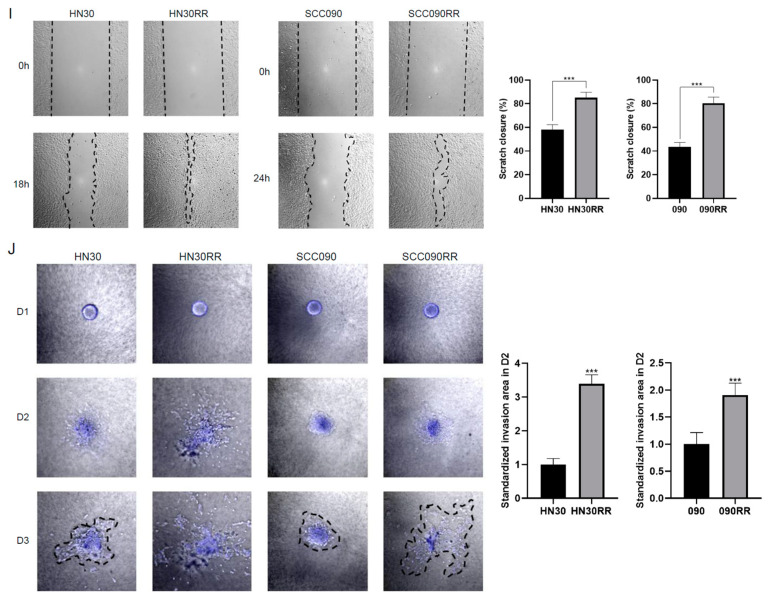
Increased expression of EMT markers, migration and invasion in RR cell lines. (**A**) The diagram demonstrates the establishment and maintenance of RR cell lines. (**B**) Clonogenic assay for HN30RR and 090RR cells compared with their parental cells following 4 Gy irradiation. (**C**) Quantification of clonogenic assay of RR compared with parental cell lines, independent *t* test (*** *p* < 0.001). (**D**) Cell proliferation measured by MTT assay on 5 consecutive days in RR cells and their corresponding parental cells, independent *t* test (N.S *p* > 0.05, * *p* < 0.05). (**E**) Expression levels of E-cadherin and phosphorylated ERK1/2 in HN30RR and 090RR and their parental counterparts as determined by western blotting. This immunoblot is representative of 3 independent experiments. (**F**) Quantification of three experiments involving HN30 and SCC090 cells is shown in a bar chart. Band intensities were adjusted for the intensities of the loading control bands and then normalised using the control value, independent *t* test (*** *p* < 0.001). Fluorescence microscopy of N-cadherin and Vimentin in HN30RR (**G**) and 090RR (**H**) cell lines and their parental counterparts, and quantification of the fluorescence intensity by independent *t* test (*** *p* < 0.001). Scratch assay (**I**) and spheroid assay (**J**) on HN30RR and 090RR cells with their parental counterparts. Data are presented as the mean percentage of scratch area closure or mean fold change of invasion area over the respective time periods, standardised to the control group values, and displayed in a bar chart, independent *t* test (*** *p* < 0.001). All experiments were conducted independently in triplicate.

Clinically, highly invasive HNSCC with nodal metastasis often results in a poor response to RT. Similarly, patients with recurrent HNSCC and radioresistant tumours frequently experience a higher rate of metastasis [45]. To investigate the relationship between acquired radioresistance and EMT, isogenic radioresistant clones HN30RR and HN5RR from the HPV-negative cell lines HN30 and HN5, and 090RR from the HPV-positive cell line SCC090 were established as described in the Methods section (Figure 6A). The clonogenic assay confirmed the successful establishment of HN30RR, HN5RR, and 090RR cells (Figure 6B,C and Figure S6A). The MTT assay revealed inconsistent changes in baseline cell viability among three pairs of RR-parental cell lines (Figure 6D and Figure S6C). This is consistent with the observation that local treatment of a primary tumour with RT can have unpredictable systemic effects on tumour growth, such as enhanced growth of distant metastases [46]. After confirming higher radioresistance in the RR cells, we investigated whether the acquisition of radioresistance occurs alongside the development of the EMT phenotype. Western blotting results indicated a decrease in E-cadherin expression and an increase in p-ERK expression in the RR cell lines compared with their parental counterparts (Figure 6E,F and Figure S6D). Consistently, IF revealed upregulated expression of the EMT markers N-cadherin and Vimentin in the RR cell lines (Figure 6G,H and Figure S6E). All RR cell lines demonstrated higher migration and invasion capabilities compared with their parental counterparts, as evidenced by scratch assays and spheroid assays (Figure 6I,J and Figure S6F,G). These results confirm the successful establishment of RR cells, which not only exhibit an EMT phenotype but also display increased aggressiveness compared with their parental counterparts.

### 3.7. IL-6R Inhibitors Reverse Radioresistance and EMT Status of Radioresistant HNSCC Cells

**Figure 7:**
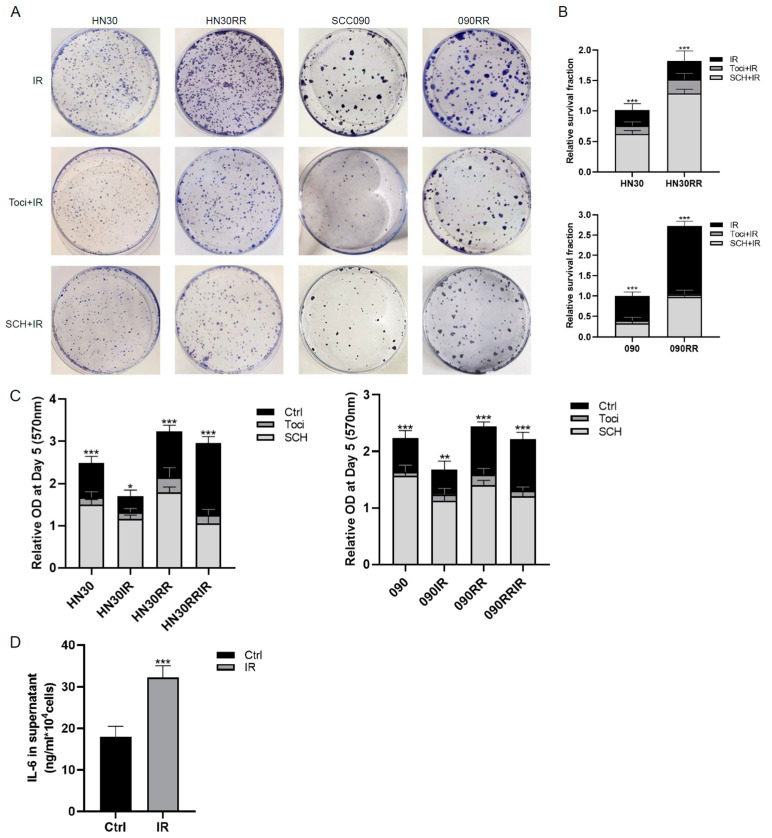

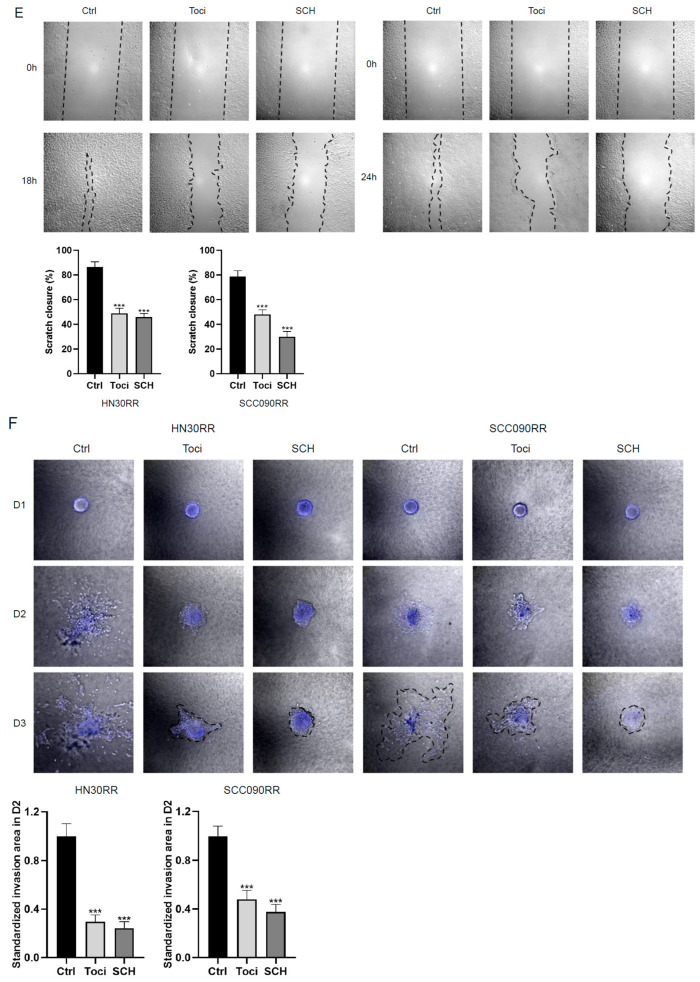
Blocking the IL-6R or MAPK/ERK pathway reverses the increased radioresistance, migration and invasion in RR cell lines. (**A**) Clonogenic assay of HN30RR and 090RR and their parental cells following 4 Gy irradiation. Cells were pre-treated with tocilizumab (1 μg/mL) or SCH772984 (100 nM) for 24 h. (**B**) Quantification of the clonogenic assay of drug-pre-treated irradiated cells with non-pre-treated irradiated cells, one-way ANOVA (*** *p* < 0.001). (**C**) Cell proliferation measured by MTT assay on Day 5 in RR cells and their corresponding parental cells with or without tocilizumab (1 μg/mL) or SCH772984 (100 nM) pre-treatment before 4Gy irradiation, one-way ANOVA (* *p* < 0.05, ** *p* < 0.01, *** *p* < 0.001). (**D**) ELISA of IL-6 in conditioned media from i1BR3 cells after a single dose of 4 Gy irradiation, independent *t* test (*** *p* < 0.001). Scratch assay (**E**) and spheroid assay (**F**) on HN30RR and 090RR cells with their parental counterparts with or without tocilizumab (1 μg/mL) or SCH772984 (100 nM) treatment. Data are presented as the mean percentage of scratch area closure or mean fold change of invasion area over the respective time periods, standardised to control group values, and displayed in a bar chart, one-way ANOVA (*** *p* < 0.001). All experiments were conducted independently in triplicate.

Since the RR cell lines exhibited changes similar to those observed with IL-6 treatment, we speculated that the alterations in RR cells are mediated through the IL-6/IL-6R/ERK pathway. Clonogenic assays on HNSCC cells pre-treated with tocilizumab or SCH772984 demonstrated that both IL-6R and ERK inhibitors act as radiosensitisers for HNSCC cells (Figure 7A,B and Figure S7A). MTT assays showed that treatment with tocilizumab or SCH772984 significantly reduced the proliferation of both RR and parental HNSCC cell lines, with a more pronounced reduction in the RR cell lines in irradiated groups (Figure 7C and Figure S7B). The ability of i1BR3 cells to secrete IL-6 was measured using ELISA, both with and without a single 4 Gy dose of irradiation. The results indicated a drastic increase in IL-6 release following irradiation (Figure 7D), suggesting that RT further stimulates the secretion of IL-6 by 1BR3 cells. To investigate whether the EMT characteristics of RR cells and their maintained radioresistance are linked through a common IL-6 pathway, scratch assays and spheroid assays were performed on RR cells. Following treatment with an IL-6R blocker or an ERK inhibitor, there was a clear reversal in the enhanced migration and invasion abilities of all RR HNSCC cell lines (Figure 7E,F and Figure S7C,D). This suggests that blocking either the IL-6 receptor or the downstream MAPK/ERK pathway effectively mitigates the aggressive traits typically associated with EMT in these cells. In summary, Fb-secreted IL-6, further potentiated by radiotherapy, activates the IL-6/IL-6R/ERK pathway, which in turn induces and maintains radioresistance and the EMT status in HNSCC cells.

## 4. Discussion

EMT is a crucial biological process that significantly contributes to tumour cell metastasis and resistance to multiple therapies, and it is frequently observed in tumours of the head and neck [47]. Multiple hypotheses explore the molecular triggers of EMT, with the most acknowledged mechanisms involving induction by secretory molecules within the TME or the mechanical properties of the ECM [39]. Despite active clinical investigations, only a few therapeutic agents targeting TME components have been tested. These include VEGF/VEGFR inhibitors aimed at anti-angiogenesis and immune checkpoint inhibitors targeting PD-1/PD-L1 and CTLA-4 pathways, yet the clinical outcomes have generally been unsatisfactory to date [4]. Specifically, despite substantial evidence demonstrating the significant pro-tumorigenic effects of CAFs in cancer, the ablation of α-SMA high Fb poses potential risks. For instance, in a murine model of pancreatic adenocarcinoma, CAF depletion resulted in more invasive tumours and increased Treg recruitment [48]. In this study, we studied the pro-tumorigenic signalling events induced by the interaction between CAFs and HNSCC cells while preserving the barrier function of the stroma.

Our data demonstrate that both conditioned media from Fb and direct co-culture of Fb with tumour cells were able to enhance typical EMT-related changes (Figure 1A–F) and radioresistance (Figure 4G,H) in HNSCC cells. The pro-EMT and radioresistance-inducing effects triggered by CAFs have been widely reported in HNSCC [19]. Secreted molecules, exosomes, mechanical stress, and metabolic products are all potent factors capable of enhancing EMT marker expression profiles, promoting aggressive behaviour, and conferring resistance to ionising radiation. This crosstalk is primarily mediated by the CAF-induced activation of Ras/MAPK, PI3K/Akt, Jak/STAT, YAP/TAZ, and Wnt signalling pathways of HNSCC cells [40]. Besides TGF-β, one of the primary candidates is IL-6, which has been found to be elevated in both serum and tumour sites in HNSCC patients and is associated with higher staging and poor prognosis [9]. The effect of IL-6 on cancer has long been recognised, with evidence showing that it induces higher proliferation and EMT changes in ovary cancers, breast cancers, and HNSCC [7,20], as well as promoting an immunosuppressive TME [49]. The hypothesis that IL-6 might be the primary trigger of the EMT program in HNSCCs is supported by the finding that a main target of the classical IL-6/IL-6R pathway, p-ERK, was upregulated following FbCM treatment and the IL-6 receptor inhibitor, tocilizumab, completely blocked the effects of FbCM on HNSCC cells (Figure 1A and Figure 4C–E). The precise source of IL-6 in the HNSCC TME is not clearly established because tumour cells, CAFs, and immune cells have all been shown to release IL-6 into the TME and even into peripheral blood [5,42]. In this research, ELISA confirmed that primary and immortalised Fb produce significantly higher levels of IL-6 compared with both HPV-negative and HPV-positive HNSCC cell lines. Importantly, our data demonstrate that IL-6 expression in Fb is likely induced through direct interaction with HNSCC cells, as IL-6 levels were further upregulated in co-cultures of HNSCC and i1BR3 cells compared with mono-cultures (Figure 4A). Furthermore, expression analysis based on scRNA data revealed that IL-6 is predominantly expressed by fibroblasts, tumour cells, and macrophages, while IL-6R and IL6ST are mainly expressed in tumour cells, CAFs, and various immune cells (Figure 5E,F). These data highlight the need for a better understanding of the exact source and modulation of EMT activators such as IL-6 in the HNSCC.

Our data strongly support the targeting of IL-6 signalling in cancer treatment. We showed that tocilizumab, an FDA-approved anti-IL-6Rα antibody for autoimmune diseases, inhibits tumour growth in both 2D and 3D HNSCC culture models. Clinically, IL-6 pathway activation, especially via the JAK/STAT pathway, is linked to aggressive cancer phenotypes and poor prognosis in ovarian, prostate, breast, and HNSCC patients [6]. IL-6 signalling inhibitors include Siltuximab (targets IL-6), tocilizumab (targets IL-6R), and Ruxolitinib (targets JAK/STAT) [50]. Among these, only tocilizumab has shown concrete evidence of improving outcomes in ovarian cancer [51] and acute myeloid leukaemia [52] by targeting the IL-6 pathway. Despite significant preclinical evidence, few IL-6 pathway inhibitors have progressed to clinical trials. Tocilizumab has been shown to effectively reduce immune-related adverse events (irAEs) and enhance the antitumour effects of anti-CTLA-4 and anti-PD-1 therapies without compromising the efficacy of immune checkpoint inhibitors (ICIs) [53].

Both the MAPK/ERK and JAK/STAT pathways are recognised as key signalling pathways in response to IL-6–IL6R binding and have been linked to a more aggressive phenotype in multiple cancers [6,7,54]. To our knowledge, our findings are the first to report the differential pro-tumorigenic role of IL-6 in HPV-negative and HPV-positive HNSCC. Both JAK/STAT3 and MAPK/ERK signalling pathways were found to mediate the observed effects of IL-6 on HPV-negative cell behaviour; however, only the MAPK/ERK pathway was activated by IL-6 treatment in HPV-positive HNSCC (Figure 3A–G).

Our bioinformatics analysis further validated these findings, with the EMT pathway ranking as the top responding pathway to IL-6, followed by the JAK/STAT pathway (Table S3). In contrast, changes in the expression of MAPK/ERK pathway genes were less associated with the higher IL-6/IL-6R expression group compared with JAK/STAT. This may be due to the unbalanced proportion of HPV-positive samples included in the TCGA database [55]. In our analysis using TCGA data, samples were not differentiated according to HPV status because only a limited number of samples had this information available (38 HPV-positive, 73 HPV-negative, and 409 with ‘unknown’ HPV status). Differentiating the samples by HPV status would have resulted in an insufficient number of samples for reliable identification of the ‘High’ and ‘Low’ IL-6/IL-6R expression groups. Our recent data identifying distinct responses to EGFR monoclonal antibody Cetuximab between-negative and HPV-positive HNSCC reinforces that these two subtypes harbour distinct tumorigenic pathways [56]. We have recently reported that the differential response of HPV-negative and HPV-positive HNSCC cells to the EGFR inhibitor Cetuximab were linked to p53 status and BRD4 expression [25,30]. It has also been reported that the extent to which both pathways are regulated by IL-6 in HNSCC can differ; in both HPV E6/E7 immortalised oral epithelial cells and HPV-negative Cal27 cells, ERK knockdown only partially affected E-cadherin expression and cell migration. In contrast, STAT3 knockdown significantly reversed IL-6-mediated upregulation of EMT markers Vimentin and snail, and attenuated IL-6-induced cell motility [21]. Conversely, another study found that STAT3 and MEK1/2 signalling inhibitors have a similar ability to suppress tumorigenesis, and the combination of both inhibitors more potently affected these phenotypes in oesophageal cancer [22]. Previous research on the correlation between IL-6 and the HPV virus has primarily focused on HPV’s effect on IL-6 secretion from cancer cells. IL-6 cytokine expression in HNSCC cell lines has been found to be upregulated by a positive HPV status [57], and in cervical keratinocytes, this upregulation is potentially induced by E6 and E7 proteins from HPV types 16 and 18 [58]. Our results, as outlined in Figure 4, showed that HPV-negative and HPV-positive HNSCC cells have comparable secretion levels of IL-6, both of which are much lower compared with Fb (Figure 4A,B). Further mechanisms in the differential response of HPV-negative and positive HNSCC cells to ERK and STAT inhibitors remain to be investigated.

Our data reinforce the clear oncogenic activity of IL-6 in both HPV-negative and HPV-positive HNSCC cells, contributing to RT resistance. RT is an essential modality in treating HNSCC, playing a key role in both early and advanced stages to preserve swallowing and speech functions. For locally advanced HNSCC, the standard of care includes concurrent RT with CT, such as cisplatin, 5-FU, and targeted therapy Cetuximab. Immunotherapy with checkpoint inhibitors is standard for recurrent or metastatic HNSCC, but its efficacy in radioimmunotherapy is limited by low PD-1/PD-L1 expression and unpredictable clinical responses [59]. Acquired radioresistance is a significant obstacle in the treatment of HNSCC. To investigate the correlation between IL-6, radioresistance, and EMT changes, we developed radioresistant cell lines from both HPV-negative and HPV-positive HNSCC cell lines. Radioresistance was successfully acquired in three RR cell lines, validating their use as an in vitro model system to characterise the expression of EMT markers, invasion behaviour, and response to IL-6 inhibitor tocilizumab. Reduced proliferation of RR cell lines compared with parental cell lines has been reported in prostate cancer [60] and breast cancer [28] and was also observed in HN5RR cells (Figure S6C). Enhanced EMT, cancer stem cell phenotypes, and activation of checkpoint proteins in acquired RR cell lines might explain this phenomenon [61,62]. Radioresistance has been linked to a more invasive phenotype in HNSCC [63], prostate cancer [61], and breast cancer [28]. However, the in vitro-acquired radioresistance of HNSCC cell lines has not been widely studied. In this research, it is linked to a more mesenchymal-like phenotype, with increased invasive and migratory potential in both 2D and 3D models. As proposed in the model in the Graphical Abstract, our RR model also demonstrated the downregulation of E-cadherin and the upregulation of Vimentin and N-cadherin, all known biomarkers of EMT [39], in all RR cell lines. The importance of IL-6 and the MAPK/ERK pathway in radioresistance and EMT was reinforced by our findings, as decreased invasiveness coupled with reversed radiosensitivity was observed after treatment with IL-6R or MAPK/ERK inhibitors. (Figure 7A–F). The correlation between the IL-6 pathway, EMT, and radioresistance has previously been established in HNSCC [64], oesophageal [65] and nasopharyngeal cancers [66]. However, our study is the first to utilise induced radioresistant cell lines to experimentally demonstrate the dual role of the IL-6/ERK pathway in EMT and radioresistance in HNSCC. Future research should aim to comprehensively elucidate the role of the IL-6/IL-6R/ERK axis within TME to facilitate the development of more effective, less toxic, and personalised therapeutic strategies for HNSCC.

Clinically, interleukin-6 (IL-6) holds recognised prognostic and monitoring value in HNSCC [9]. Elevated intra-tumoral IL-6 levels in HNSCC are associated with advanced clinical staging, therapy resistance phenotypes, increased expression of programmed death-ligand 1 (PD-L1), and other immunosuppressive characteristics [49,67]. However, given the complex interactions within the TME, careful consideration is required before integrating IL-6/IL-6R targeted therapies into clinical practice. Currently, IL-6/IL-6R pathway inhibitors, such as tocilizumab, are predominantly utilised for their anti-inflammatory effects in autoimmune diseases, cytokine release syndrome, cancer-associated cachexia, and to enhance tolerance to immunotherapy [20,68].

Preclinical studies across various cancer types have provided substantial evidence supporting the role of IL-6 in promoting cancer progression [20]. However, there remains insufficient evidence to justify the inclusion of IL-6/IL-6R-targeted monotherapy as a first-line treatment for any solid malignancy. For instance, Siltuximab, an IL-6 antagonist, has demonstrated favourable tolerability in clinical trials but has not shown significant survival benefits in patients with prostate cancer [69], clear cell renal cancer [70], or other solid tumours such as ovarian cancer [71]. Similarly, the efficacy of tocilizumab in improving clinical outcomes for epithelial ovarian cancer patients remains limited [51]. Given the intricate interplay between IL-6/IL-6R signalling and resistance to EGFR-targeted therapies [72], radiotherapy [73], chemotherapy [74], and immune checkpoint inhibitors [49], IL-6/IL-6R inhibitors may hold promise when employed in combination with existing therapeutic modalities [75]. The integration of IL-6/IL-6R inhibitors with established therapeutic modalities demonstrates promise for tackling treatment-refractory cases and deserves further investigation as part of future strategies for patient management.

## 5. Conclusions

In summary, this study reveals a pivotal interaction between HNSCC cells and fibroblasts (Fbs) mediated through IL-6/IL-6R/ERK signalling. It demonstrates that Fb-induced IL-6/IL-6R activation drives the MAPK/ERK pathway in both HPV-negative and HPV-positive cells, promoting EMT and enhancing radioresistance, while the classical IL-6/IL-6R/STAT axis plays a role only in HPV-negative HNSCC. Inhibition of either IL-6R or ERK effectively reverses EMT and re-sensitises cells to ionising radiation. These findings suggest that targeting IL-6 could be an effective approach to overcoming radioresistance and controlling disease progression in HNSCC.

## Data Availability

All data included in this research are available upon request by contact with the corresponding author.

## Supplementary Materials

The following supporting information can be downloaded at https://www.mdpi.com/article/10.3390/cancers17020267/s1, Figure S1. Indirect and direct interactions between HNSCC cells and Fb induce EMT phenotype and changes in HNSCC cell invasion, Figure S2. IL-6 induces EMT phenotype and enhances migration, invasion, and radioresistance in HNSCC cells, Figure S3. MAPK/ERK pathway is the main downstream responder triggered by IL-6 treatment to induce EMT and radioresistance, Figure S4. Blocking the IL-6 receptor or MAPK/ERK pathway eliminates the effects of Fb-derived IL-6 on EMT and radioresistance, Figure S5. K-M survival analysis of IL-6/IL-6R high and low groups and distribution of IL6ST within the HNSCC TME, Figure S6. Increased expression of EMT markers, migration and invasion in RR cell lines, Figure S7. Blocking the IL-6R or MAPK/ERK pathway reverses the increased radioresistance, migration and invasion in RR cell lines; Table S1. Table of the markers used to identify different clusters in scRNA-Seq data, Table S2. Top 5 Gene Sets in GSEA Analysis Between IL-6/IL-6R ‘High’ and ‘Low’ Groups Using the Hallmark Gene Set, Table S3. Top 10 Differential Gene Sets Between IL-6/IL-6R ‘High’ and ‘Low’ Groups Using the KEGG Gene Set.
