# Supplementary material for: Targeting Fibroblast-Derived Interleukin 6: A Strategy to Overcome Epithelial-Mesenchymal Transition and Radioresistance in Head and Neck Cancer
Source: Cancers (Basel). 2025 Jan 15;17(2):267. doi: 10.3390/cancers17020267 (PMC11763410; doi:10.3390/cancers17020267)
Supplement: Supplementary file 1 [file cancers-17-00267-s001.zip › Supplementary figure legend.pdf]

Supplementary figure legends

Fig S1. Indirect and direct interactions between HNSCC cells and Fb induce EMT phenotype and changes in HNSCC cells invasion.

(A) Supernatants from i1BR3 cells were used to incubate both HPV-negative HN5 and HPV-positive SCC154 cells for 24 hours. Tocilizumab (1 μg/ml) was also added to FbCM-treated samples. The expression of E-cadherin and p-ERK1/2 was analysed by western blotting, with tubulin as a loading control. (B) Quantification of three experiments involving HN5 and SCC154 cells is shown in a bar chart. Band intensities were normalized to tubulin and control values. The expression of mesenchymal markers N-cadherin in HN5 cells (C) and SCC154 cells (D), with or without FbCM treatment, was assessed by indirect immunofluorescence, all at 60x magnification. (E) Cell proliferation measured by MTT assay on 5 consecutive days in HN5 and SCC154 cells with or without incubation with FbCM. (F) Cell migration of HN5 and SCC154 cells was assessed after treatment with control media or FbCM. Representative images were taken at 0 and 18 or 24 hours, all at 40x magnification. Data are presented as the mean percentage of scratch area closure over the respective time periods, standardized to control values. (G) Representative images of spheroids in mono-culture of GFP-labelled HN5 or SCC154 cells and co-culture with i1BR3 cells, all at 40x magnification. Data are presented as the mean fold change of invasion area at 48 hours relative to 0 hours, standardized to the control group values, and displayed in a bar chart. Ctrl, control group; FbCM, conditioned media from i1BR3 Fb; FbCM+Toci, conditioned media from i1BR3 Fb plus Tocilizumab (1 μg/ml). All experiments were conducted independently in triplicate. P values were calculated by one-way ANOVA (B, E) or Student's t-test (C, D, F, G). Data represent the mean ± SEM. N.S, $P>0.05$, *$P<0.05$, **$P<0.01$, ***$P < 0.001$.

Fig S2. IL-6 induces EMT phenotype and enhances migration, invasion, and radioresistance in HNSCC cells.

Cell migration (A) and invasion (B) were evaluated using scratch assays and spheroid assays on HN30 and SCC090 cells treated with TGF-β1 (10ng/ml). Data are presented as the mean percentage of scratch area closure and mean fold change of invasion area over the respective time periods, standardized to control values. (C) HPV-negative HN5 and HPV-positive SCC154 cells were treated with IL-6 (10 ng/ml) or Tocilizumab (1 μg/ml) for 24 hours. The expression of E-cadherin and p-ERK was analysed by western blotting, with tubulin as a loading control. (D) Quantification of expression levels in HN5 and SCC154 cells is shown in a bar chart. Band intensities were normalized to tubulin and control values. (E) The expression of mesenchymal markers N-cadherin in HN5 and SCC154 cells, treated with IL-6 (10 ng/ml) or Tocilizumab (1 μg/ml) for 24 hours, was assessed by indirect immunofluorescence. All images were taken at 60x magnification. Quantification of these experiments is presented in a bar chart. Cell migration (F) and invasion (G) were evaluated using scratch assays and spheroid assays on HN5 and SCC154 cells treated with IL-6 (10 ng/ml) or Tocilizumab (1 μg/ml). Data are presented as the mean percentage of scratch area closure and mean fold change of invasion area over the respective time periods, standardized to control values. (H) Cell proliferation measured by MTT assay on 4 consecutive days in tumour cells treated by Tocilizumab (1μg/ml), SCH772984 (100 nM) or BP-1-102 (10 μM). (I) Clonogenic assay of HN5 and SCC154 cells pre-treated with IL-6 (10 ng/ml) or Tocilizumab (1 μg/ml) for 24 hours prior to 4Gy irradiation. Quantification of the survival fraction was normalized to the plating efficiency of non-irradiated controls. Ctrl, untreated control group. IR, irradiation at 4 Gy. IL-6+IR, IL-6 (10 ng/ml) treatment prior to 4 Gy irradiation. Toci+IR, Tocilizumab (1 μg/ml) treatment prior to 4 Gy irradiation. All experiments were conducted independently in triplicate. P values were calculated by one-way ANOVA. Data represent the mean ± SEM. N.S, $P>0.05$, *$P<0.05$, **$P<0.01$, ***$P < 0.001$.

Fig S3. MAPK/ERK pathway is the main downstream responder triggered by IL-6 treatment to induce EMT and radioresistance

(A) Levels of phosphorylated STAT3, total STAT3, and phosphorylated ERK1/2 were measured in

HN5 and SCC154 cells after treatment with IL-6 (10 ng/ml), with or without concurrent administration of SCH772984 (100 nM) or BP-1-102 (10 μM) for 24 hours. Quantification of the western blotting results is presented in a bar chart. Band intensities were normalized to tubulin and control values. Cell migration was evaluated using a scratch assay on HN5 (B) and SCC154 (C) cells, and a spheroid assay on HN5 (D) and SCC154 (E) cells. All cells were treated with IL-6 (10 ng/ml), with or without concurrent administration of SCH772984 (100 nM) or BP-1-102 (10 μM). Data are presented as the mean percentage of scratch area closure or mean fold change of invasion area over the respective time periods, standardized to the control group values, and displayed in a bar chart. (F) Clonogenic assay of HN5 and SCC154 cells pre-treated with IL-6 (10 ng/ml), with or without concurrent administration of SCH772984 (100 nM) or BP-1-102 (10 μM) for 24 hours before 4Gy irradiation. Quantification of the survival fraction of HN5 and SCC154 colonies after 4 Gy radiation was normalized to the plating efficiency of non-irradiated controls. Ctrl, untreated control group; IR, irradiation at 4 Gy. All experiments were conducted independently in triplicate. P values were calculated by one-way ANOVA. Data represent the mean ± SEM. N.S, $P>0.05$. *$P<0.05$, **$P<0.01$, ***$P < 0.001$.

Fig S4. Blocking the IL-6 receptor or MAPK/ERK pathway eliminates the effects of Fb-derived IL-6 on EMT and radioresistance.

(A) Level of IL-6 in conditioned media from i1BR3 cells after TGF-β1 (10ng/ml) or SCH772984 (100nM) for 72 hours was measured by ELISA. Cell migration of HN5 (B) and SCC154 (C) cells was assessed after treatment with control media or FbCM. Tocilizumab (1 μg/ml) or SCH772984 (100nM) was added concurrently. Representative images were taken at 0 and 18 or 24 hours, all at 40x magnification. Data are presented as the mean percentage of scratch area closure over the respective time periods, standardized to control values. Representative images of spheroids in mono-culture of GFP-labelled HN5 (D) or SCC154 (E) cells and co-culture with i1BR3 cells, all at 40x magnification. Data are presented as the mean fold change of invasion area at 48 hours relative to 0 hours, standardized to the control group values, and displayed in a bar chart. (F) Clonogenic assay of HN5 and SCC154 cells pre-treated with IL-6 (10 ng/ml) or FbCM, with or

without concurrent administration of Tocilizumab (1 μg/ml) for 24 hours, was performed. Quantification of the survival fraction after 4 Gy radiation was normalized to the plating efficiency of non-irradiated controls. Ctrl, untreated control group; FbCM, conditioned media from i1BR3 Fb; IR, irradiation at 4 Gy. All experiments were conducted independently in triplicate. P values were calculated by one-way ANOVA. Data represent the mean ± SEM. N.S, $P>0.05$, $**P<0.01$, $***P<0.001$.

Fig S5. K-M survival analysis of IL-6/IL-6R high and low groups and distribution of IL6ST within the HNSCC TME

(A) Kaplan-Meier survival analysis of overall survival stratified by IL-6/IL-6R expression levels, comparing 'High' versus 'Low' groups. Vertical steps in the curve indicate one or more death events, and censored patients are marked by vertical ticks on the curve at the time of censoring. The difference in survival between the groups was assessed using a log-rank test. (B) UMAP plot displaying the expression of IL6ST across all cancer stage cells. Cells are color-coded based on the expression levels of IL6ST, with the expression scaled to the maximum expression of IL6ST across the HNSCC cells.

Fig S6. Increased expression of EMT markers, migration and invasion in RR cell lines

(A) Clonogenic assay for HN5RR cells compared to their parental cells following 4 Gy irradiation. (B) Quantification of clonogenic assay. (C) Cell proliferation measured by MTT assay on 5 consecutive days of HN5 and HN5RR cells. (D) Expression levels of E-cadherin and phosphorylated ERK1/2 in HN5 and HN5RR as determined by western blotting. (E) Indirect immunofluorescence of N-cadherin and Vimentin in HN5 and HN5RR cells. Scratch assay (F) and spheroid assay (G) of HN5 and HN5RR cells. Data are presented as the mean percentage of scratch area closure or mean fold change of invasion area over the respective time periods, standardized to the control group values, and displayed in a bar chart. All experiments were conducted independently in triplicate. P values were calculated by One-way ANOVA (B, C) or Student's t test (E, F, G). Data represents the mean ± SEM. $*P<0.05$. $***P<0.001$.

Fig S7. Blocking the IL-6R or MAPK/ERK pathway reverses the increased radioresistance, migration and invasion in RR cell lines.

(A) Clonogenic assay of HN5 and HN5RR cells following 4 Gy irradiation with quantification. Cells were pre-treated with Tocilizumab (1 µg/ml) or SCH772984 (100 nM) for 24 hours. (B) Cell proliferation measured by MTT assay on Day 5 in HN5 and HN5RR cells with or without Tocilizumab (1 µg/ml) or SCH772984 (100 nM) pretreatment treatment. Cells were then subjected to 4Gy irradiation. Scratch assay (C) and spheroid assay (D) on HN5 and HN5RR with or without Tocilizumab (1 µg/ml) or SCH772984 (100 nM) treatment. Data are presented as the mean percentage of scratch area closure or mean fold change of invasion area over the respective time periods, standardized to control group values, and displayed in a bar chart. All experiments were conducted independently in triplicate. P values were calculated by one-way ANOVA. Data represent the mean ± SEM. *P<0.05, **P<0.01, ***P < 0.001.
