# Supplementary material for: Targeting Fibroblast-Derived Interleukin 6: A Strategy to Overcome Epithelial-Mesenchymal Transition and Radioresistance in Head and Neck Cancer
Source: Cancers (Basel). 2025 Jan 15;17(2):267. doi: 10.3390/cancers17020267 (PMC11763410; doi:10.3390/cancers17020267)
Supplement: Supplementary file 1 [file cancers-17-00267-s001.zip › Supplementary figures .pdf]

# Figure S1

Indirect and direct interactions between HNSCC cells and fibroblasts induce EMT phenotype and changes in HNSCC cells invasion

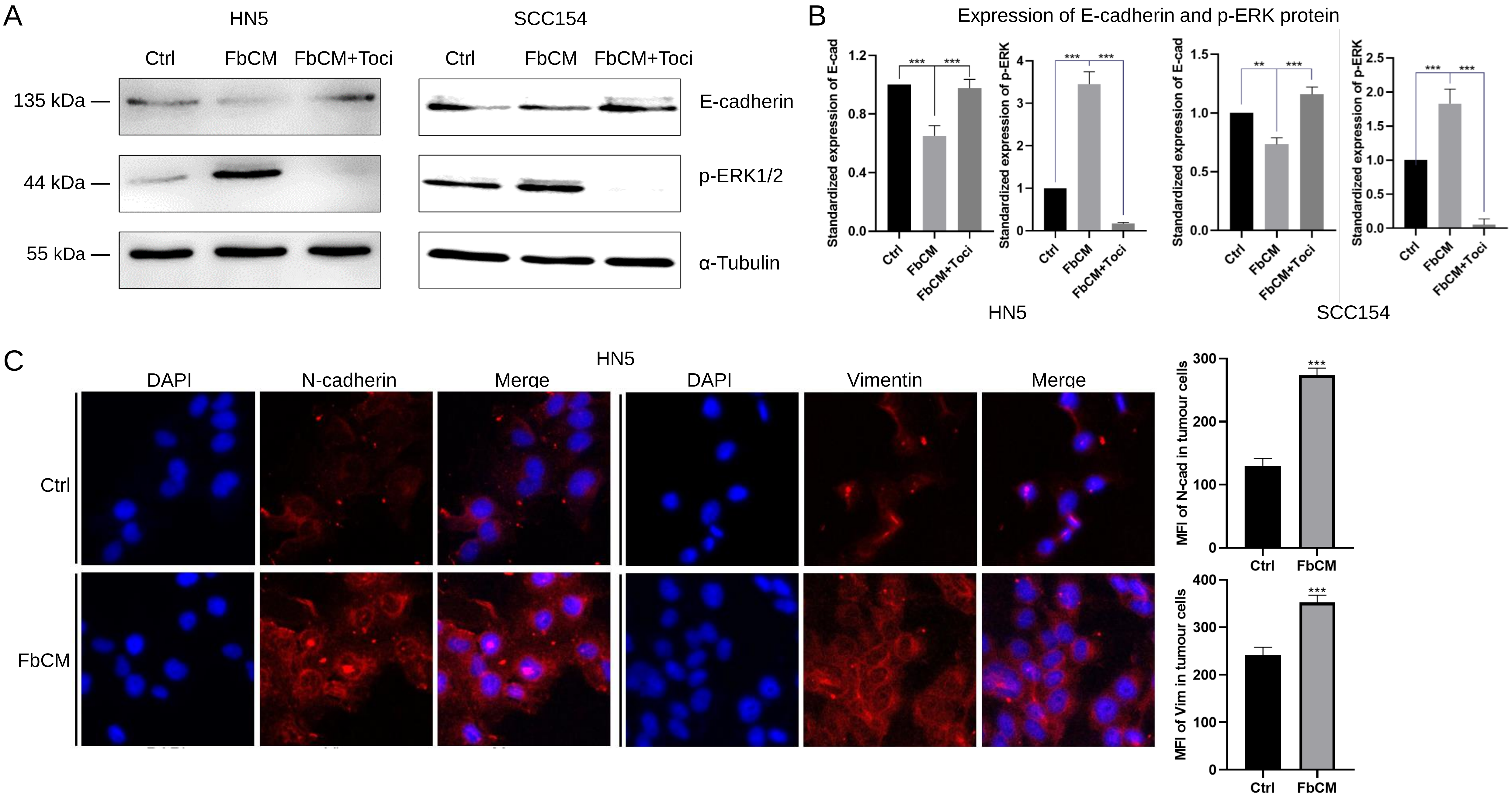
A
HN5
SCC154
Ctrl
FbCM
FbCM+Toci
135 kDa —
44 kDa —
55 kDa —
E-cadherin
p-ERK1/2
α-Tubulin
B
Expression of E-cadherin and p-ERK protein
Standardized expression of E-cad
Standardized expression of p-ERK
HN5
SCC154
C
HN5
DAPI
N-cadherin
Merge
Vimentin
Ctrl
FbCM
MFI of N-cad in tumour cells
MFI of Vim in tumour cells

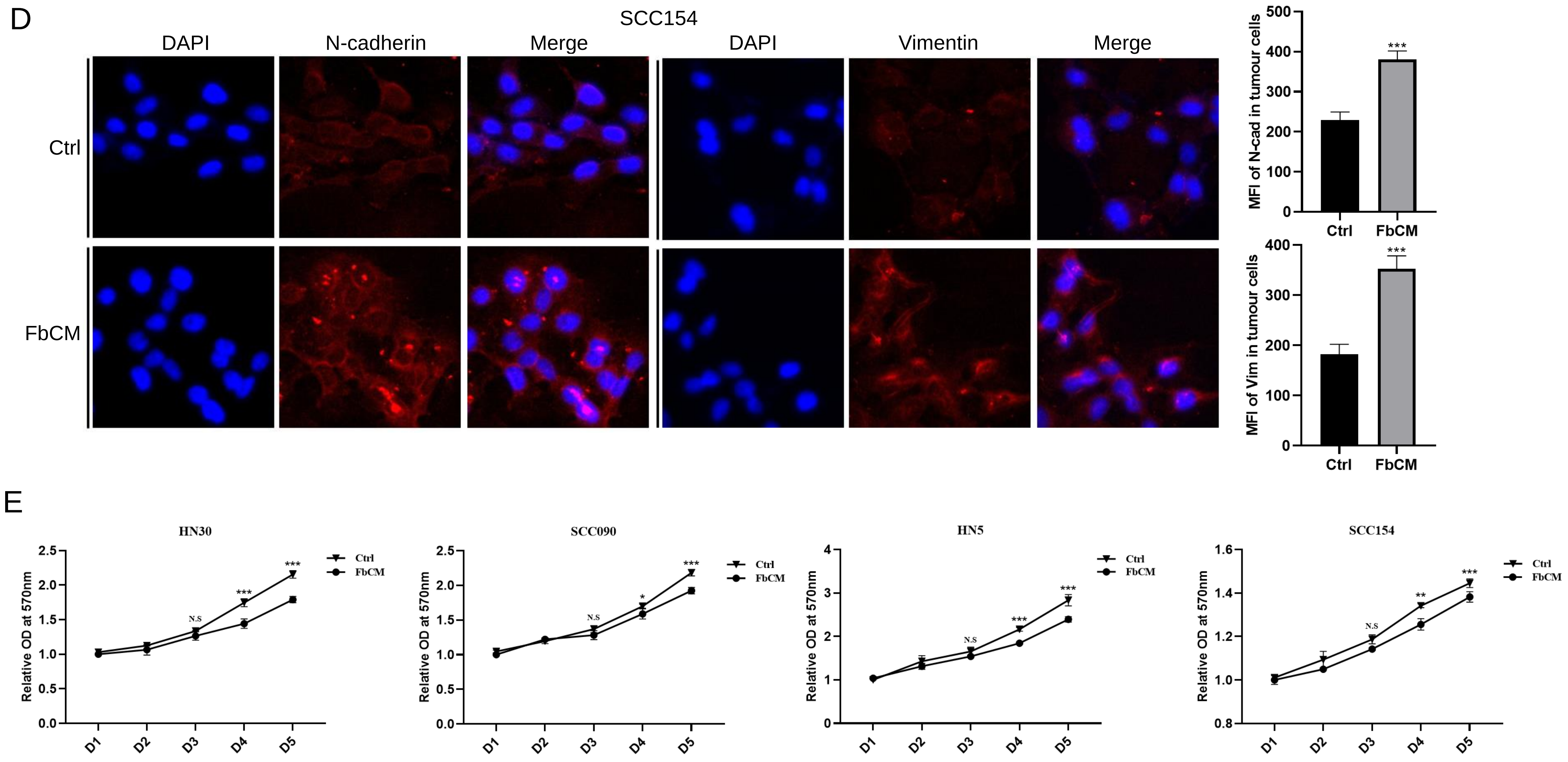

D
SCC154
DAPI
N-cadherin
Merge
DAPI
Vimentin
Merge
Ctrl
FbCM
MFI of N-cad in tumour cells
500
400
300
200
100
0
***
Ctrl
FbCM
MFI of Vim in tumour cells
400
300
200
100
0
***
Ctrl
FbCM
E
HN30
SCC090
HN5
SCC154
Relative OD at 570nm
Ctrl
FbCM
N.S
*
**
***
D1
D2
D3
D4
D5

F

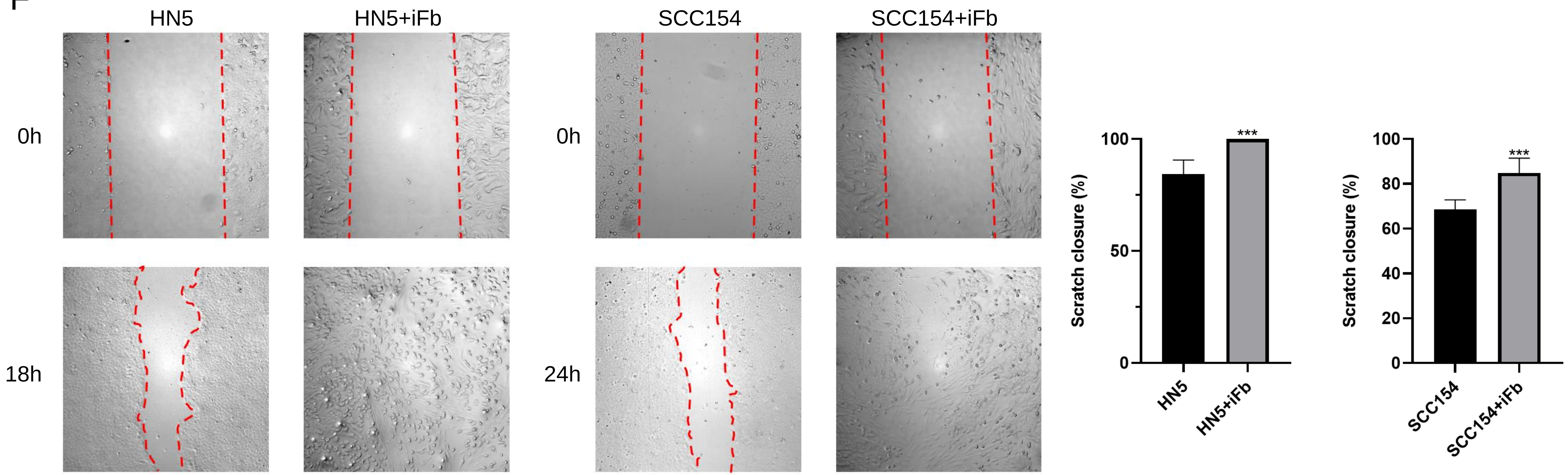
HN5
HN5+iFb
SCC154
SCC154+iFb
0h
18h
0h
24h
Scratch closure (%)
100
50
0
***
HN5
HN5+iFb
Scratch closure (%)
100
80
60
40
20
0
***
SCC154
SCC154+iFb

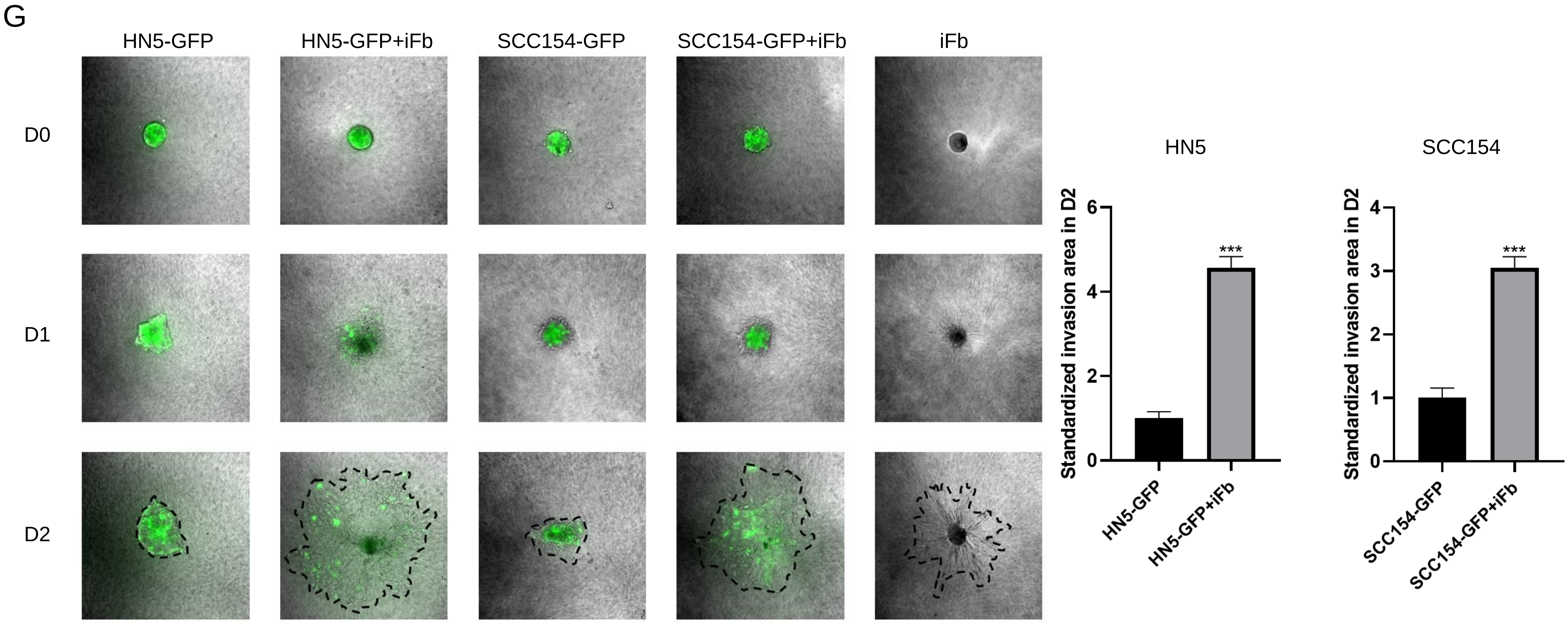
G
HN5-GFP
HN5-GFP+iFb
SCC154-GFP
SCC154-GFP+iFb
iFb
D0
D1
D2
HN5
SCC154
Standardized invasion area in D2
6
4
2
0
***
HN5-GFP
HN5-GFP+iFb
4
3
2
1
0
SCC154-GFP
SCC154-GFP+iFb

# Figure S2

IL-6 induces EMT phenotype and enhances migration, invasion, and radioresistance in HNSCC cells.

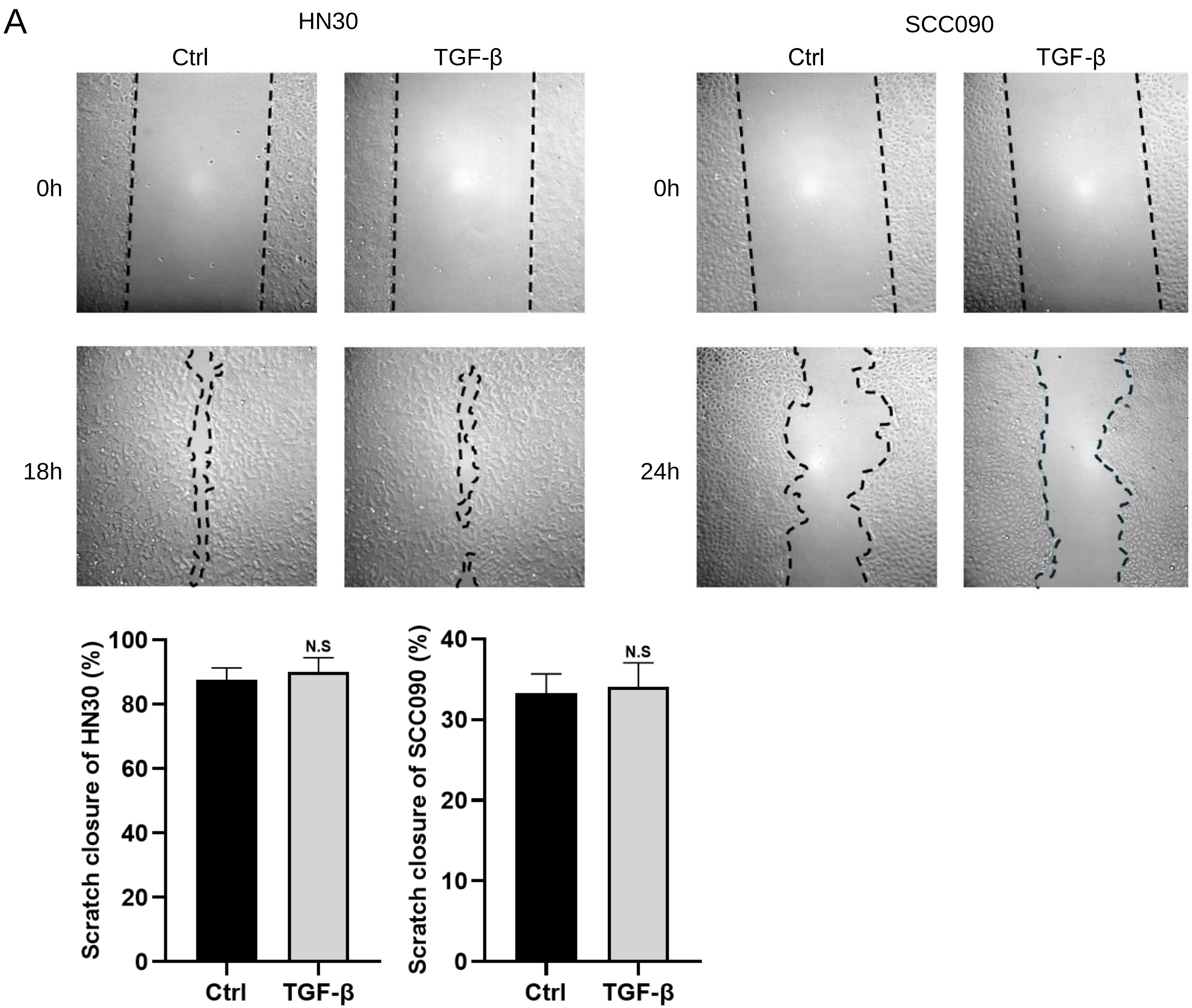

A
HN30
SCC090
Ctrl
TGF-β
Ctrl
TGF-β
0h
0h
18h
24h
Scratch closure of HN30 (%)
N.S
100
80
60
40
20
0
Ctrl
TGF-β
Scratch closure of SCC090 (%)
N.S
40
30
20
10
0
Ctrl
TGF-β

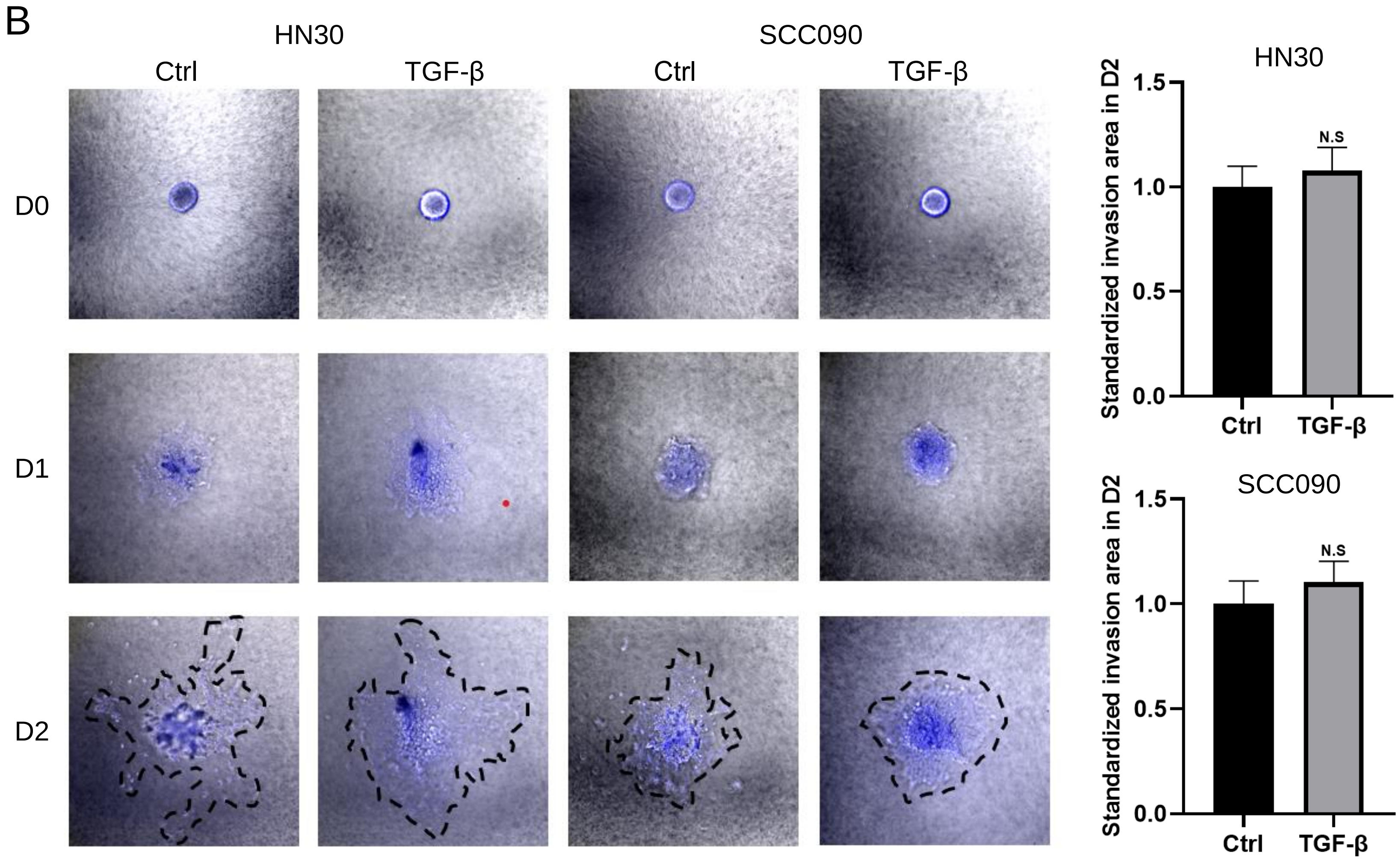

B
HN30
SCC090
Ctrl
TGF-β
Ctrl
TGF-β
D0
D1
D2
HN30
Standardized invasion area in D2
1.5
1.0
0.5
0.0
N.S
Ctrl
TGF-β
SCC090
Standardized invasion area in D2
1.5
1.0
0.5
0.0
N.S
Ctrl
TGF-β

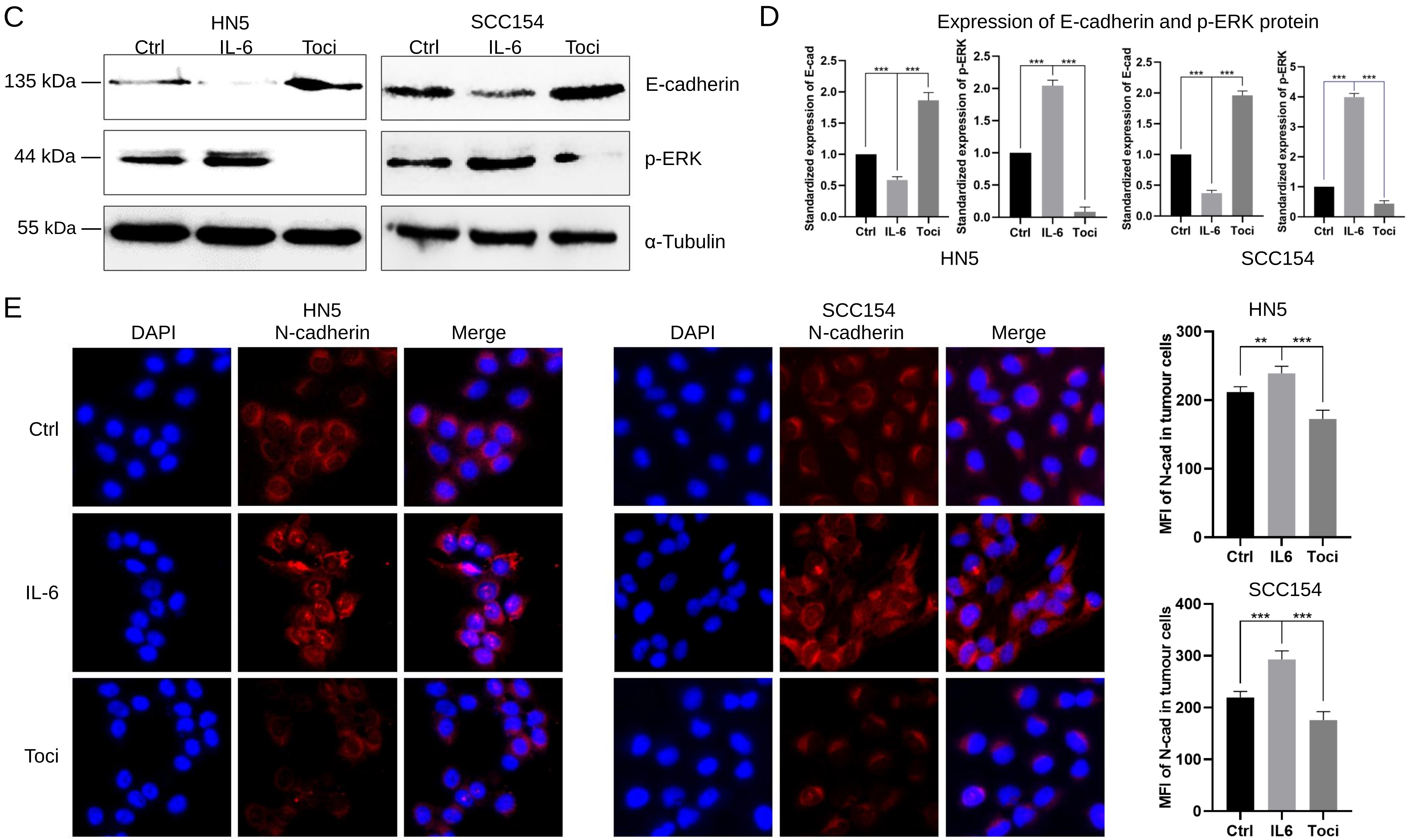
C
HN5
SCC154
Ctrl
IL-6
Toci
135 kDa
44 kDa
55 kDa
E-cadherin
p-ERK
α-Tubulin
D
Expression of E-cadherin and p-ERK protein
Standardized expression of E-cad
Standardized expression of p-ERK
HN5
SCC154
E
DAPI
N-cadherin
Merge
MFI of N-cad in tumour cells
IL6

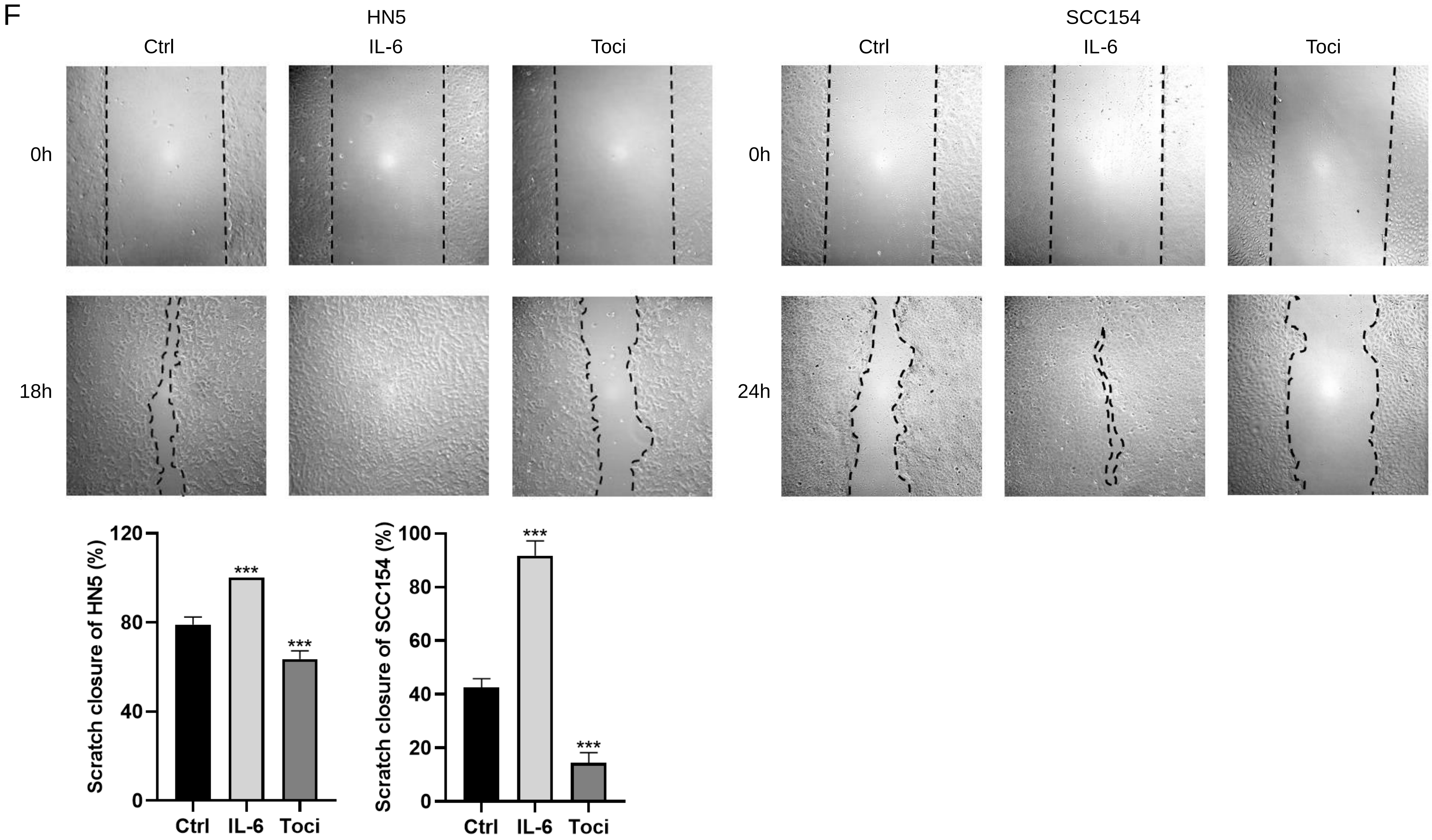

F
HN5
SCC154
Ctrl
IL-6
Toci
0h
18h
24h
Scratch closure of HN5 (%)
Scratch closure of SCC154 (%)
***
0
40
80
120
20
60
100

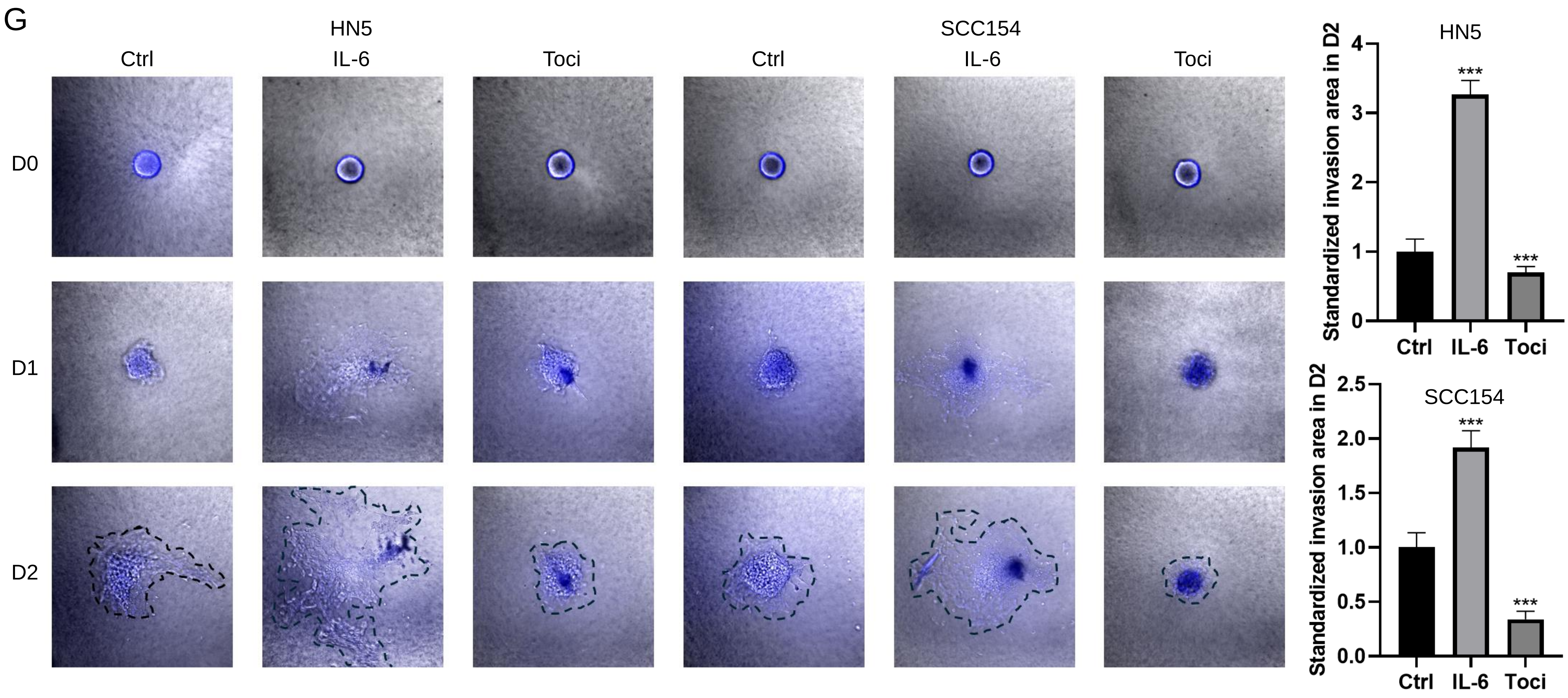
G
HN5
SCC154
Ctrl
IL-6
Toci
Ctrl
IL-6
Toci
D0
D1
D2
HN5
Standardized invasion area in D2
0
1
2
3
4
***
***
Ctrl
IL-6
Toci
SCC154
Standardized invasion area in D2
0.0
0.5
1.0
1.5
2.0
2.5
***
***
Ctrl
IL-6
Toci

H

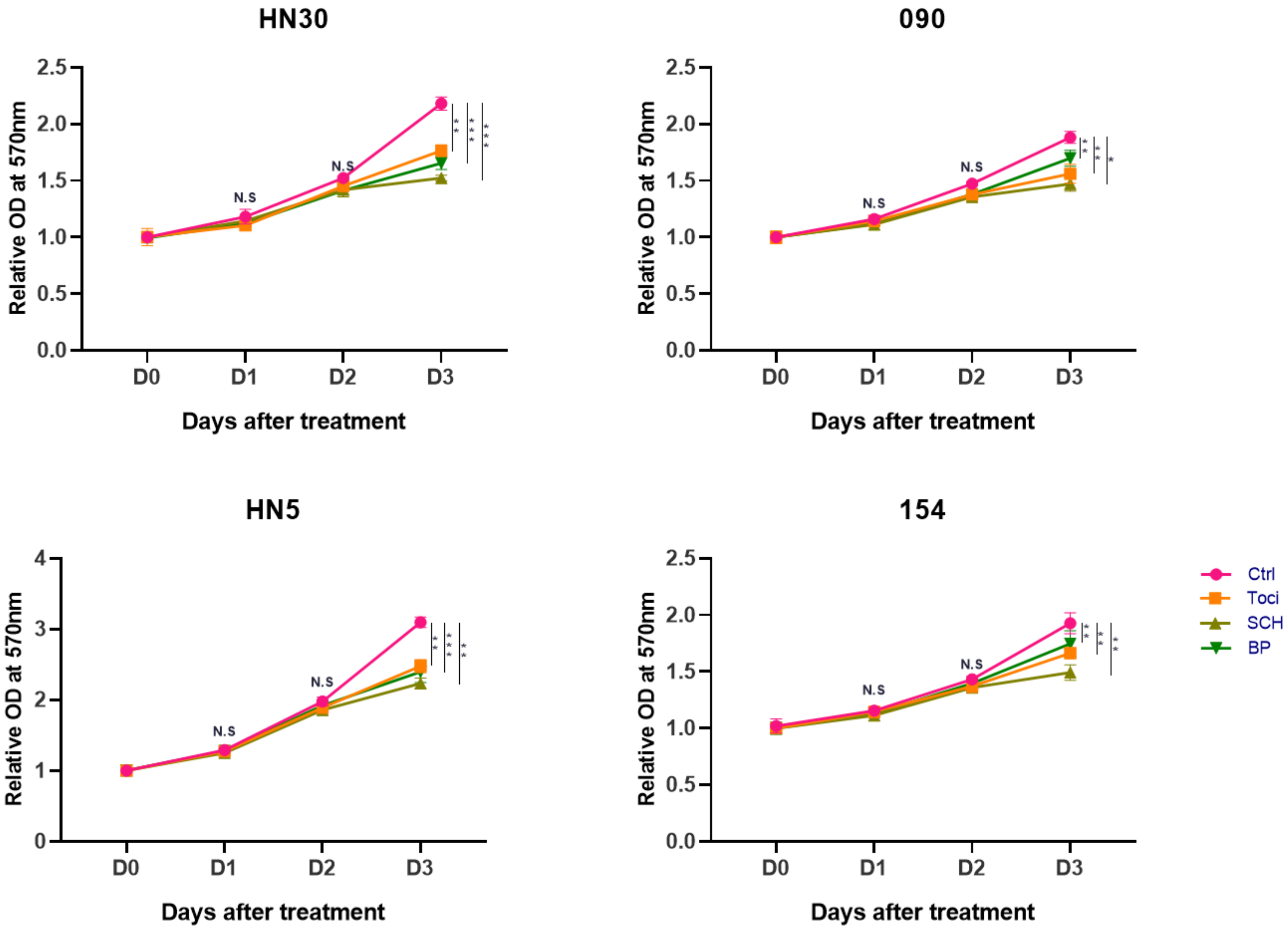
HN30
090
HN5
154
Relative OD at 570nm
Days after treatment
D0
D1
D2
D3
N.S
Ctrl
Toci
SCH
BP

I

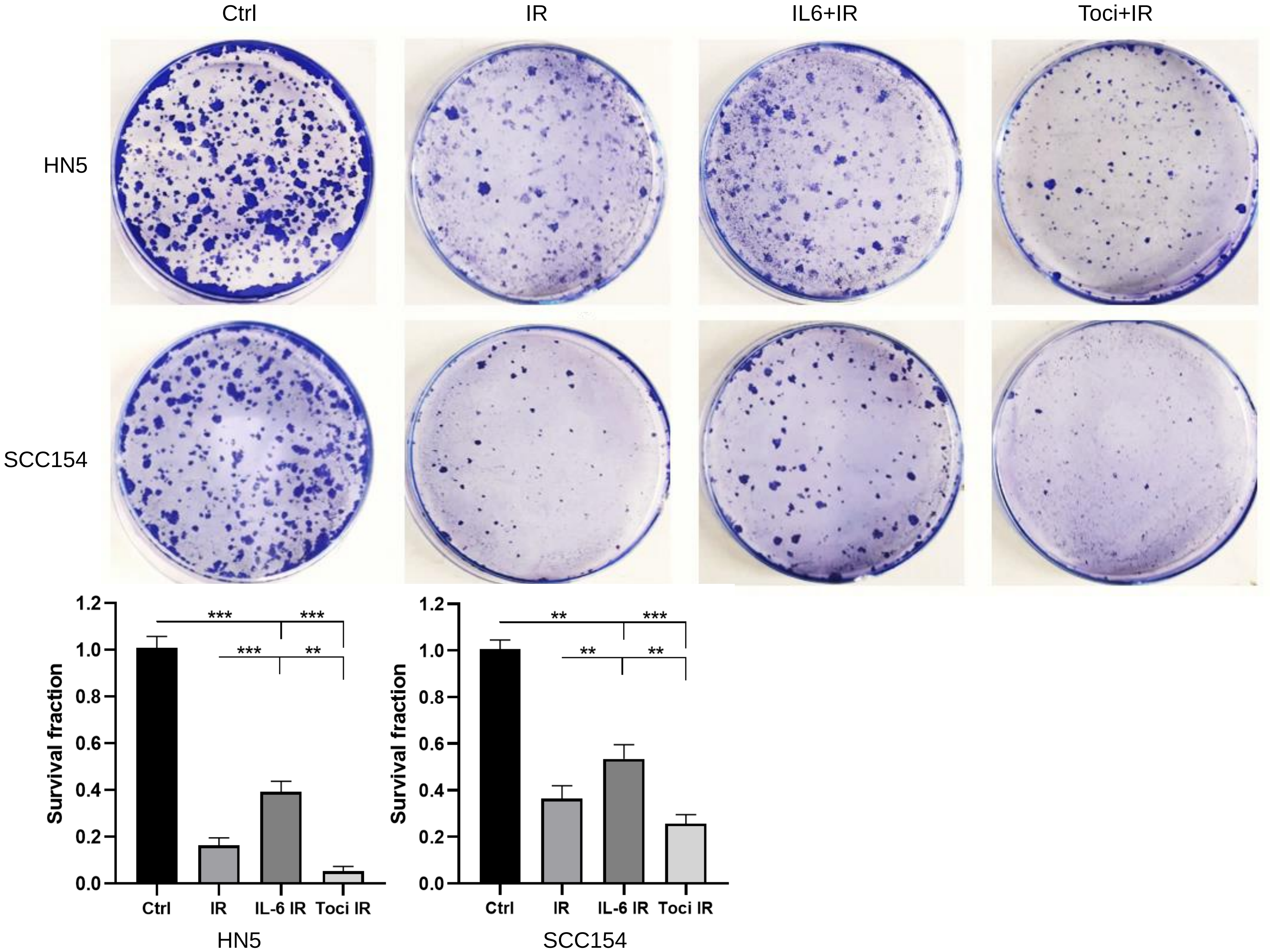
Ctrl
IR
IL6+IR
Toci+IR
HN5
SCC154
Survival fraction
1.2
1.0
0.8
0.6
0.4
0.2
0.0
***
***
***
**
Ctrl
IR
IL-6 IR
Toci IR
HN5
**
***
**
**
SCC154

# Figure S3

MAPK/ERK pathway is the main downstream responder triggered by IL-6 treatment to induce EMT and radioresistance

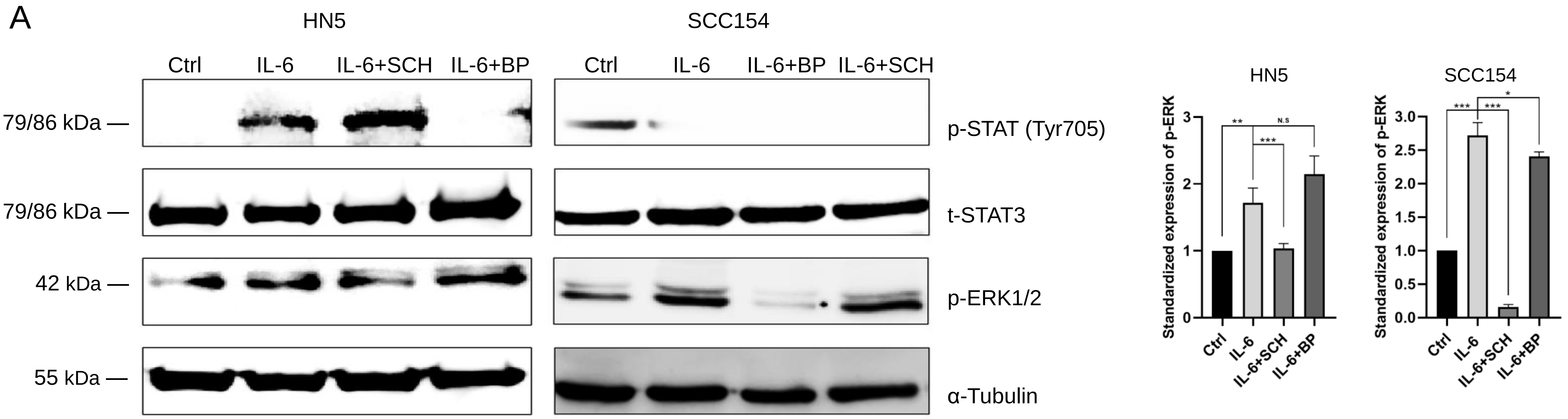
A
HN5
SCC154
Ctrl
IL-6
IL-6+SCH
IL-6+BP
Ctrl
IL-6
IL-6+BP
IL-6+SCH
79/86 kDa —
79/86 kDa —
42 kDa —
55 kDa —
p-STAT (Tyr705)
t-STAT3
p-ERK1/2
α-Tubulin
HN5
SCC154
Standardized expression of p-ERK
Ctrl
IL-6
IL-6+SCH
IL-6+BP
**
N.S
***
*

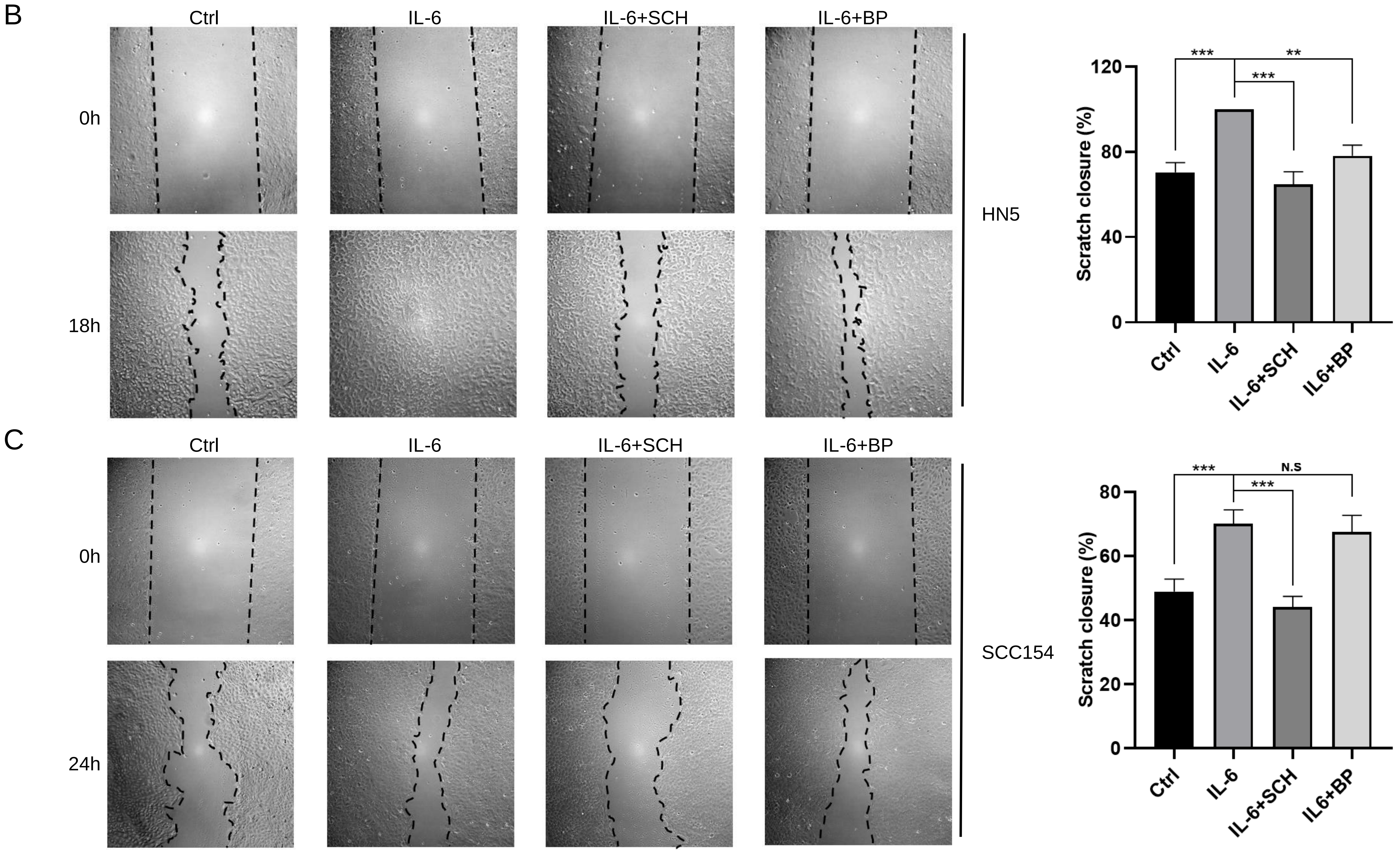
B
Ctrl
IL-6
IL-6+SCH
IL-6+BP
0h
18h
HN5
Scratch closure (%)
120
80
40
0
***
**
***
Ctrl
IL-6
IL-6+SCH
IL6+BP
C
Ctrl
IL-6
IL-6+SCH
IL-6+BP
0h
24h
SCC154
Scratch closure (%)
80
60
40
20
0
***
N.S
***
Ctrl
IL-6
IL-6+SCH
IL6+BP

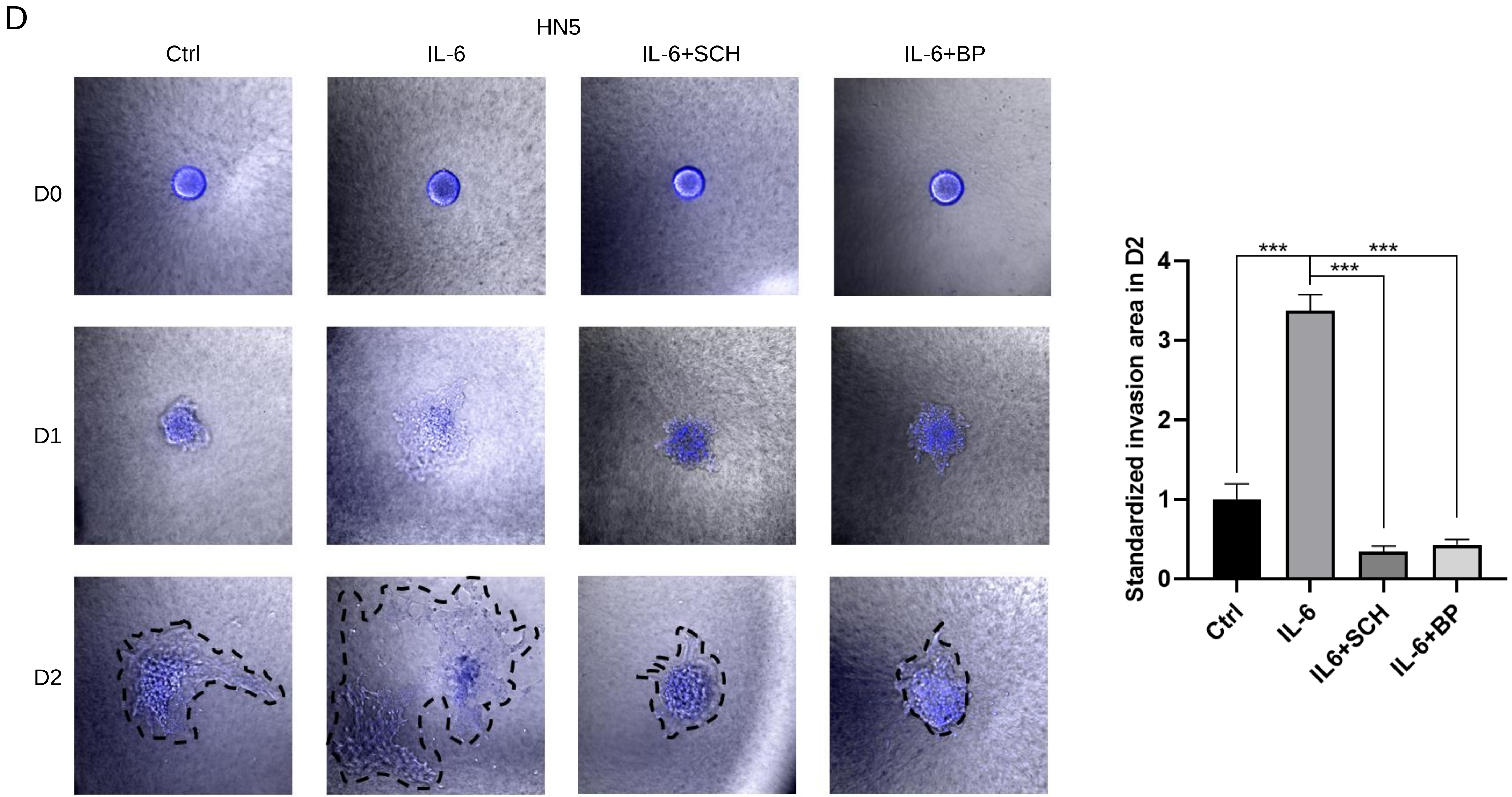
D
HN5
Ctrl
IL-6
IL-6+SCH
IL-6+BP
D0
D1
D2
Standardized invasion area in D2
***
***
***
0
1
2
3
4
Ctrl
IL-6
IL6+SCH
IL-6+BP

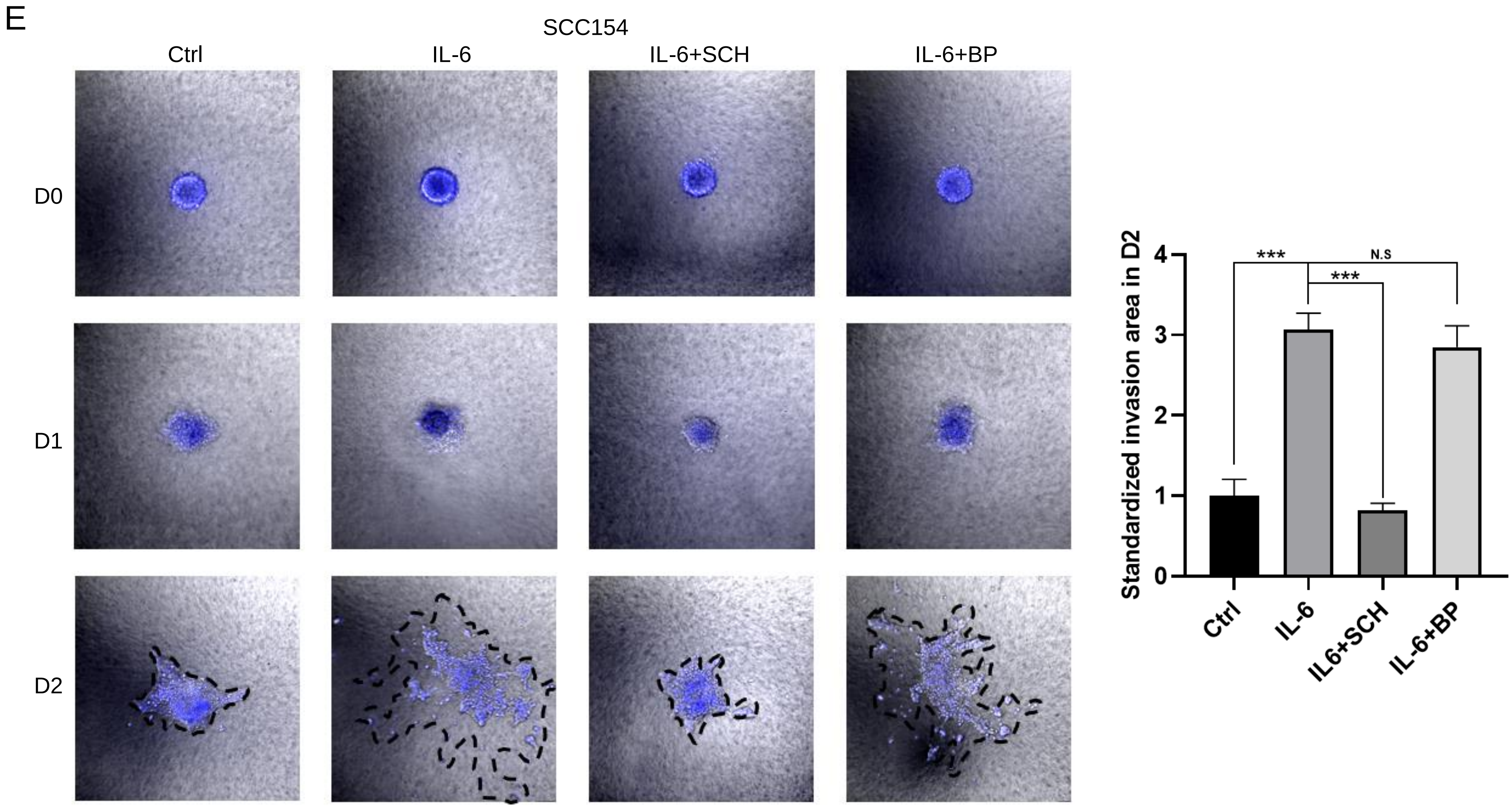
E
SCC154
Ctrl
IL-6
IL-6+SCH
IL-6+BP
D0
D1
D2
Standardized invasion area in D2
0
1
2
3
4
***
***
N.S
Ctrl
IL-6
IL6+SCH
IL-6+BP

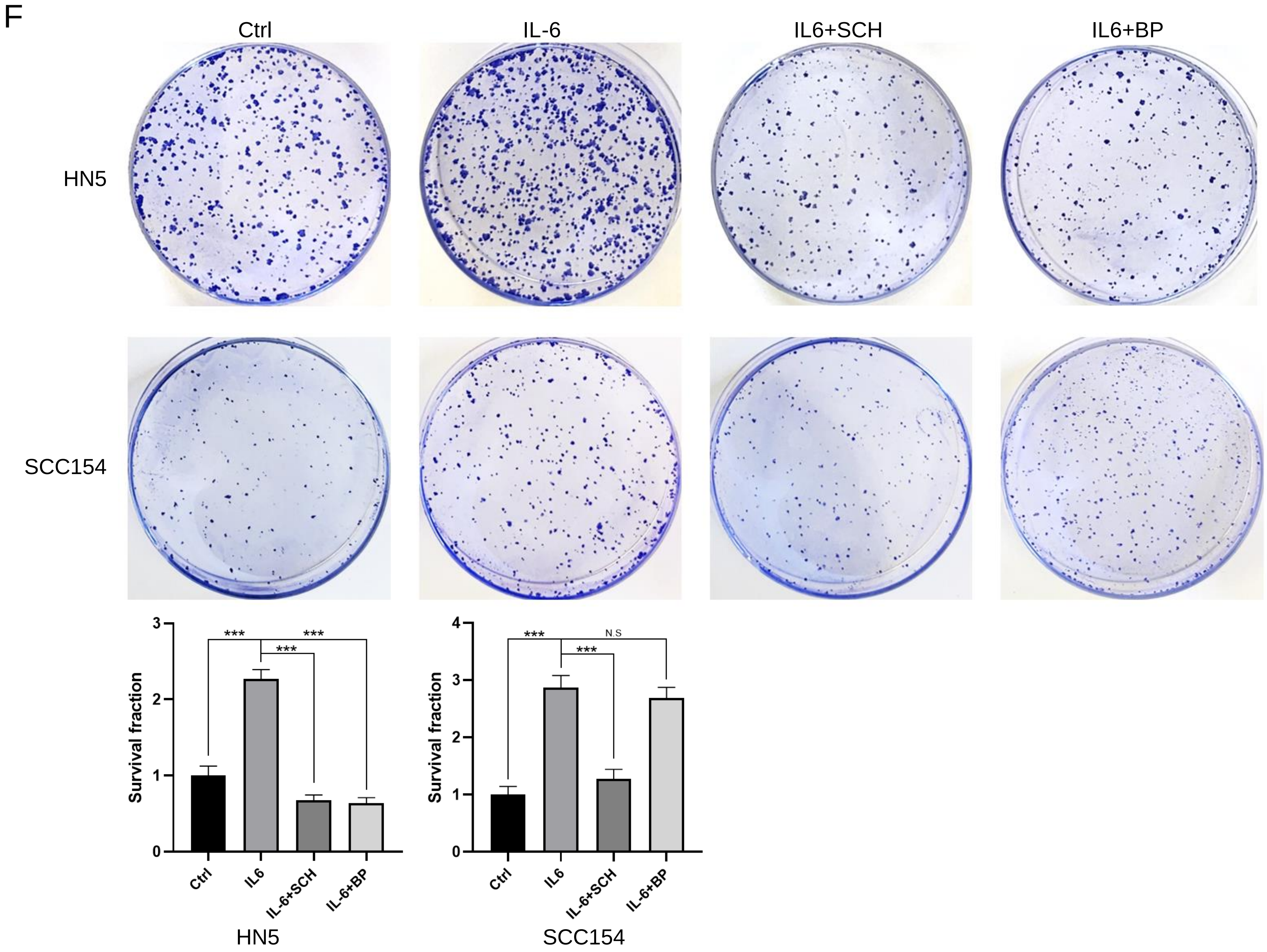
F
Ctrl
IL-6
IL6+SCH
IL6+BP
HN5
SCC154
Survival fraction
***
***
***
N.S
Ctrl
IL6
IL-6+SCH
IL-6+BP
HN5
SCC154

# Figure S4

Blocking the IL-6 receptor or MAPK/ERK pathway eliminates the effects of fibroblast-derived IL-6 on EMT and radioresistance.

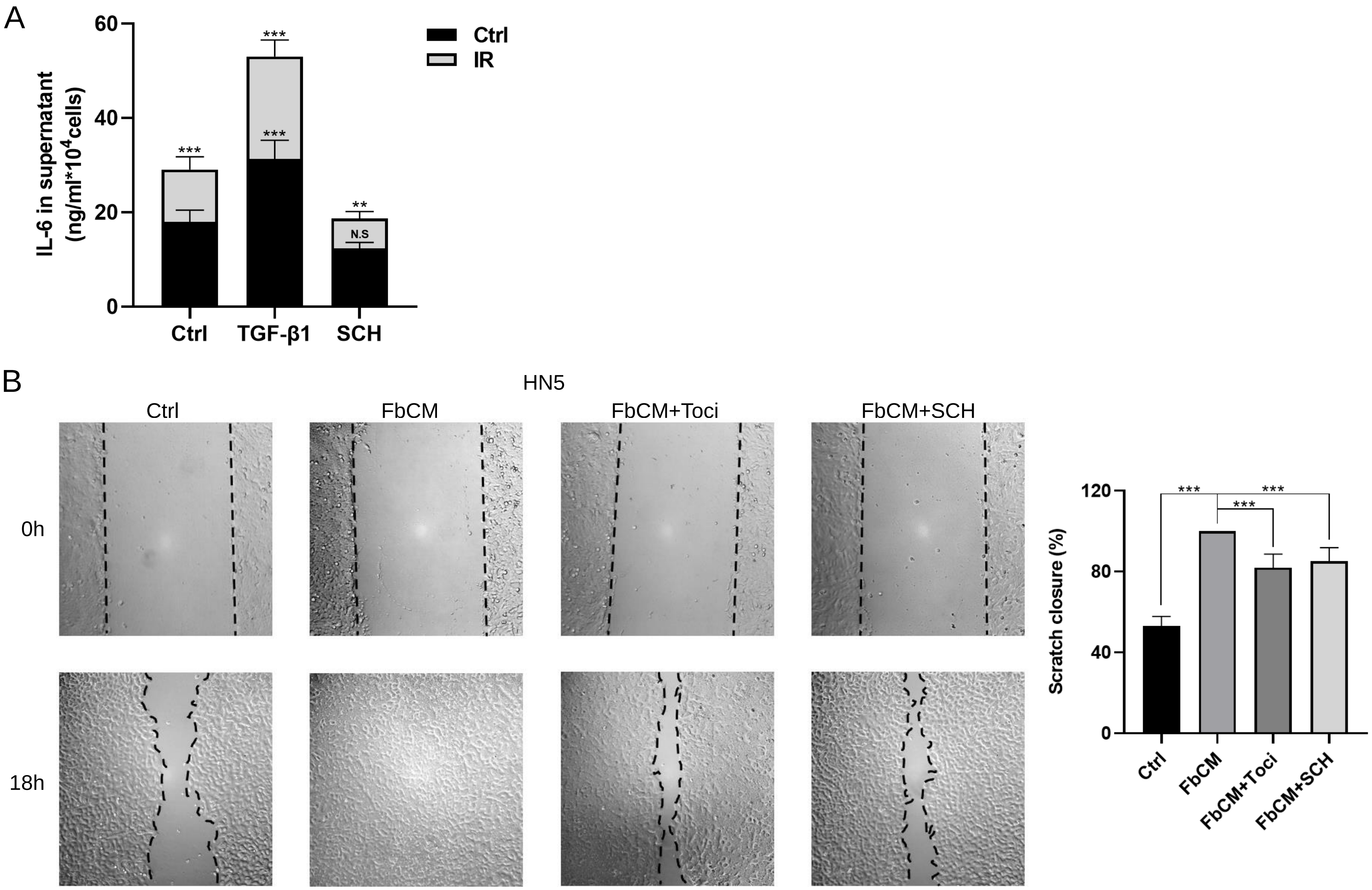
A
IL-6 in supernatant (ng/ml*10^4cells)
60
40
20
0
Ctrl
IR
***
***
***
**
N.S
Ctrl
TGF-β1
SCH
B
HN5
Ctrl
FbCM
FbCM+Toci
FbCM+SCH
0h
18h
Scratch closure (%)
120
80
40
0
***
***
***
Ctrl
FbCM
FbCM+Toci
FbCM+SCH

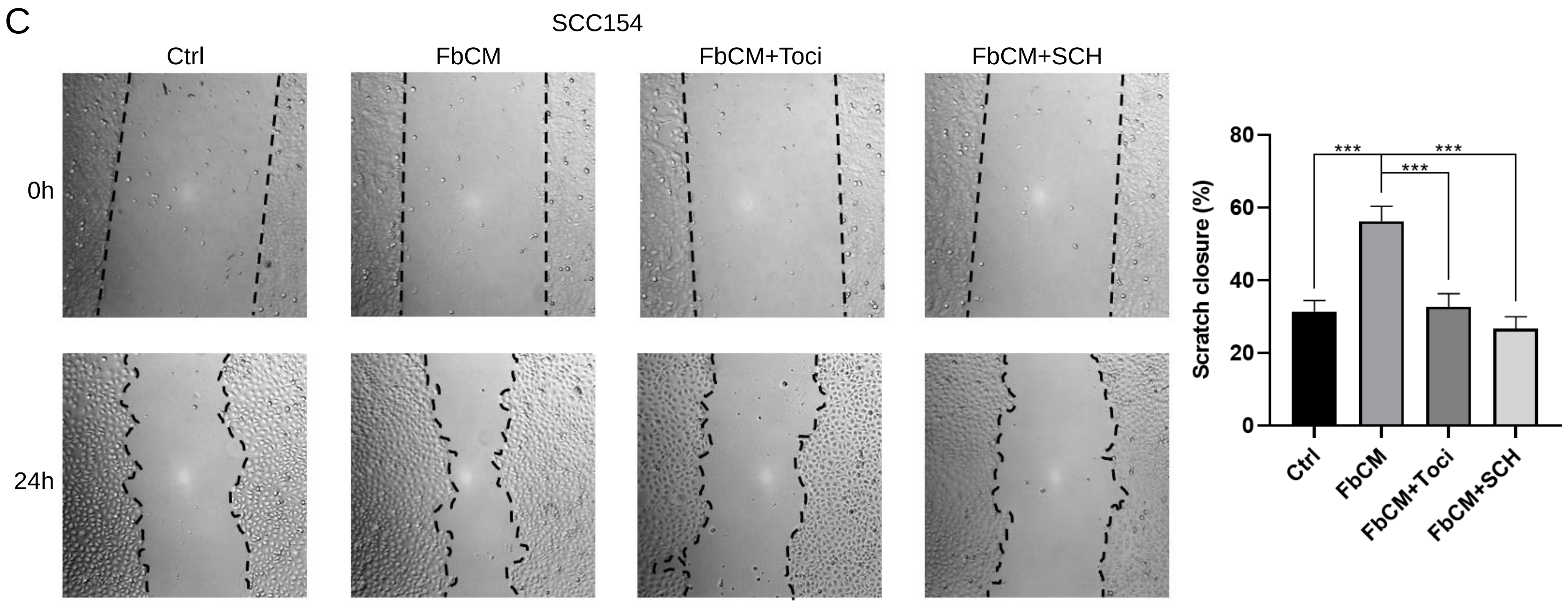
C
SCC154
Ctrl
FbCM
FbCM+Toci
FbCM+SCH
0h
24h
Scratch closure (%)
80
60
40
20
0
***
***
***
Ctrl
FbCM
FbCM+Toci
FbCM+SCH

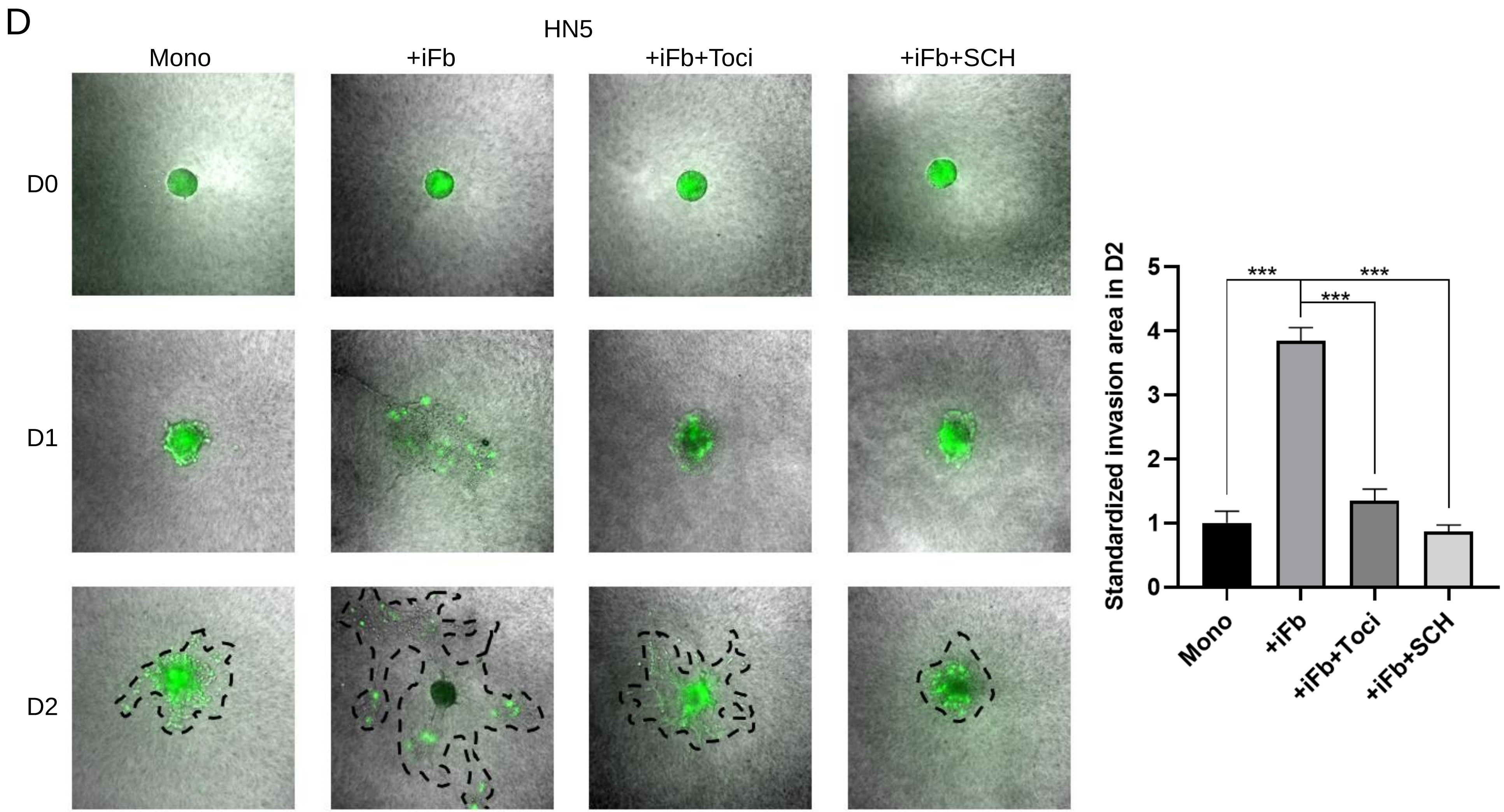
D
HN5
Mono
+iFb
+iFb+Toci
+iFb+SCH
D0
D1
D2
Standardized invasion area in D2
***
***
***
0
1
2
3
4
5
Mono
+iFb
+iFb+Toci
+iFb+SCH

E

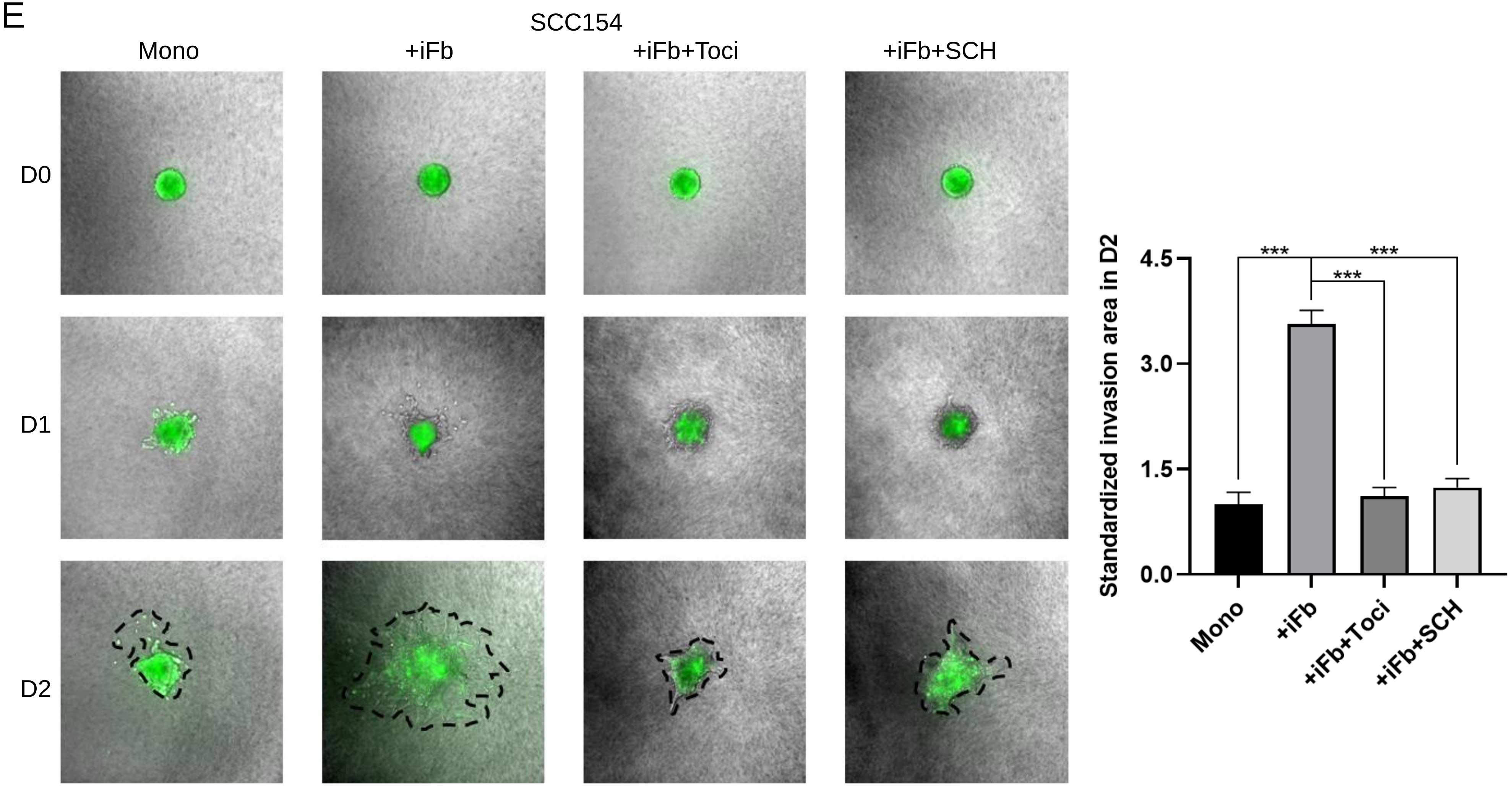
SCC154
Mono
+iFb
+iFb+Toci
+iFb+SCH
D0
D1
D2
Standardized invasion area in D2
4.5
3.0
1.5
0.0
***
***
***
Mono
+iFb
+iFb+Toci
+iFb+SCH

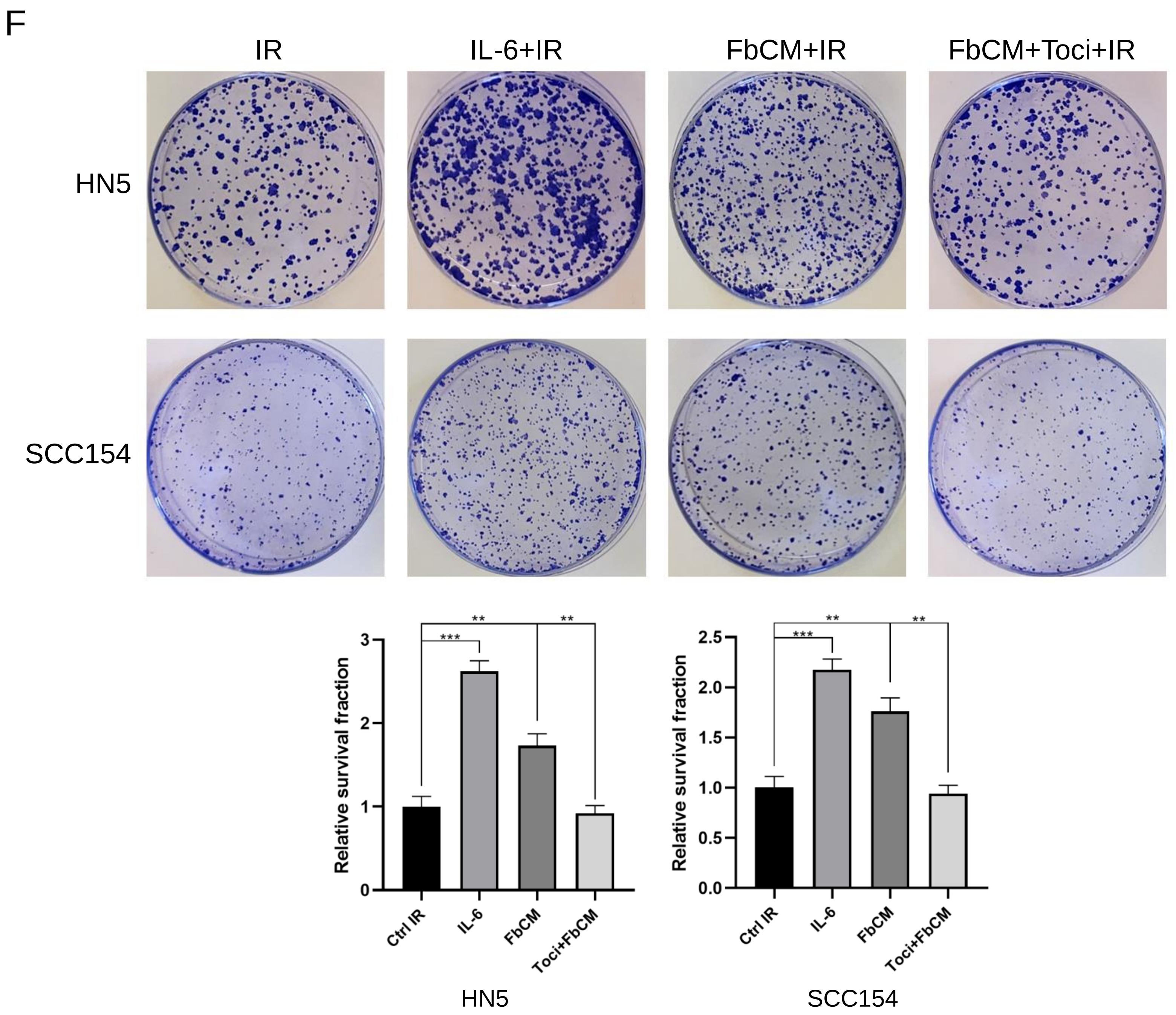
F
IR
IL-6+IR
FbCM+IR
FbCM+Toci+IR
HN5
SCC154
Relative survival fraction
Ctrl IR
IL-6
FbCM
Toci+FbCM
HN5
SCC154

# Figure S5

Validation of downstream responses and sources of IL-6 in HNSCC through bioinformatics analysis

A

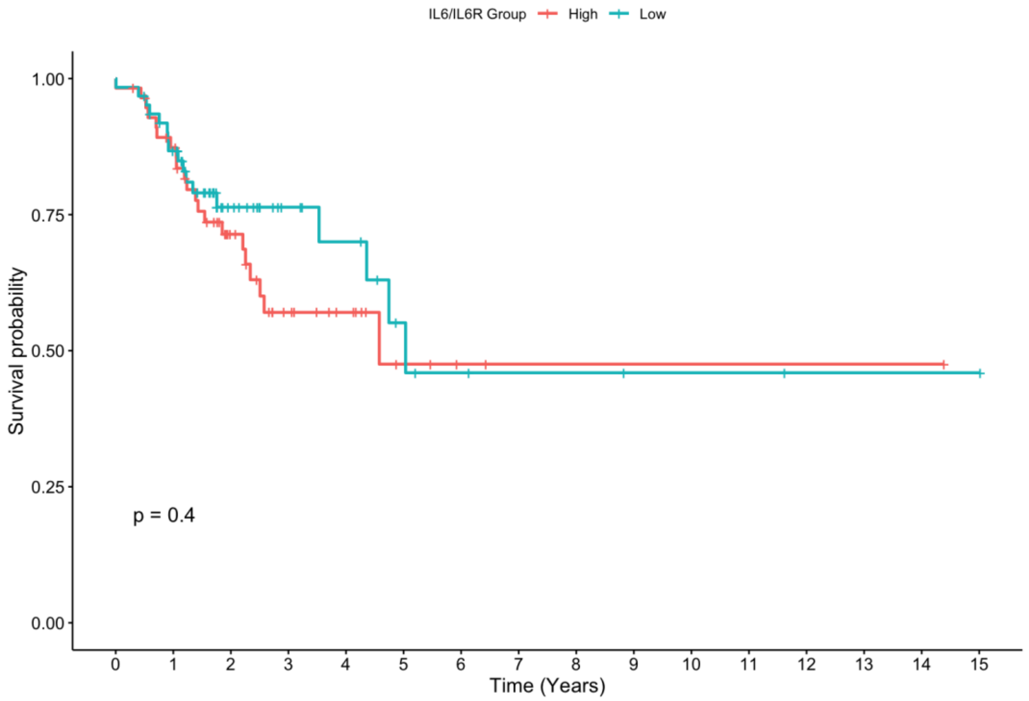
IL6/IL6R Group
High
Low
Survival probability
1.00
0.75
0.50
0.25
0.00
p = 0.4
0 1 2 3 4 5 6 7 8 9 10 11 12 13 14 15
Time (Years)

B

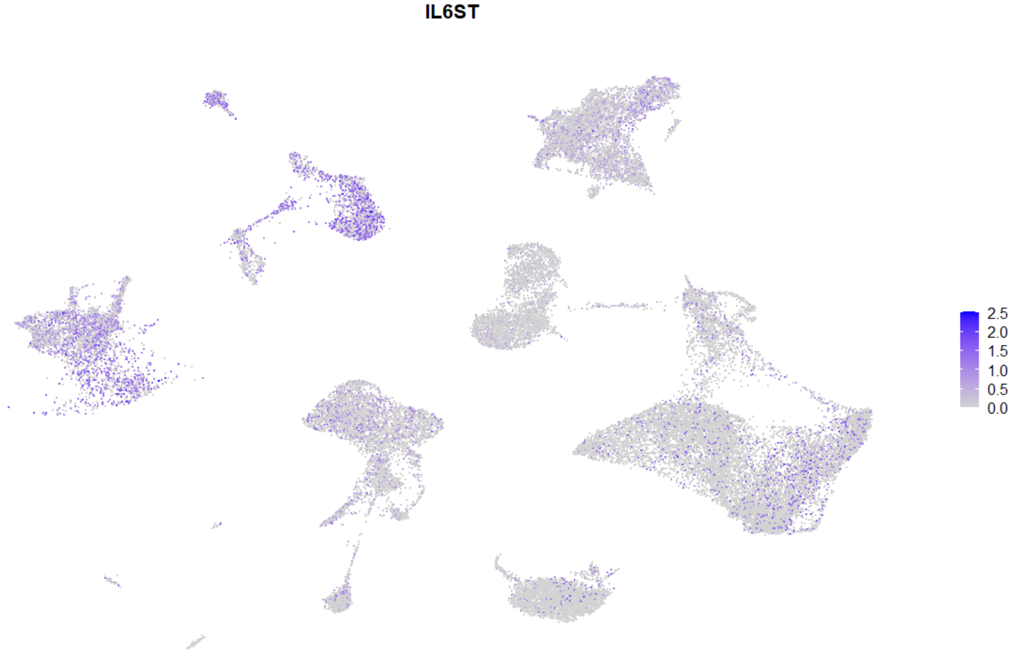
IL6ST
2.5
2.0
1.5
1.0
0.5
0.0

# Figure S6

Increased expression of EMT markers, migration and invasion in RR cell lines

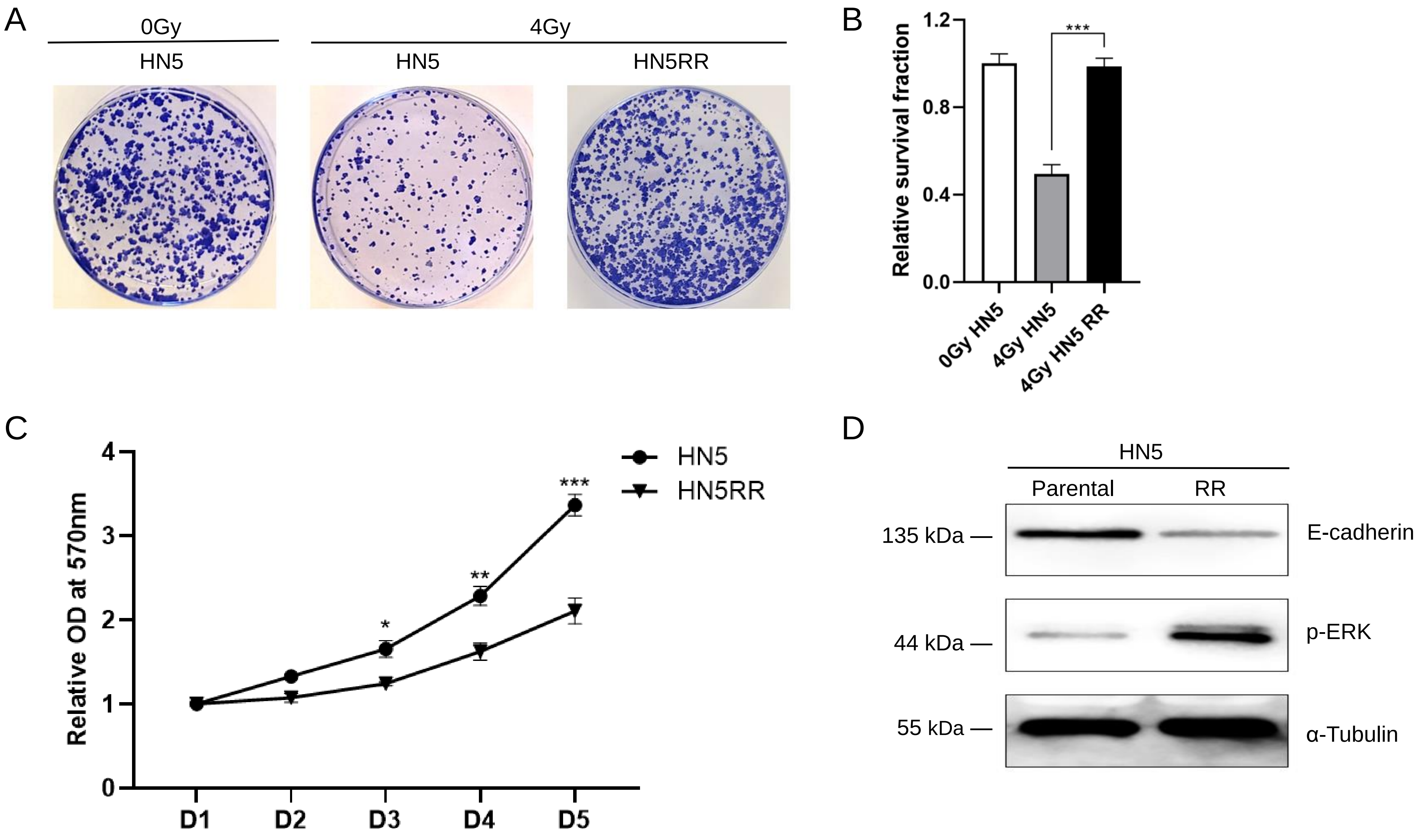
A
0Gy
4Gy
HN5
HN5
HN5RR
B
Relative survival fraction
1.2
0.8
0.4
0.0
***
0Gy HN5
4Gy HN5
4Gy HN5 RR
C
Relative OD at 570nm
4
3
2
1
0
HN5
HN5RR
*
**
***
D1
D2
D3
D4
D5
D
HN5
Parental
RR
135 kDa —
E-cadherin
44 kDa —
p-ERK
55 kDa —
α-Tubulin

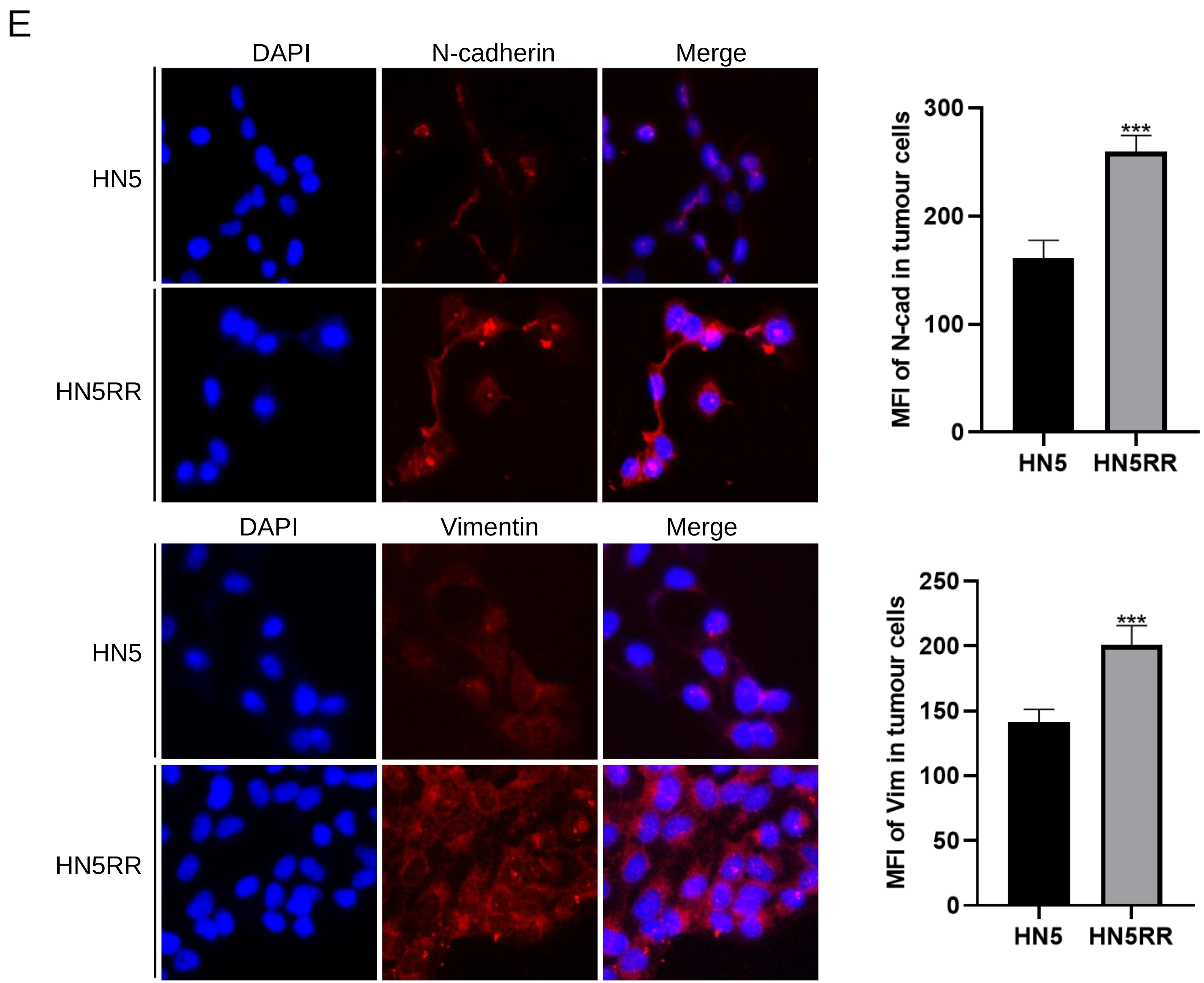
E
DAPI
N-cadherin
Merge
HN5
HN5RR
MFI of N-cad in tumour cells
300
200
100
0
***
HN5
HN5RR
DAPI
Vimentin
Merge
HN5
HN5RR
MFI of Vim in tumour cells
250
200
150
100
50
0
***
HN5
HN5RR

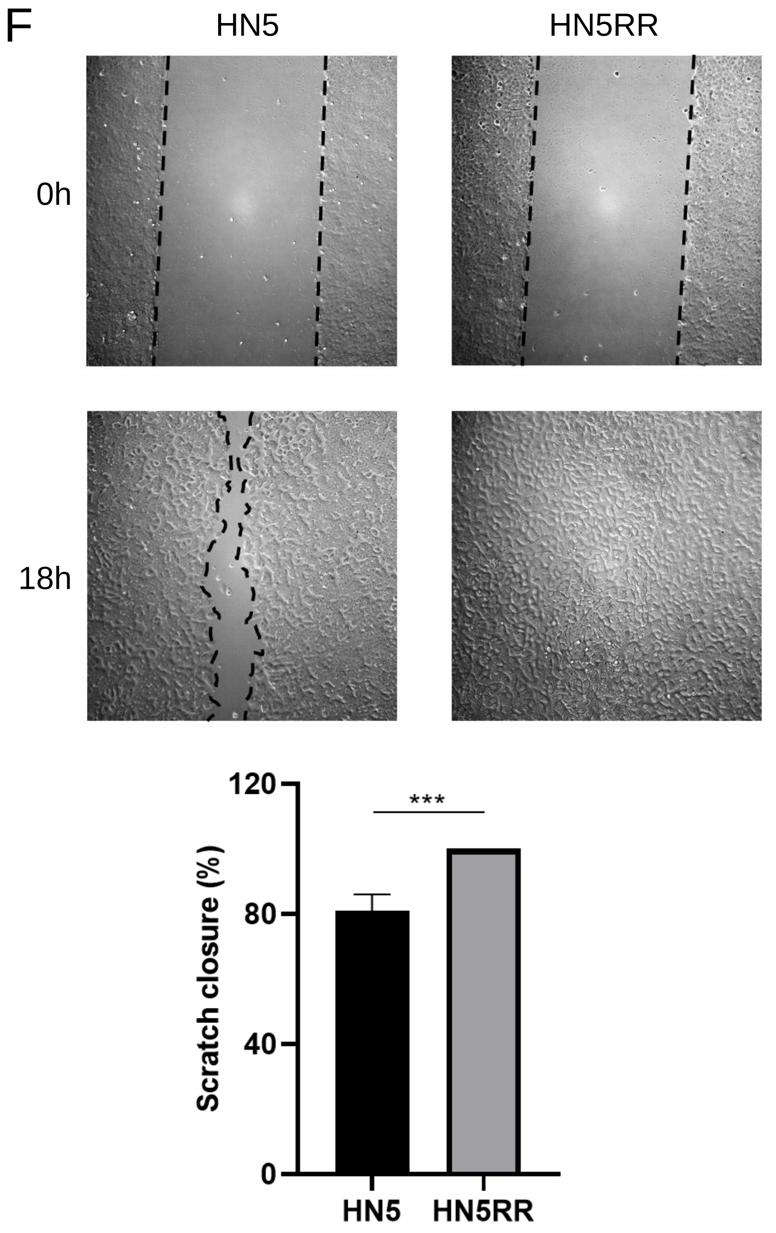
F
HN5
HN5RR
0h
18h
120
80
40
0
Scratch closure (%)
***
HN5
HN5RR

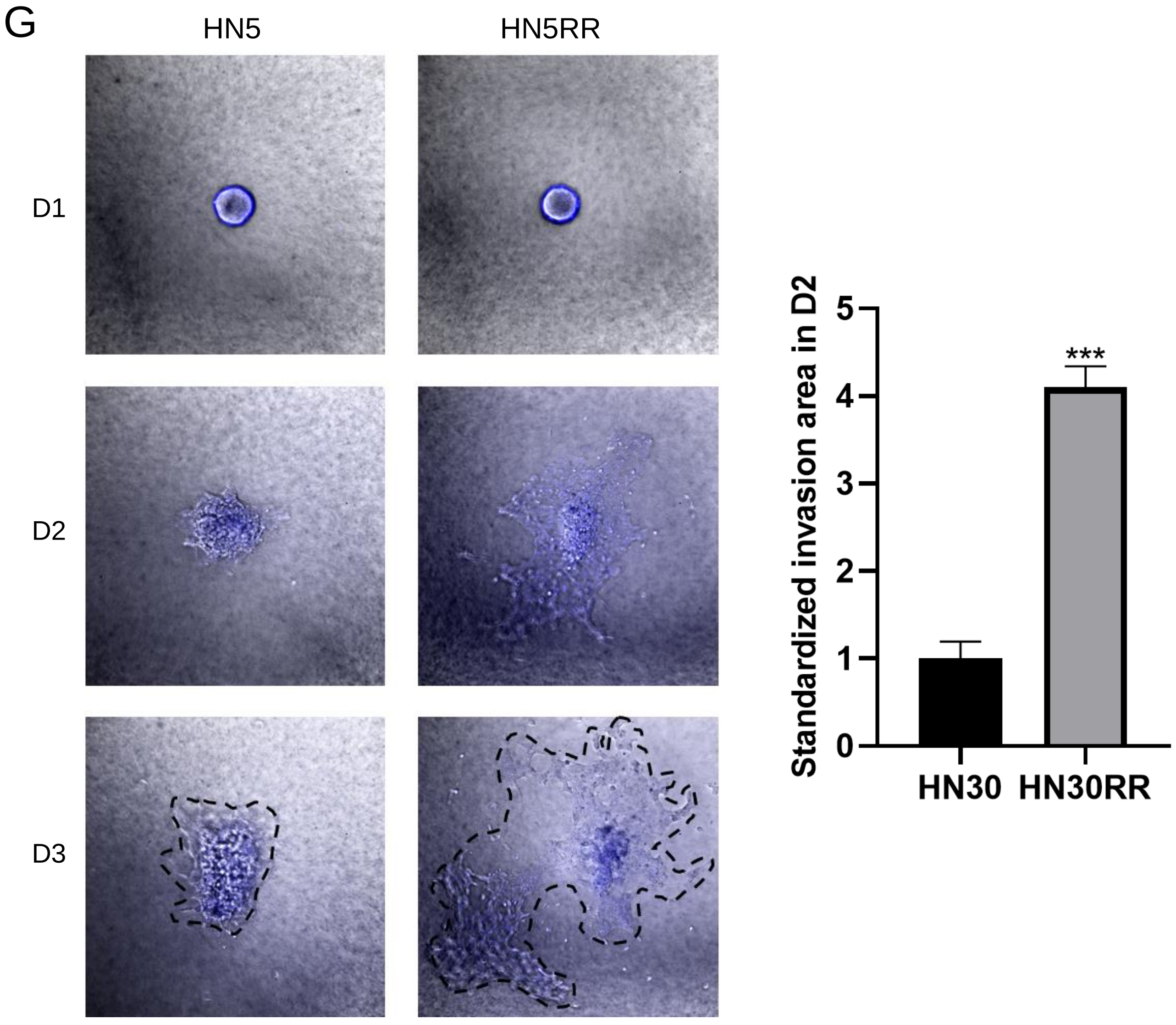
G
HN5
HN5RR
D1
D2
D3
Standardized invasion area in D2
5
4
3
2
1
0
***
HN30
HN30RR

# Figure S7

Blocking the IL-6R or MAPK/ERK pathway reverses the increased radioresistance, migration and invasion in RR cell lines.

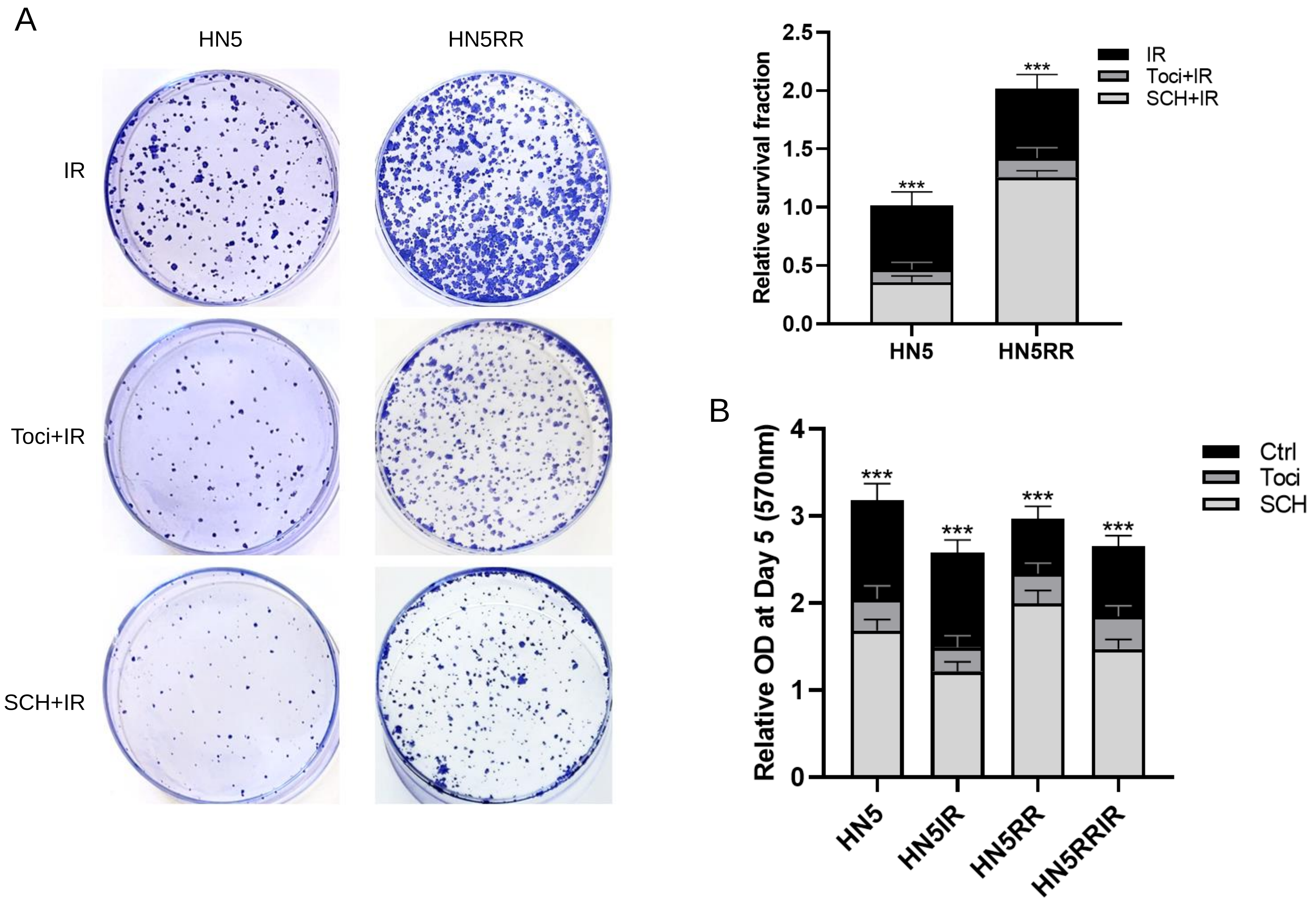
A
HN5
HN5RR
IR
Toci+IR
SCH+IR
Relative survival fraction
2.5
2.0
1.5
1.0
0.5
0.0
***
***
HN5
HN5RR
IR
Toci+IR
SCH+IR
B
Relative OD at Day 5 (570nm)
4
3
2
1
0
***
***
***
***
HN5
HN5IR
HN5RR
HN5RRIR
Ctrl
Toci
SCH

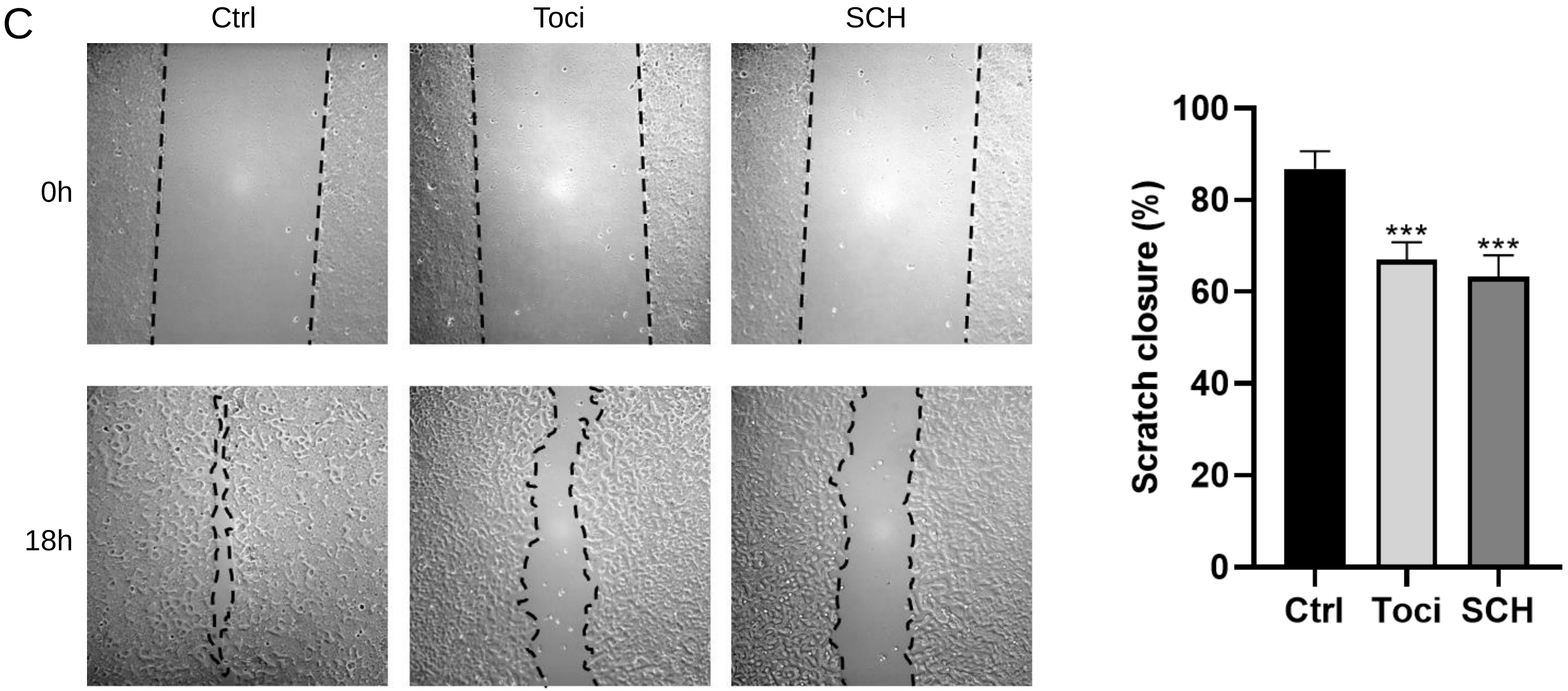
C
Ctrl
Toci
SCH
0h
18h
Scratch closure (%)
100
80
60
40
20
0
***
***
Ctrl
Toci
SCH

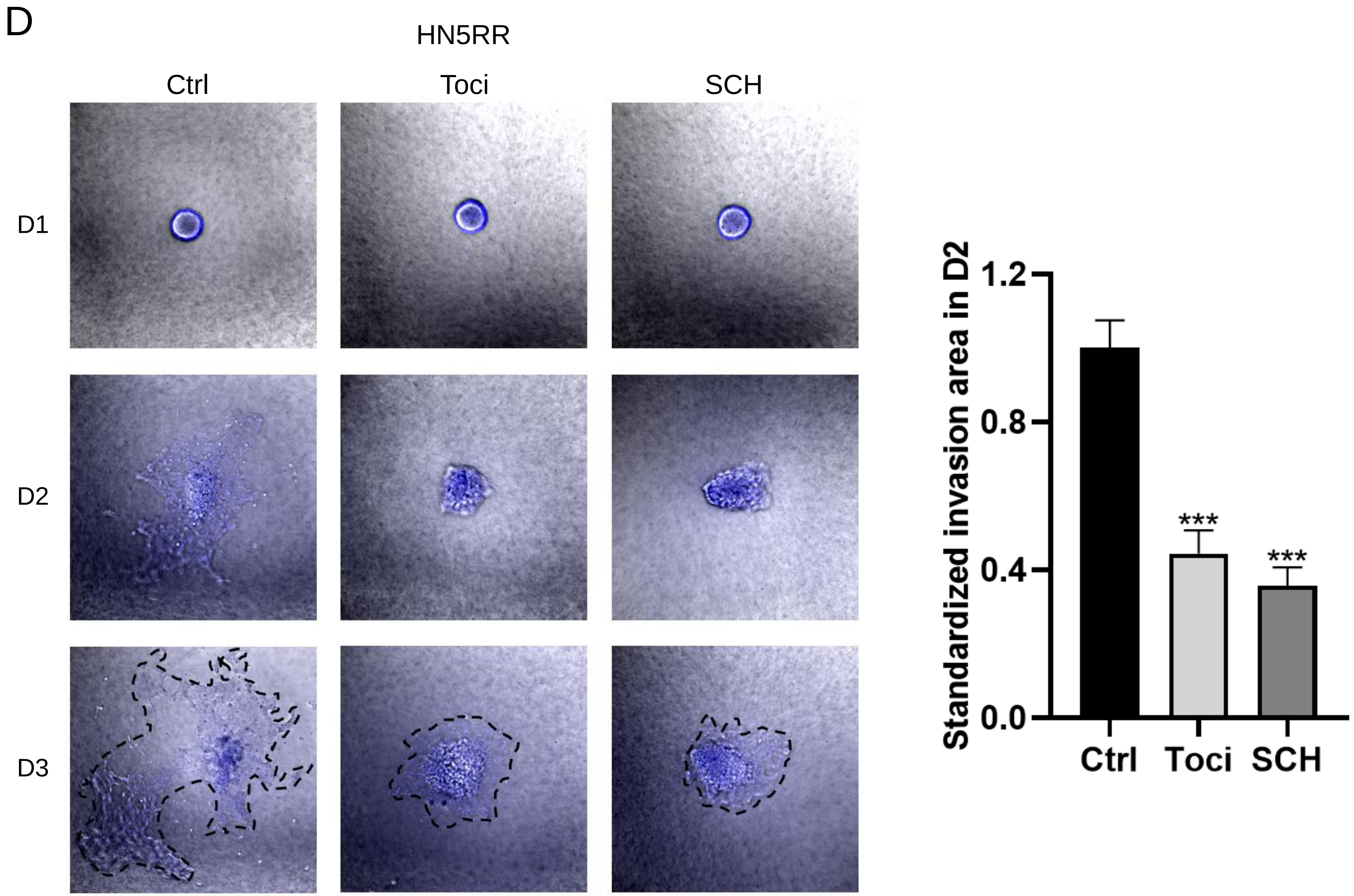
D
HN5RR
Ctrl
Toci
SCH
D1
D2
D3
Standardized invasion area in D2
1.2
0.8
0.4
0.0
***
***
Ctrl
Toci
SCH
