# Supplementary material for: Targeting Fibroblast-Derived Interleukin 6: A Strategy to Overcome Epithelial-Mesenchymal Transition and Radioresistance in Head and Neck Cancer
Source: Cancers (Basel). 2025 Jan 15;17(2):267. doi: 10.3390/cancers17020267 (PMC11763410; doi:10.3390/cancers17020267)
Supplement: Supplementary file 1 [file cancers-17-00267-s001.zip › Table S1-3.pdf]

Table S1. Table of the markers used to identify different clusters in scRNA-Seq data.

Cluster	Cell type	Markers
0	Fibroblasts	FAP, PDPN, COL1A2, DCN, COL3A1, COL6A1
1	T-Cells	CD2, CD3D, CD3E, CD3G
2	T-Cells	CD2, CD3D, CD3E, CD3G
3	Malignant Cells	KRT14, KRT17, KRT6A, KRT5, KRT19, KRT8, KRT16, KRT18, KRT6B, KRT15, KRT6C, KRTCAP3, EPCAM, SFN
4	Macrophages	CD14, CD163, CD68, FCGR2A, CSF1R
5	B/Plasma Cells	SLAMF7, CD79A, BLNK, FCRL5
6	Endothelial Cells	SLAMF7, CD79A, BLNK, FCRL5
7	CAFs	FAP, PDPN, CTGF, MMP2
8	B/Plasma Cells	SLAMF7, CD79A, BLNK, FCRL5
9	T-Cells	CD2, CD3D, CD3E, CD3G
10	B/Plasma Cells	SLAMF7, CD79A, BLNK, FCRL5
11	Dendritic Cells	CD40, CD80, CD83, CCR7
12	Myofibroblasts	ACTA2, MYLK, MYL9
13	Lymphatic endothelial cells	CCL21, MMRN1, ART4
14	Unknown	
15	Skeletal myocytes	MYH2, NRAP, ACTA1
16	Mast Cells	CMA1, MS4A2, TPSAB1, TPSB2
17	Malignant Cells	KRT14, KRT17, KRT6A, KRT5, KRT19, KRT8, KRT16, KRT18, KRT6B, KRT15, KRT6C, KRTCAP3, EPCAM, SFN

Table S2. Top 5 Gene Sets in GSEA Analysis Between IL-6/IL-6R 'High' and 'Low' Groups Using the Hallmark Gene Set.

Table of the 5 gene sets with the best adjusted p-value (P-adj) in the GSEA Pre-ranked analysis between IL6/IL6R 'High' and 'Low' groups using the hallmark gene set. The columns include the pathway name, normalized enrichment score (NES), adjusted p-value (P-adj), and the size of the gene set. The positive NES values, highlighted in red, indicate enrichment in the IL-6/IL-6R 'High' groups.

**Pathway**	**NES**	**P-adj**	**Size**
HALLMARK_EPITHELIAL_MESENCHYMAL_TRANSITION	2.903	1.22×10^{-35}	200
HALLMARK_TNFA_SIGNALING_VIA_NFKB	2.861	1.23×10^{-32}	199
HALLMARK_INFLAMMATORY_RESPONSE	2.816	2.30×10^{-30}	199
HALLMARK_KRAS_SIGNALING_UP	2.474	4.36×10^{-16}	200
HALLMARK_INTERFERON_GAMMA_RESPONSE	2.450	2.60×10^{-15}	199

Table S3. Top 10 Differential Gene Sets Between IL-6/IL-6R 'High' and 'Low' Groups Using the KEGG Gene Set.

Table of the 10 gene sets with the best adjusted p-value in the GSEA Pre-ranked analysis between IL6/IL6R 'High' and 'Low' groups using the KEGG gene set. The columns include the pathway name, normalized enrichment score (NES), adjusted p-value (P-adj), and the size of the gene set. The positive NES values (in red) indicate an enrichment in the IL-6/IL-6R 'High' group, and the negatives NES values (in blue) indicates an enrichment in the IL-6/IL-6R 'Low' group.

**Pathway**	**NES**	**P-adj**	**Size**
KEGG_FOCAL_ADHESION	2.740	5.77×10^{-26}	198
KEGG_CYTOKINE_CYTOKINE_RECEPTOR_INTERACTION	2.544	3.12×10^{-25}	254
KEGG_JAK_STAT_SIGNALING_PATHWAY	2.662	1.81×10^{-17}	147
KEGG_PATHWAYS_IN_CANCER	2.275	2.79×10^{-17}	325
KEGG_CHEMOKINE_SIGNALING_PATHWAY	2.478	7.76×10^{-16}	187
KEGG_OXIDATIVE_PHOSPHORYLATION	−2.069	6.09×10^{-15}	131
KEGG_REGULATION_OF_ACTIN_CYTOSKELETON	2.356	8.52×10^{-13}	212
KEGG_HEMATOPOIETIC_CELL_LINEAGE	2.649	6.18×10^{-12}	84
KEGG_PARKINSONS_DISEASE	−1.940	2.24×10^{-10}	129
KEGG_MAPK_SIGNALING_PATHWAY	2.032	6.47×10^{-10}	267
